# Investigation of the thermal deconstruction of β–β′ and 4-O-5 linkages in lignin model oligomers by density functional theory (DFT)†

**DOI:** 10.1039/d2ra07787f

**Published:** 2023-02-21

**Authors:** Ross W. Houston, Nourredine H. Abdoulmoumine

**Affiliations:** a Department of Biosystems Engineering and Soil Science, University of Tennessee 2506 E. J. Chapman Drive Knoxville TN 37996 USA nabdoulm@utk.edu; b Center for Renewable Carbon, University of Tennessee 2506 Jacob Drive Knoxville TN 37996 USA

## Abstract

Model compounds that represent important substructures in lignin have popularly been used to gain a better understanding of the behavior of lignin during thermal deconstruction, such as fast pyrolysis. The β-O-4 linkage of lignin has previously been the focus of many model compound studies as it is the most prevalent linkage found in native lignin. In this work, two lesser studied linkages, the β–β′ and 4-O-5, were investigated with density functional theory (DFT). Bond dissociation enthalpies (BDEs) were calculated for the relevant bonds along each interunit linkage for two model compounds containing these linkages. Conformational analysis of the first model oligomer has a relative enthalpy difference of 1.55 kcal mol^−1^. For the β–β′ linkage, the alpha carbons had the lowest BDEs of the ring opening reactions due to excessive electron delocalization around the aromatic rings. The bonds of the 4-O-5 linkage had similar BDEs but were appreciably higher than the BDEs for other ether linkages, such as β-O-4 and α-O-4. The higher BDEs of the 4-O-5 bonds is a result of the radical being formed on an aromatic carbon compared to an aliphatic carbon. Our results indicate the ring-opening reactions around the alpha-carbon of the β–β′ linkage would be a major reaction point during thermal deconstruction of the chosen oligomers. This work provides valuable information on the thermal deconstruction behavior of two lesser studied interunit linkages that builds on the authors' previous work, on β-O-4, α-O-4, and β-5 linkages, to develop a library of reaction information for various lignin interunit linkages.

## Abstract 2

Density functional theory (DFT) simulations were performed on two lesser understood linkages in lignin. The C_α_-containing bonds look to be the primary points of reaction along the β–β′ linkage during thermal conversion.

## Graphical abstract

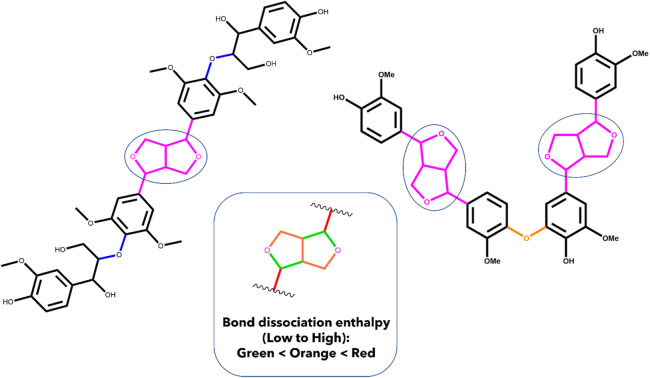

## Introduction

Lignocellulosic biomass is an abundant, renewable resource with the potential to serve as a sustainable alternative to fossil fuel derived products such as liquid transportation fuel and specialty chemicals. Increasing concern over greenhouse gas emission from fossil fuels has sparked interest in understanding how to effectively convert lignocellulosic biomass into value-added materials typically derived from fossil fuels. Fast pyrolysis is a thermochemical conversion process that can create a liquid product, which can potentially be upgraded into transportation fuel and other valuable products.

Lignocellulosic biomass is primarily comprised of three major components: cellulose, hemicellulose, and lignin. Cellulose makes up 40–50 wt% of lignocellulosic biomass and is a linear polymer of glucose monomers joined together through a β-(1,4) glycosidic linkage. It is considered the most abundant naturally occurring polymer in the world.^1–4^ The cellulose found in lignocellulosic biomass is structurally the same regardless of material except for the degree of polymerization. Cellulose has received significant attention in fast pyrolysis reaction studies and its reaction mechanism is consequently more understood relative the other major components.^5–10^ Hemicellulose comprises 20–35 wt% of lignocellulosic biomass and is an amorphous heteropolymer of pentose and hexose sugars.^11,12^ The composition and structure of hemicellulose is dependent on the biomass source; however, it be classified in terms of well-defined polysaccharide types.^13^ Hemicellulose has not been investigated to the extent of cellulose, but mechanistic information about its fast pyrolysis process has been achieved.^11^ Lignin, the last major component of lignocellulosic biomass, constitutes up to 35 wt% of lignocellulosic biomass.^14,15^ Unlike cellulose and hemicellulose, lignin does not have a polysaccharide structure. Instead, lignin is made up of aromatic units and is the most abundant natural source of aromatics in the world.^16^ Lignin is synthesized *via* enzymatic dehydrogenation of three monolignols, *p*-coumaryl alcohol, sinapyl alcohol, and coniferyl alcohol. The resulting phenoxyl radicals are randomly polymerized into a complex and not well-defined three-dimensional structure with a variety of different interunit linkages.^15,17^ Lignin's fast pyrolysis mechanism is less understood compared to cellulose and hemicellulose, owing to its complex and less well-defined structure. The limited knowledge of lignin's fast pyrolysis mechanism leaves a significant knowledge gap in whole lignocellulosic biomass' fast pyrolysis reaction mechanism. Therefore, it is difficult to implement mechanistic reaction schemes into models of biomass fast pyrolysis and has led to the use of lumped kinetic schemes.^19^ Consequently, the overall goal of this study is to improve our understanding of lignin fast pyrolysis by examining the pyrolytic breakdown of specific interunit linkages in model compounds through bond dissociation enthalpies (BDE). The determination of BDEs of lignin interunit linkages provides valuable insights on initial reaction sites and can guide the proposal of future lignin pyrolysis reaction mechanisms using a combination of both computational and experimental approaches.

Despite lignin's unclear native structure, it is still possible to identify prevalent and important linkages found in lignin. We can gain valuable understanding of the reaction behavior of these linkages and substructures using model compounds. Model compounds are smaller, simpler compounds that either contain a significant interunit linkage found in lignin or represent possible substructures of a larger lignin polymer. Most lignin model compound studies have focused on model monomers, such as guaiacol and syringol, and dimers, such as phenethyl phenyl ether (PPE).^20–32^ The kinetic information gathered from monomer and dimer studies has greatly improved our understanding of the thermal degradation behavior of the smallest lignin building blocks; however, due to the complex, non-linear nature of lignin, it is not advisable to assume the kinetics are directly applicable to the larger native lignin structure. Therefore, the knowledge gap for lignin pyrolysis is how to get from model dimers to a native lignin structure. The authors believe it is possible to bridge that gap by increasing the size of the model compounds and begin to investigate oligomers and other important lignin substructures. Recently, there have been studies that have explored the thermal decomposition behaviors of larger, linear oligomeric species.^33^ The author's previous work investigated a model tetramer containing β-O-4, α-O-4, and β-5 interunit linkages and found the trends between dimer and oligomer for each linkage hold true. The C–O bonds of both the β-O-4 and α-O-4 linkages had lower BDEs than their C–C counterparts while the C_α_ of the β-5 ring exhibited the lowest BDEs for ring-opening. However, the magnitudes of the bond dissociation enthalpies (BDE) differ based on the surrounding substituents.^34^ Through continuing investigations into the variety of interunit linkages and substructures found in native lignin, it is possible to build a library of potential reactions. Given you have characteristics of the lignin, this library will form a reaction mechanism comparable to the mechanisms proposed for cellulose and hemicellulose.^6,7,11^

In this study, two model oligomers, containing β–β′ and 4-O-5 linkages, are investigated using DFT to determine the BDEs of homolytic cleavage reactions at relevant bonds along the interunit linkages. The β–β′ and 4-O-5 have not received the level of attention as other more prevalent linkages, such as β-O-4; however, there are previous dimer studies for both linkages that serve as good points of comparison.^28,35^ In a DFT study of pinoresinol, a dimer with a β–β′ linkage, the C_α_–O and C_α_–C_β_ bonds exhibited the lowest BDEs, which were supported by visualization of significant delocalization of the unpaired electrons from the resulting product.^28^ Additionally, the 4-O-5 linkage, as part of a large BDE study of multiple linkage types, was found to have high BDEs among the ether linkages even higher than the C_α_–C_β_ bond of the β-O-4 linkage.^35^ The authors hypothesize similar trends will be present in the oligomers where the ring-opening reactions involving the alpha carbon of the β–β′ linkage will have the lowest BDEs of the β–β′ linkage. This is due to the resulting radicals on the alpha carbon should exhibit higher electron delocalization along the neighboring aromatic ring. Additionally, the authors believe both 4-O-5 bonds will have appreciable higher BDE than the β–β′ and other ether linkages as the carbons of this ether linkage are directly part of an aromatic ring. These hypotheses line up with the previously reporting findings for model dimers.^28,35^ Based on previous oligomer works, the authors believe the general trends found in BDE for each linkage will agree with the trends published for their respective dimers, but the differences will be found in the magnitude of the BDEs.

## Materials and methods

### Model lignin oligomers

**Fig. 1:**
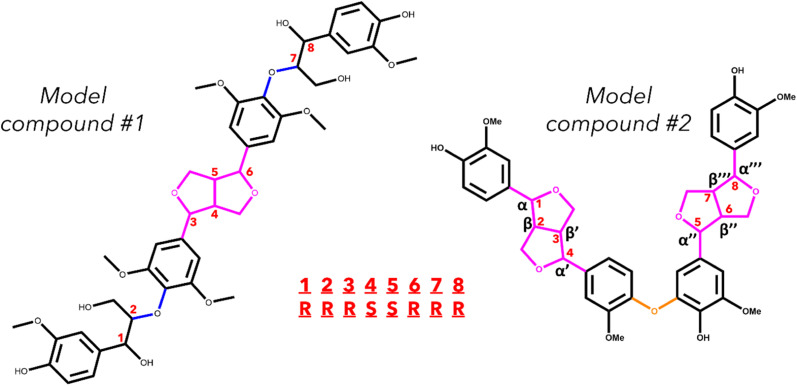
Skeletal structure of the two model lignin oligomers. The interunit linkages are highlighted by color: β-O-4 (blue), β–β′ (pink), and 4-O-5 (orange). The chiral centers of MC1 are indicated with the red numbers. An example of the stereoisomer nomenclature for MC1 is included.

Two model oligomers were chosen for this study, both containing at least one β–β′ and one containing an additional 4-O-5 linkage. The first tetramer, hereafter referred to as model compound 1 (MC1), contains two β–β′ linkages and one 4-O-5 linkage.^36^ The second tetramer, hereafter referred to as model compound 2 (MC2), contains a β–β′ linage and two β-O-4 linkages.^37^ The structures of MC1 and MC2 are shown in Fig. 1. These tetramers were chosen as they contain two important yet understudied interunit linkages found in native lignin. Each relevant bond along the interunit linkages will be investigated; however, due to the well-documented studies on the β-O-4 linkage,^29,34,38–41^ this work will focus primarily on the bond dissociation enthalpies of the β–β′ and 4-O-5 linkages.

MC1, also known as hedyotisol, contains eight chiral centers, which produces 64 possible stereoisomers. However, in the initial identification of hedyotisol, Matsuda *et al.*^37^ proposed a single absolute configuration of the four chiral centers of the β–β′ linkage. Additionally, both β-O-4 linkages were reported as *erythr*o, which further reduces the number of stereoisomers that should be considered. The reported configurations of each linkage reduce the number of possible stereoisomers from 64 to 4. The naming convention for these configurations is also shown in Fig. 1. Each chiral center is labeled by their carbon position (α, β) and presented as their absolute configuration (*R* or *S*). The chiral centers are ordered from left to right and the resulting combination of absolute configurations is how each stereoisomer will be differentiated from each other. An example of the naming convention using MC1 is as follows: if the four chiral centers of both β-O-4 linkages have the *R* configuration, the resulting stereoisomer will be labeled *RRRSSRRR*.

MC2 is a previously identified model lignin oligomer that contains two β–β′ linkages and a 4-O-5 linkage.^36^ Similar to MC1, MC2 has eight chiral centers located around both of the β–β′ linkages. Assuming the same configuration of the β–β′ in MC1, there is only one possible stereoisomer to account for, which is *RSSRRSSR*.

### Computational details

The computational work in this study was done using Spartan'18 (Wavefunction, Inc., Irvine, CA, 2018), GaussView 6, and Gaussian 16 (Gaussian, Inc., Wallingford, CT, 2016). The calculations in this project can be broken down into two categories that are discussed in the following sections: conformational analysis and bond dissociation enthalpy (BDE) calculations. The conformational analysis of each stereoisomer was performed using Spartan 18 on a local desktop using a single processor, while the density functional theory (DFT) calculations were performed using the Gaussian suite of software. The DFT simulations were carried out on the Infrastructure for Scientific Applications and Advanced Computing (ISAAC) high-performance computing resource at the University of Tennessee. Each DFT simulation was performed in parallel using eight processors. Additional discussion on the computational details employed in this work can be found in Houston *et al.*^34^

### Conformational analysis

In both model oligomers, the chiral centers are located at the α and β positions of each respective β–β′ and β-O-4 linkage, due to the free-radical nature of lignin polymerization.^18^ These oligomers can be present in different three-dimensional configurations due to the rotations of individual bonds throughout the molecule. To ensure our calculations are carried out on the lowest energy structure for each stereoisomer of our model oligomers, a Monte Carlo conformational analysis was performed to identify the lowest energy conformer. The initial Monte Carlo search found the 500 lowest energy conformers using the molecular mechanics force field MMFF94.^42^ The remaining conformers were further refined down to the 10 lowest energy conformers by performing a geometry optimization using a semi-empirical PM6 level of theory.^43^ The 10 conformers were then optimized by DFT using the M06-2X a 6-31+G(d) to find the lowest energy conformer, which was further optimized using a more robust 6-311++G(d,p) basis set.^44–48^ The dispersion interactions were described using the GD3 empirical dispersion correction.^49^

### Determination of bond scission energetics

**Fig. 2:**
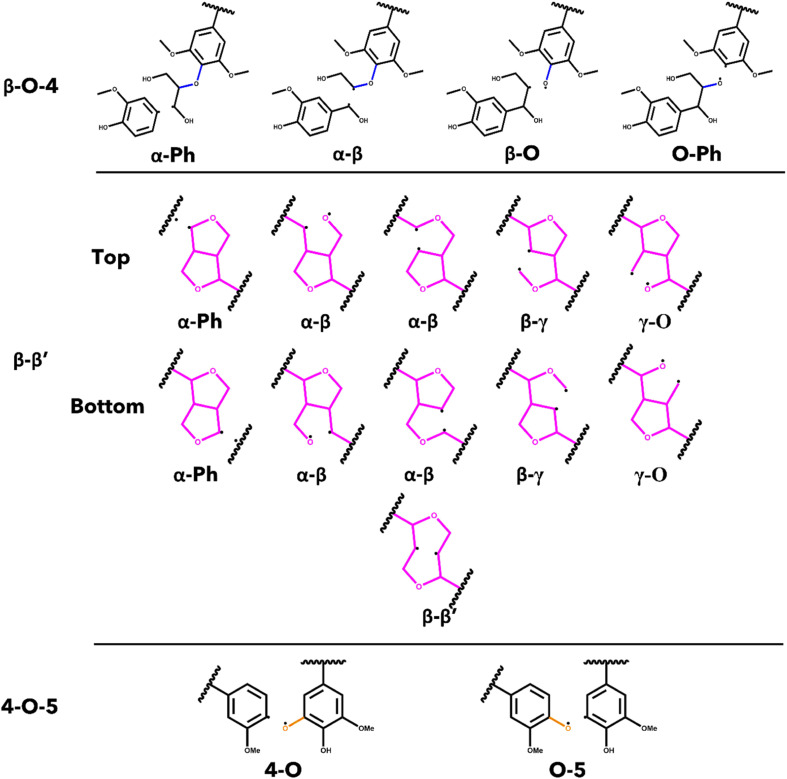
List of homolytic bond scissions for each interunit linkage investigated in this study.

The energetics associated with the homolytic cleavage of relevant bonds along each interunit linkage were determined *via* a two-step calculation procedure consisting of geometry optimization and frequency analysis at 773.15 K (500 °C). This is an established moderate temperature for biomass fast pyrolysis and energetics determined for this temperature will be relevant for comparisons with experimental studies. For each stereoisomer, the starting oligomers as well as the resulting products from each homolytic cleavage were generated from the lowest energy conformer determined by conformational analysis. The products from cleaving the bonds in the β-O-4 and the 4-O-5 linkages are two, separate radical species, while most of the products from scission of bonds in the β–β′ are single, di-radical species. In the case of the di-radical species, special care was taken to ensure they did not immediately re-bond during geometry optimization. The cleavage location for every relevant bond along each interunit linkage is shown in Fig. 2. The di-radical species were treated as triplets and the initial distance between the resulting radicalized atoms was set to 2.5 A to eliminate the possibility of re-bonding. The bond dissociation enthalpies (BDE) were calculated as the difference between the sum of the thermal enthalpies of the products and the reactants.^50^

## Results and discussion

### Conformational analysis

**Fig. 3:**
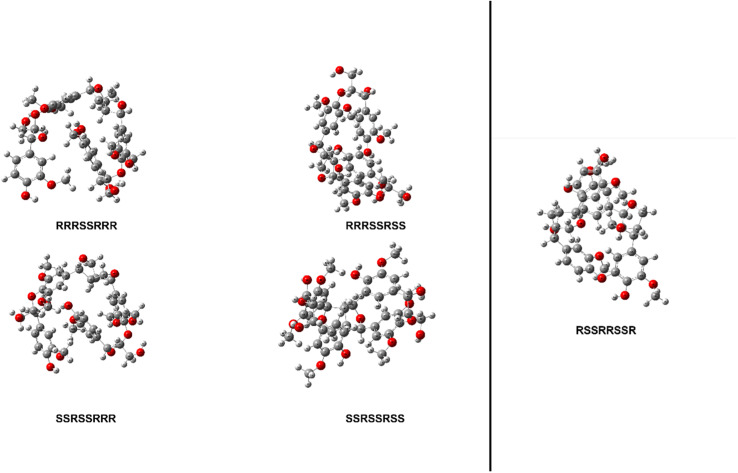
Optimized geometries of each stereoisomer of MC1 (left) and MC2 (right) determined using M06-2X/6-311++G(d,p).

The thermal enthalpies, the sum of the electronic and thermal enthalpies, of each stereoisomer's ten conformers were calculated at 298.15 K using the M06-2X functional with a 6-31+G(d) basis set for both model compounds. The conformers of stereoisomers for MC1 and MC2 were shown to have an appreciable difference in their enthalpies, ranging from 4.52 to 9.14 kcal mol^−1^ and 10.07 kcal mol^−1^, respectively. The lowest energy conformer of each stereoisomer was then optimized using the same Minnesota 06 functional with a 6-311++G(d,p) basis set to determine the initial structure of each stereoisomer for MC1 and MC2. The relative enthalpy differences of stereoisomers for MC1 are shown in Table 1. The range of these enthalpies is 1.55 kcal mol^−1^, which exceeds the accepted ±1.00 kcal mol^−1^ threshold for chemical accuracy so we are unable to say that these stereoisomers have the same total enthalpy.^51^ Therefore, BDEs of relevant linkages will be calculated for all four stereoisomers of MC1. The optimized initial geometries of each stereoisomer for MC1 and MC2 are shown in Fig. 3.

**Table 1:** Relative enthalpy difference between stereoisomers for model compound 1 at the M06-2X/6-311++G(d,p) level of theory at 773.15 K

Configuration	Relative enthalpy difference (kcal mol^−1^)
*RRRSSRRR*	1.55
*RRRSSRSS*	0.43
*SSRSSRRR*	0.75
*SSRSSRSS*	0

### Bond dissociation enthalpies (BDE)

The bond dissociation enthalpies (BDE) were calculated for each linkage along the β–β′ and 4-O-5 linkages. The three-dimensional coordinates for each investigated compound are included in the ESI.† The linkages along the backbone of the β-O-4 linkages were also investigated; however, based on previous work, the branching linkages, such as the C_α_–O and C_γ_–O bonds, were not considered.^34^ The BDEs of each homolytic cleavage were determined at 773.15 K (500 °C) to represent a well-accepted operating temperature for biomass fast pyrolysis. Upon determination of the BDEs for each relevant bond, trends were identified for each interunit linkage. Heat maps of the BDEs for MC1 and MC2 are shown in Tables 2 and 3, respectively.

**Table 2:** Bond dissociation enthalpies, Δ*H*_773_, (kcal mol^−1^) for each stereoisomer of MC1 at 773.15 K

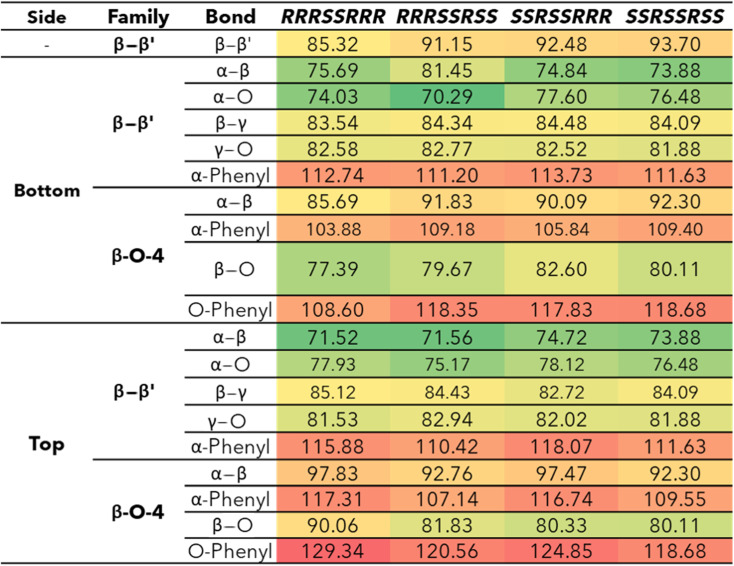

**Table 3:** Bond dissociation enthalpies, Δ*H*_773_, (kcal mol^−1^) of MC2 at 773.15 K

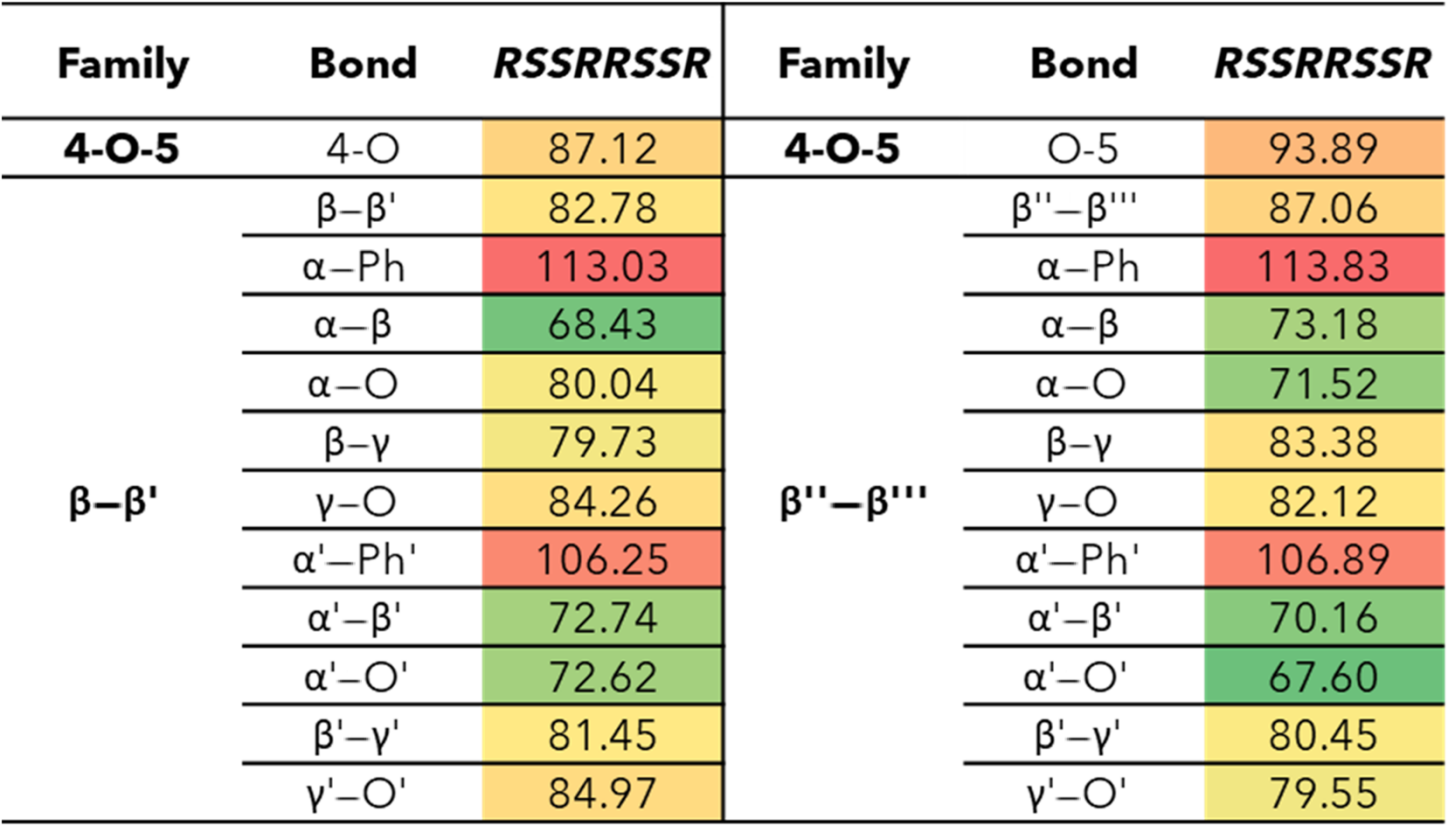

For the β-O-4 linkages of MC1, the C_β_–O bond has a lower BDE than the other bonds along the β-O-4 linkage, ranging from 77.39–90.06 kcal mol^−1^. The non-aromatic component of the ether bond has previously been shown to be the lowest BDE bond in the β-O-4 linkage, therefore, our trends are consistent with previous reports.^35,41,52,53^ The overall trends agree; however, the magnitudes of the BDEs are slightly higher than what has typically been reported for β-O-4 dimers (56–72 kcal mol^−1^).^35,52,54^ Inspection of the three-dimensional geometry shows MC1 is not necessarily linear and can fold in a way that brings the β-O-4 into closer proximity to other aromatics and linkages, which could serve to stabilize the bond. Further investigation into the cause of the magnitude discrepancy is needed; however, it is outside the scope of this study.

**Fig. 4:**
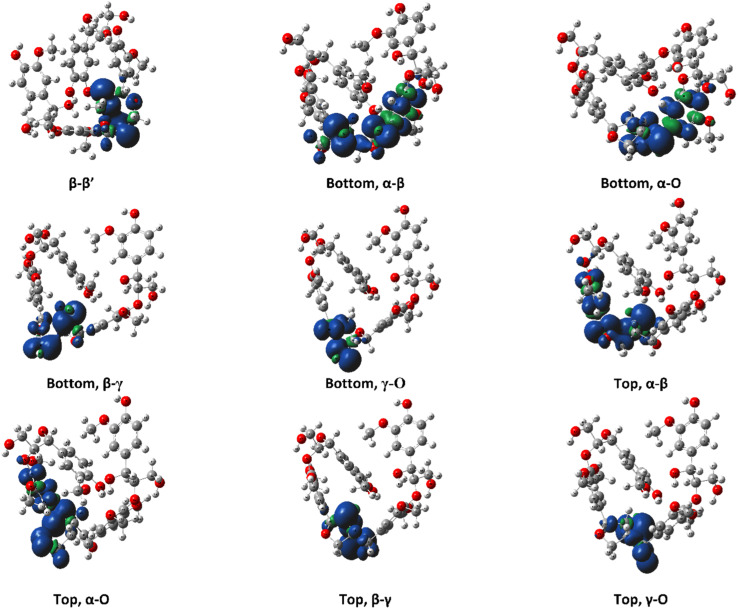
Spin density plots for the di-radical species formed from scission along the β–β′ linkage for the *RRRSSRRR* stereoisomer in MC1.

The ring-opening scissions in the β–β′ linkage, for both MC1 and MC2, were shown to have the BDEs in the range of 70.29–87.06 kcal mol^−1^. The reactions involving each C_α_ (excluding bonds to an aromatic ring) exhibited lower BDEs then the other β–β′ ring-opening reactions. The C_α_–O bond had a range of 70.29–80.04 kcal mol^−1^, while the C_α_–C_β_ had a range of 68.43–81.45 kcal mol^−1^. This trend agrees with previous BDEs published for a pinoresinol dimer.^28^ The magnitudes of the BDEs for these reactions are slightly larger than previously reported dimer values but have much closer agreement than the BDEs for the β-O-4 linkage. Spin density plots for each di-radical produced from the β–β′ ring opening scission of MC1 are shown in Fig. 4. The spin density backs up what is seen from our BDE calculations. For the C_α_–O and C_α_–C_β_ scissions, there is significantly more delocalization of the unpaired electrons, which leads to more stable, di-radical products. The more stable the resulting product, the lower the BDE for the scission of that bond will be. The di-radical species produced from the scission of other bonds along the β–β′ linkage do not possess similar levels of electron delocalization. Instead, the spin density is centered around the remaining β–β′ ring. This same trend and spin density phenomenon holds true for the linkages of MC2 as well.

The 4-O-5 linkage found in MC2 has similar BDEs for both the 4-O and the O-5 bond. The 4-O bond (87.12 kcal mol^−1^) was shown to have a slightly lower BDE than the O-5 bond (93.89 kcal mol^−1^). The 4-O-5 linkage is a strong ether linkage due to the proximity of the aromatic rings. The cleavage of these bonds is not favored compared to other ether linkages, such as the β-O-4, due to the fact scission would result in a radical on an aromatic carbon, which would disrupt the aromaticity. Additionally, the spin density is concentrated around the radical making the product unstable. Therefore, we see the 4-O-5 linkage has higher BDEs than other ether linkages.

The results obtained from BDE calculations in this study require comparison with experimentally observed products for validation. Currently, there has been no experimental investigation into these two model compounds; however, it is possible to use previous experimental work involving the linkages of interest for comparison. Formation of phenolic monomer compounds *via* the β–β′ has been proposed to initiate *via* the homolytic cleavage of the C_α_–O bond.^55^ Additionally, experimental investigation of pinoresinol, a dimer containing a β–β′ linkage, also proposed an initial cleavage of the C_α_–O with the C_α_–C_β_ being a secondary point of reaction.^56^ Comparison with our BDEs from DFT, we would expect the C_α_–O to be the primary point of reaction followed by the C_α_–C_β_. For the 4-O-5 linkage, diphenyl ether has been experimentally investigated with the primary point of reaction being homolytic cleavage of the C–O bond leading to either stabilization or recombination.^57^ The subsequent reaction pathways of the two-linkage proposed by these previous studies will be further investigated in future DFT work.

This work builds off previous DFT work on model lignin oligomers in an attempt to gain a better understanding of how different interunit linkages behave in larger, more representative lignin substructures.^34^ The current work combined with the author's previous study has now investigated the pyrolysis behavior of five interunit linkages in oligomer sized model compounds. Additional investigation is still needed to incorporate more linkages as well as different combinations of linkages in each oligomer. The fast pyrolysis scientific community is gradually gaining a better mechanistic understanding of the pyrolytic deconstruction behavior of lignin. Investigations of larger model structures is another necessary step forward to bridge the current knowledge gap from monomers and dimers to native lignin.

## Conclusions

The use of larger model compounds, such as oligomers, attempts to bridge the knowledge gap between model monomers and dimers and native lignin. In this work, the authors computationally determined, with density functional theory, the bond dissociation enthalpies of two model lignin oligomers containing two underrepresented interunit linkages, β–β′ and 4-O-5. Understanding the bond dissociation energies (BDEs) of relevant bonds provides information about the most likely points of reactions during the thermal deconstruction of these oligomers during biomass fast pyrolysis. Conformational analysis of every relevant stereoisomer for both model compounds showed a relative enthalpy difference of 1.55 kcal mol^−1^, which is over the threshold of chemical accuracy. Therefore, multiple stereoisomers were simulated for the first model oligomer. Regardless of configuration, the bonds containing alpha carbons in the β–β′ linkage had the lowest BDEs (C_α_–O and C_α_–C_β_) with ranges of 70.29–80.04 kcal mol^−1^ and 68.43–81.45 kcal mol^−1^, respectively. The 4-O-5 bonds of MC2 had much higher BDEs than other ether linkages such as the β-O-4 linkage in MC1 and other ether linkages in literature. Spin density plots showed excessive delocalization of radicals around the alpha carbons, which helps explain the lower BDEs of the respective bonds. Even though the trends were the same as previous dimer work, the magnitudes of the BDEs for the model compounds in this work were typically larger than what was reported for their dimer counterparts. Further investigation is needed to determine the exact cause of the discrepancies in the magnitudes of the BDEs. This study builds on a previous model oligomer study and provides another step towards developing a library of interunit linkage behavior that can potentially be used for generalized reaction rules for the pyrolysis of lignin.

## Conflicts of interest

The authors declare no competing financial interest.

## Supplementary Material

RA-013-D2RA07787F-s001
