# Supplementary material for: Investigation of the thermal deconstruction of β–β′ and 4-O-5 linkages in lignin model oligomers by density functional theory (DFT)
Source: RSC Adv. 2023 Feb 21;13(9):6181–90. doi: 10.1039/d2ra07787f (PMC9941757; doi:10.1039/d2ra07787f)
Supplement: RA-013-D2RA07787F-s001 [file RA-013-D2RA07787F-s001.pdf]

Electronic Supplementary Material (ESI) for RSC Advances.
This journal is © The Royal Society of Chemistry 2023

Investigation of the thermal deconstruction of β-β' and 4-O-5 linkages in lignin model oligomers by density functional theory (DFT)

Ross W. Houston[a] and Nourredine H. Abdoulmoumine[a,b]*

[a.]Department of Biosystems Engineering and Soil Science, University of Tennessee, 2506 E. J. Chapman Drive, Knoxville, TN 37996, USA

[b.]Center for Renewable Carbon, University of Tennessee, 2506 Jacob Drive, Knoxville, TN 37996. USA

Supporting Information

S.1.Optimized geometries of the initial stereoisomers of MC1

RRRSSRRR

O 1.81714707 4.75311763 -0.83164502
C 2.86768026 4.16621231 -0.19099402
C 5.13923862 3.23738791 1.09943302
C 3.96153308 5.02610689 0.01132056
C 2.91855949 2.85208212 0.24506845
C 4.06888201 2.38097281 0.88932897
C 5.08959316 4.55990981 0.65900090
H 6.02742481 2.87639841 1.60675288
H 2.07646289 2.18227214 0.10996788
H 5.91576147 5.24183334 0.81710906
C 0.63722544 3.98078479 -0.96768191
H-0.08829029 4.60638873 -1.48416352
H 0.25364721 3.69799059 0.01636398
H 0.82160484 3.07640180 -1.55421387
O 3.90027738 6.30961942 -0.41958165
H 3.03773421 6.44318545 -0.83112714
C 4.14693337 0.92823331 1.28373870
H 4.98542268 0.79290542 1.98065069
C 4.42001620 0.04009411 0.06004220
H 3.57654267 0.10209820 -0.63756878
C 5.72711598 0.39066358 -0.63404214
H 5.70262269 1.43698305 -0.93965458
H 6.54186447 0.26538051 0.09460078
O 5.96391264 -0.38001978 -1.78668221
H 5.59540852 -1.25815709 -1.63809541
O 4.55735491 -1.33404827 0.51272878
C 3.48449406 -2.16729491 0.38645025
C 1.29479930 -3.90339947 0.15959529
C 3.08588004 -2.65096183 -0.86945143
C 2.81060435 -2.62220479 1.51777429
C 1.72631029 -3.48286759 1.41180416
C 1.99147705 -3.50770413 -0.98074654
H 1.21917004 -3.78531942 2.32183330
H 1.67411165 -3.88081057 -1.94497233
O 3.82523826 -2.21554997 -1.91389587
O 3.16772907 -2.14410187 2.75730034
C 3.37017004 -2.49351197 -3.23186913
H 4.06042101 -1.97335857 -3.89201140
H 2.35749767 -2.10815750 -3.37363428
H 3.40226547 -3.56739541 -3.43345011
C 4.39949749 -2.67610592 3.25856467
H 4.56169051 -2.21137987 4.22908269
H 4.31082900 -3.75880643 3.37654641

H 5.22078775 -2.43524154 2.58187862
C 0.04893148 -4.76577052 0.04068534
C-1.17871821 -4.04640922 0.60365263
C-1.70054484 -3.23912436 -0.59787008
C-1.11295668 -3.98647937 -1.81096645
O-0.29363704 -5.04545089 -1.30339752
H 0.22233563 -5.72978705 0.52932576
H-0.94963918 -3.40867175 1.45812101
H-1.35054642 -2.20824190 -0.57894522
H-0.51257103 -3.30815150 -2.42443400
H-1.87821009 -4.44738017 -2.43896502
C-3.24538100 -3.28807396 -0.43203379
O-3.50436982 -4.12850352 0.68501508
C-2.37216267 -4.95216975 0.89825439
H-3.70740560 -3.72486254 -1.32657650
H-2.37707693 -5.80711784 0.20811329
H-2.41234674 -5.31874915 1.92417853
C-3.81295239 -1.90950071 -0.19692880
C-4.54846550 0.73991471 0.18426744
C-4.07991282 -1.10515663 -1.30121889
C-3.94845932 -1.41976932 1.09680187
C-4.32916323 -0.09076322 1.28422710
C-4.45072621 0.22373045 -1.10863943
H-3.97229825 -1.51379302 -2.29797790
H-3.76881595 -2.08467486 1.92980266
O-4.71463624 1.10406439 -2.10914541
O-4.48732098 0.50520499 2.49376968
C-4.32587071 -0.29682126 3.64826536
H-4.52447298 0.35637726 4.49462685
H-3.30563043 -0.68668142 3.71829339
H-5.03430207 -1.12973548 3.64830699
C-4.56867903 0.64269092 -3.44046166
H-4.78843448 1.49550194 -4.07941202
H-5.27698419 -0.16335199 -3.65096538
H-3.54598235 0.29969657 -3.62180851
O-4.87582478 2.05727694 0.35797114
O 2.93044791 0.55886419 1.90456866
H 3.05094250 -0.30197112 2.32948677
C-3.80973220 2.91068151 0.81474949
H-3.27226744 2.40491151 1.62050779
C-4.51563458 4.11995954 1.39959190
H-3.77585034 4.84361387 1.74820848
H-5.12447022 4.59036046 0.62006577
O-5.29482955 3.74918030 2.51564959
H-5.77938107 2.95414060 2.26639622
C-2.87331588 3.27262682 -0.34714223
H-2.24408767 4.10065188 0.00383891
O-3.62049821 3.78956968 -1.42299373
H-4.27085091 3.11012584 -1.65680943
C-1.94976483 2.13373121 -0.78548754
C-0.22684968 0.08637128 -1.62060595
C-1.29700627 1.32809834 0.15969933
C-1.70978111 1.91184775 -2.13417224
C-0.85310930 0.89059749 -2.55309495
C-0.45121863 0.30968128 -0.25016362
H-1.45510250 1.48800421 1.21846401
H-2.19544717 2.54439516 -2.86646000
H-0.65814096 0.71493844 -3.60472356
O 0.20649840 -0.56044083 0.56477797
O 0.59786986 -0.92166336 -2.00886267
H 0.94219893 -1.35855026 -1.21403367
C 0.04649179 -0.38709149 1.96271707
H 0.64926765 -1.16008192 2.43492899
H 0.41676449 0.59305075 2.27183190

H-1.00721528 -0.51132135 2.23732622

RRRSSRSS

O-0.10291281 -2.86870208 0.12065349

C-1.29568747 -2.40743016 -0.36426268

C-3.56794313 -1.38537694 -1.57601710

C-1.17217295 -1.52460969 -1.44404166

C-2.54958524 -2.74168144 0.12487766

C-3.70214410 -2.22237786 -0.47267884

C-2.30991984 -1.02369305 -2.04907380

H-4.43835782 -0.97110202 -2.06938690

H-2.65936859 -3.40208563 0.97505549

H-2.19873986 -0.34874834 -2.88979883

C-0.13467659 -3.71758015 1.25322982

H-0.67389608 -4.64203264 1.03009812

H 0.90260698 -3.94265622 1.49246611

H-0.60242949 -3.21500861 2.10484728

O 0.05580544 -1.16819636 -1.91432718

H 0.73542085 -1.48065908 -1.30174109

C-5.06417601 -2.60717885 0.10972085

H-5.46133517 -3.44610237 -0.47340703

C-6.10328871 -1.48118592 0.04478244

H-6.14043675 -1.02466484 -0.94618361

C-7.50046937 -1.95252235 0.40360421

H-7.80340922 -2.75111537 -0.27678701

H-7.49413126 -2.34699250 1.42529100

O-8.43318142 -0.90387663 0.25752888

H-8.04455571 -0.12628219 0.67359677

O-5.80894763 -0.46465645 1.02169998

C-4.79316777 0.40468098 0.72453356

C-2.70124215 2.14945295 0.14845231

C-4.94409615 1.37844682 -0.26325631

C-3.59523774 0.32301523 1.43575794

C-2.54573237 1.19314833 1.15101416

C-3.89454590 2.24821964 -0.55918243

H-1.62528877 1.15032817 1.71876756

H-3.99081119 3.01863883 -1.31183561

O-6.14740728 1.37582105 -0.89394560

O-3.54008871 -0.65824764 2.37781455

C-6.36444383 2.35021167 -1.89661148

H-7.37548898 2.18308344 -2.26051930

H-5.65277859 2.23252395 -2.71950748

H-6.28224429 3.36052183 -1.48636205

C-2.31159414 -0.82965754 3.06120281

H-2.45276034 -1.68116991 3.72461988

H-1.50131076 -1.03764809 2.35549250

H-2.05749863 0.05765201 3.64654931

C-1.59803076 3.16957031 -0.09299297

C-0.19287713 2.56866338 -0.21288964

C-0.03624317 2.30063578 -1.72813703

C-1.18372781 3.12113139 -2.35012604

O-1.77124284 3.88590200 -1.30260650

H-1.64460438 3.90939598 0.71177345

H-0.06770663 1.66826427 0.38016186

H-0.12375645 1.24179111 -1.96810442

H-1.93551962 2.45246975 -2.78671088

H-0.84486921 3.82527583 -3.11158192

C 1.36920290 2.84787961 -2.03287345

O 1.51838315 3.94387851 -1.15018547

C 0.91828017 3.58985222 0.09272075

H 1.43836940 3.24833750 -3.04841329

H 0.53791621 4.51088280 0.53679789

H 1.66432493 3.14781051 0.76457796

C 2.46041786 1.80397911 -1.83126147

C 4.40054008 -0.14037123 -1.38747490

C 2.41101033 0.60530511 -2.54013424

C 3.50515813 2.05985131 -0.95008862

C 4.47237533 1.08281341 -0.72554456

C 3.38857223 -0.36319743 -2.32542048

H 1.60078912 0.41709645 -3.23199102

H 3.54711837 3.01998378 -0.45717876

O 3.43312167 -1.57285566 -2.94258419

O 5.51431497 1.22120876 0.14148543

C 5.58014806 2.42398697 0.88382444

H 6.43419590 2.31902539 1.55112795

H 5.73610336 3.28226838 0.22468212

H 4.66534688 2.57173114 1.46779859

C 2.57535997 -1.77838169 -4.05610688

H 2.83591884 -2.75785627 -4.45056320

H 1.52580614 -1.76861611 -3.75408149

H 2.75079289 -1.01430636 -4.81897250

O 5.34241242 -1.10215589 -1.13630078

O-4.95198192 -3.08141758 1.43387217

H-4.72161510 -2.31397221 1.97968062

C 4.89460223 -2.24267469 -0.38142004

H 3.90952630 -2.54554928 -0.74479500

C 5.88701180 -3.33832329 -0.72326466

H 5.64731901 -4.24088391 -0.15682263

H 6.89359742 -3.00773201 -0.44444599

O 5.80926609 -3.67021419 -2.09179690

H 5.74229812 -2.84048203 -2.57837808

C 4.86472248 -1.90967186 1.11748922

H 4.78688043 -2.86057988 1.65733254

O 6.09610291 -1.34693635 1.50781913

H 6.24725742 -0.58816798 0.92374921

C 3.67735041 -1.03628914 1.53107071

C 1.53788198 0.57768518 2.34632611

C 3.87986192 -0.01508512 2.46342821

C 2.39425863 -1.24782480 1.03665307

C 1.32833380 -0.44089085 1.43363445

C 2.82282082 0.78812419 2.86241848

H 4.87605375 0.13624289 2.85778648

H 2.19706921 -2.05213309 0.33767204

H 0.32958479 -0.60098921 1.04001228

O 2.90297373 1.82164355 3.75553036

O 0.50016539 1.35990382 2.75948932

H 0.84319803 1.99025644 3.40594989

C 4.09752047 1.94246884 4.50854079

H 4.94352914 2.19777378 3.86492607

H 4.31291007 1.01196729 5.04052766

H 3.92815024 2.74671349 5.22051747

SSRSSRRR

O-1.29097099 -4.42193784 1.11766354

C-2.31623324 -3.96672278 0.34474984

C-4.42257771 -3.30851227 -1.33137756

C-2.90251294 -4.95559631 -0.46440718

C-2.77894233 -2.66188803 0.30072222

C-3.84872168 -2.32255615 -0.53974890

C-3.95107336 -4.62090007 -1.30100220

H-5.25121095 -3.05480846 -1.98308417

H-2.29737692 -1.90910302 0.91157653

H-4.39383485 -5.39668463 -1.91336210

C-0.76927057 -3.53866353 2.09913127

H-1.55815242 -3.24023025 2.79658900

H-0.32432306 -2.65660648 1.63533329

H-0.00005697 -4.09732184 2.62781269

O	-2.43255190	-6.22516152	-0.42699171
H	-1.71594365	-6.25819355	0.21914405
C	-4.36945221	-0.90486727	-0.61434672
H	-5.38860981	-0.93209520	-1.02083290
C	-4.42814133	-0.22564709	0.75834204
H	-3.45384483	-0.27546786	1.24871042
C	-5.49563152	-0.84144750	1.64574304
H	-5.38004354	-1.92705576	1.65915276
H	-6.47513252	-0.60275135	1.20910445
O	-5.41395782	-0.39213320	2.97820618
H	-5.16586945	0.53953377	2.95293848
O	-4.78073350	1.16616079	0.59711668
C	-3.74268346	2.05880745	0.57920163
C	-1.57502718	3.81329407	0.50055875
C	-2.96067049	2.27429411	1.72042433
C	-3.47034336	2.79544199	-0.57146359
C	-2.39439483	3.68131695	-0.60982087
C	-1.86649762	3.12891613	1.67831666
H	-2.18110300	4.26627454	-1.49195047
H	-1.23905603	3.26639671	2.55089972
O	-3.35015999	1.57662149	2.81951726
O	-4.28252543	2.54291948	-1.64129568
C	-2.48890158	1.55644248	3.94205271
H	-1.49748835	1.18911542	3.66054828
H	-2.40279631	2.55030354	4.39002266
H	-2.94530877	0.87165069	4.65368278
C	-4.07441891	3.30526562	-2.81891943
H	-3.08309898	3.11380185	-3.24002426
H	-4.18970692	4.37225493	-2.61397701
H	-4.83926931	2.98316985	-3.52165924
C	-0.32247777	4.66679669	0.43207144
C	0.94662050	3.80349821	0.43346851
C	1.14031523	3.47365689	-1.05674731
C	0.46985831	4.66987570	-1.75853321
O	-0.23801384	5.41524462	-0.76694621
H	-0.32542466	5.38510279	1.25761112
H	0.83490865	2.90496824	1.03823665
H	0.68025141	2.52250738	-1.32625473
H	-0.22112062	4.33758022	-2.53948139
H	1.20773296	5.34168957	-2.20329047
C	2.66448136	3.47759702	-1.21867125
O	3.10611101	4.49547138	-0.33789567
C	2.22827048	4.58156125	0.78441230
H	2.95693177	3.78906150	-2.22637404
H	2.02870532	5.64117410	0.95990931
H	2.71581157	4.16094642	1.66989845
C	3.30161936	2.12997273	-0.93018197
C	4.47397660	-0.34685944	-0.46366294
C	4.18351566	1.98024408	0.13199129
C	3.02094336	1.06113980	-1.78117326
C	3.62360471	-0.17345077	-1.55905086
C	4.77086309	0.73478902	0.36277837
H	4.42868620	2.84105301	0.73763881
H	2.34450872	1.20398111	-2.61465327
O	5.63955122	0.46271371	1.37153223
O	3.44371718	-1.27358019	-2.33488955
C	2.55395614	-1.16045888	-3.43070655
H	2.52579893	-2.14434745	-3.89562834
H	2.92204068	-0.42759612	-4.15451722
H	1.55007044	-0.88119368	-3.09537488
C	5.98029988	1.51813353	2.25004118
H	6.47085777	2.33399930	1.71194787
H	6.66845864	1.09085591	2.97566031
H	5.09392707	1.90097265	2.76508610

O 5.03541921 -1.57293628 -0.22988866

O-3.54408588 -0.16618006 -1.51195307

H-3.98153131 0.68178700 -1.68578029

C 4.52624309 -2.29775864 0.90776286

H 4.36502720 -1.59923189 1.73184092

C 5.64988917 -3.24000302 1.29492220

H 5.32604180 -3.87804404 2.12012561

H 5.89477049 -3.87593460 0.43745012

O 6.77502028 -2.51532417 1.74297377

H 6.88757390 -1.77065065 1.14072990

C 3.23047441 -3.02575339 0.52709783

H 3.01366421 -3.74681239 1.32404855

O 3.44498283 -3.79414666 -0.63582057

H 3.76615847 -3.17890749 -1.31196162

C 2.03338363 -2.08478626 0.37793763

C-0.13838705 -0.34487124 -0.01591123

C 1.13987449 -2.29357422 -0.67781803

C 1.80609925 -1.02098139 1.24624048

C 0.72656452 -0.15963674 1.05179793

C 0.07866612 -1.42575871 -0.88743165

H 1.30959480 -3.12504977 -1.34974192

H 2.46941437 -0.83498010 2.08259911

H 0.53699786 0.66694214 1.72736698

O-0.80927254 -1.49819505 -1.91409285

O-1.17853711 0.49494121 -0.19972551

H-1.81257065 0.13692625 -0.85522336

C-0.90497041 -2.71795987 -2.62699173

H-0.98131951 -3.56394316 -1.93945575

H-1.81451790 -2.64727583 -3.22005948

H-0.04030309 -2.85792585 -3.28384777

SSRSSRSS

O-3.85358031 3.85960224 0.68167508

C-3.39017848 2.82309933 -0.08366332

C-2.21000506 0.76417547 -1.51032869

C-2.02527369 2.55914953 0.07967198

C-4.15349261 2.05868930 -0.94907504

C-3.57262247 1.00456763 -1.65933056

C-1.43583855 1.53463602 -0.64260822

H-1.72551850 -0.03708834 -2.05627039

H-5.21113538 2.24700629 -1.07591566

H-0.37490667 1.34014981 -0.51597546

C-5.16468957 4.32820561 0.41848558

H-5.30984114 5.19901398 1.05339284

H-5.26896439 4.61102972 -0.63238554

H-5.90963164 3.56729348 0.66632069

O-1.29230227 3.32307929 0.94055364

H-1.88387770 3.98565440 1.32015219

C-4.46672438 0.16946740 -2.57916930

H-4.30609408 0.50364194 -3.61032319

C-4.16401862 -1.33301412 -2.52917501

H-3.09070867 -1.52723259 -2.58859898

C-4.85250576 -2.11688214 -3.63052563

H-4.56624455 -1.71151474 -4.60359345

H-5.93727962 -2.01589735 -3.51556445

O-4.45176138 -3.46929106 -3.60475089

H-4.41187441 -3.73438060 -2.67823696

O-4.67839766 -1.89223606 -1.30509860

C-3.87175400 -1.76471161 -0.20556442

C-2.20960332 -1.46057016 2.00085200

C-4.22906322 -0.89594142 0.82237488

C-2.70627593 -2.52837635 -0.09803773

C-1.86398616 -2.36686639 0.99730092

C-3.39540937 -0.74022670 1.93217227

H-0.94742039 -2.93753165 1.07901038
H-3.65417052 -0.08045051 2.74809144
O-5.40510622 -0.23386110 0.64431025
O-2.48562639 -3.38180400 -1.13062307
C-5.78238150 0.69372912 1.64322843
H-5.02553712 1.47751893 1.75624257
H-6.72128969 1.13087025 1.30703421
H-5.93816959 0.19347340 2.60302654
C-1.33604377 -4.20992000 -1.06626766
H-1.36492064 -4.82481114 -1.96286498
H-1.36466687 -4.84593013 -0.17703191
H-0.41915895 -3.61541113 -1.05387742
C-1.26382317 -1.28769529 3.17932222
C 0.10321162 -0.76126098 2.73433990
C-0.10320678 0.76143481 2.73430863
C-1.23163060 0.96319193 3.75985490
O-1.72366251 -0.33047782 4.11728321
H-1.18114078 -2.23962920 3.71244577
H 0.39385148 -1.13889840 1.75851617
H-0.39383953 1.13903371 1.75846706
H-2.04055145 1.56455745 3.33015362
H-0.87856406 1.44086370 4.67666583
C 1.26383193 1.28788264 3.17927068
O 1.72365603 0.33070916 4.11728283
C 1.23163966 -0.96297771 3.75989200
H 1.18116355 2.23984661 3.71234225
H 0.87858750 -1.44062745 4.67671932
H 2.04056231 -1.56434583 3.33019877
C 2.20961445 1.46069128 2.00079338
C 3.87175859 1.76470522 -0.20564681
C 3.39538606 0.74029048 1.93211572
C 1.86403438 2.36699006 0.99723290
C 2.70632230 2.52843457 -0.09811889
C 4.22903871 0.89594544 0.82231185
H 3.65412803 0.08051668 2.74804356
H 0.94750078 2.93770671 1.07894340
O 5.40504568 0.23380245 0.64425559
O 2.48571302 3.38187116 -1.13070518
C 1.33618866 4.21006804 -1.06633726
H 0.41926294 3.61562456 -1.05390697
H 1.36487882 4.84609543 -0.17711544
H 1.36508542 4.82493672 -1.96294907
C 5.78230414 -0.69373751 1.64322799
H 5.02543128 -1.47749068 1.75630533
H 5.93812699 -0.19342387 2.60298980
H 6.72118904 -1.13093628 1.30704416
O 4.67839316 1.89216211 -1.30519887
O-5.83516857 0.38361984 -2.31284428
H-6.01803138 -0.05477080 -1.46798751
C 4.16400043 1.33286301 -2.52923651
H 3.09069003 1.52707688 -2.58866346
C 4.85247238 2.11666986 -3.63064286
H 4.56620147 1.71124422 -4.60368322
H 5.93724813 2.01569798 -3.51568661
O 4.45172070 3.46907842 -3.60494427
H 4.41183690 3.73422069 -2.67844524
C 4.46671560 -0.16961943 -2.57915278
H 4.30610736 -0.50384196 -3.61029458
O 5.83516101 -0.38374680 -2.31280230
H 6.01800677 0.05466958 -1.46795532
C 3.57260930 -1.00468201 -1.65928362
C 2.02527194 -2.55916346 0.07982104
C 4.15347921 -2.05877076 -0.94898127
C 2.20999117 -0.76428094 -1.51028566

C 1.43583245 -1.53469063 -0.64251568
C 3.39017097 -2.82313079 -0.08351768
H 5.21111993 -2.24710031 -1.07581886
H 1.72550104 0.03695008 -2.05627169
H 0.37490032 -1.34019906 -0.51588680
O 3.85357954 -3.85959362 0.68187169
O 1.29230837 -3.32303088 0.94076347
H 1.88388172 -3.98559188 1.32038965
C 5.16467314 -4.32823559 0.41867097
H 5.90963683 -3.56732820 0.66645791
H 5.30982204 -5.19901979 1.05361198
H 5.26891881 -4.61110470 -0.63219072

S.2.Optimized geometries of the initial stereoisomers of MC2

RSSRRSSR

C 1.88429498 4.02396804 -1.21780430
C 2.29284171 2.13306786 0.78668251
C 0.84277555 3.70341490 -0.34742435
C 3.09492240 3.34860292 -1.13857644
C 3.29194733 2.38251461 -0.16221359
C 1.06674181 2.77771029 0.66313307
H 3.89623043 3.53582834 -1.84309569
H 0.28361238 2.54177330 1.37311489
O 4.48644479 1.68258021 -0.16205294
C 4.41533988 0.30726543 -0.30634060
C 4.43990135 -2.44951590 -0.33730811
C 5.59423216 -0.36079738 0.01843531
C 3.27133813 -0.37793636 -0.67860661
C 3.26555323 -1.76774357 -0.64618559
C 5.60673445 -1.75797071 -0.02788811
H 4.47388999 -3.53362110 -0.30528913
H 2.38059426 0.16621997 -0.95868254
C 1.94805197 -2.51281362 -0.79394358
O 0.90969861 -1.67036993 -1.26300094
C 0.29002840 -1.01116113 -0.14513584
C 0.79716530 -1.70085361 1.13226049
C 1.45985497 -2.97410579 0.58872087
H 2.04881096 -3.33923487 -1.50402154
H 0.51136193 0.05868266 -0.16360571
H -0.78749510 -1.13812875 -0.25215532
C 0.28722950 -3.98107137 0.57940483
O -0.83411374 -3.34436289 1.19085416
C -0.36469087 -2.24255023 1.95956299
H 0.54462777 -4.88693936 1.13750784
H -0.00995044 -4.26437688 -0.43317919
H 0.00725556 -2.60811818 2.92878407
H 1.48139959 -1.06740698 1.69487566
H 2.28325479 -3.32265501 1.21149305
H 1.73843216 4.77530320 -1.98727015
C -0.52099803 4.32968083 -0.53367662
O -1.31191068 4.08477017 0.62367071
C -2.64596040 3.78170976 0.24319767
C -2.50290741 3.04729079 -1.07860706
C -1.33813558 3.78545790 -1.75769005
H -0.39300919 5.41232108 -0.65430440
H -3.09857261 3.18710708 1.03937277
H -3.22656509 4.70394803 0.11288469
H -3.41459190 3.06634888 -1.67719978
H -1.68092252 4.61388649 -2.37892385

C -0.71156288 2.67277963 -2.58483029

O -0.78326759 1.52533720 -1.75743032

C -1.95346931 1.60671027 -0.94049045

H 0.33323181 2.82456904 -2.85324826

H -1.29794455 2.52577650 -3.50162993

H -1.61641651 1.44183303 0.09127718

C -2.94889308 0.52107644 -1.28553097

C -4.70256876 -1.60963946 -1.69691972

C -4.25783293 0.58599931 -0.82385508

C -2.51877682 -0.61115388 -1.98295139

C -3.38417168 -1.67651443 -2.17057458

C -5.13758383 -0.47316780 -1.03971254

H -4.59910625 1.45314497 -0.26664297

H -1.49840425 -0.64724729 -2.34218002

H -6.15810253 -0.44075592 -0.67726352

C -1.47007095 -1.23642609 2.17883736

C -3.47543065 0.67414595 2.49685651

C -2.80803747 -1.59888684 1.99835466

C -1.14958338 0.06971793 2.53310347

C -2.15213589 1.02713456 2.68981992

C -3.80507682 -0.65088846 2.17139943

H -3.04146592 -2.61352338 1.70517730

H -0.11158487 0.36108810 2.66216826

H -1.91542121 2.05432844 2.94565553

O 6.72720832 -2.45686878 0.31765854

O 6.69953600 0.32316026 0.41898818

O 2.42077561 1.21923572 1.79000584

O -3.06718131 -2.84938915 -2.79120527

O -5.54200956 -2.66766511 -1.86595686

O -5.14424118 -0.87355208 2.04764214

O -4.45922642 1.60904603 2.60787849

C -5.56200580 -2.19756285 1.75779480

H -5.24677564 -2.87943170 2.55272215

H -5.16325824 -2.53224440 0.79722568

H -6.64823508 -2.16810964 1.70748176

C -1.70802732 -3.03391377 -3.15663370

H -1.04805551 -2.89351087 -2.29553213

H -1.63219021 -4.05204607 -3.53207608

H -1.42069766 -2.33050133 -3.94311451

C 7.83246745 -2.28314001 -0.56484115

H 7.55990799 -2.60178027 -1.57557624

H 8.62898833 -2.91947102 -0.18404165

H 8.16496635 -1.24416883 -0.57825925

C 3.60914772 1.26583265 2.58064896

H 3.31787508 0.98012576 3.59064243

H 4.02288983 2.27653740 2.59309672

H 4.35439561 0.56127131 2.20633907

H 6.48770535 1.26439332 0.39692251

H -5.30403945 1.15876208 2.48261988

H -5.05621909 -3.35295111 -2.34131621

S.3.Optimized geometries of the radical species produced from the RRRSSRRR stereoisomer of MC1

β-β'Cleavage

O 1.49946136 4.90709098 -0.86300285

C 2.56091653 4.36733216 -0.19926890

C 4.84237974 3.54129002 1.14238293

C 3.61581228 5.27175007 0.01498296

C 2.65574601 3.05990445 0.24955906
C 3.81139772 2.64086235 0.91967062
C 4.74907648 4.85664444 0.68792042
H 5.73352315 3.22100237 1.67144723
H 1.84306309 2.35589828 0.10725287
H 5.54421867 5.57241281 0.85525471
C 0.35300084 4.08759269 -1.01181581
H -0.38363960 4.67734983 -1.55394145
H -0.04160104 3.80484810 -0.03206466
H 0.58506770 3.18270054 -1.57988538
O 3.51122678 6.54825783 -0.42874829
H 2.65166319 6.64283651 -0.85703832
C 3.93314760 1.19768790 1.33989740
H 4.75645070 1.10638078 2.06242479
C 4.28114914 0.29819364 0.14177467
H 3.47082597 0.32404352 -0.59640636
C 5.61220228 0.68722184 -0.48553847
H 5.56955991 1.73289565 -0.79083594
H 6.38829363 0.58798809 0.28796605
O 5.94201031 -0.06783023 -1.62448734
H 5.61895739 -0.96588131 -1.49102665
O 4.43440414 -1.06431620 0.62992498
C 3.43949560 -1.97253033 0.41609438
C 1.48836271 -3.96937401 0.04259970
C 3.22720200 -2.54054400 -0.85357865
C 2.67794716 -2.44702094 1.47976284
C 1.69523696 -3.41699664 1.29978168
C 2.27196504 -3.53527819 -1.03063601
H 1.10593286 -3.70748722 2.16001051
H 2.13338982 -3.99277809 -2.00165801
O 4.01678161 -2.06124694 -1.84081401
O 2.86053994 -1.90572460 2.73244232
C 3.65206236 -2.32682621 -3.18921647
H 4.31463199 -1.71324577 -3.79548899
H 2.61253697 -2.03596428 -3.36193363
H 3.79999631 -3.38212262 -3.43206556
C 4.04081331 -2.37111838 3.39734141
H 4.07059899 -1.86494861 4.36023818
H 3.97673270 -3.45156757 3.54738277
H 4.92777680 -2.12239439 2.81207745
C 0.49471628 -5.10941825 -0.20072559
C -0.49423768 -5.32908234 0.91001038
C -1.33113793 -3.01885310 -0.85456009
C -0.67564386 -3.81142568 -1.92492056
O -0.10916045 -5.06365531 -1.48368796
H 1.11105643 -6.01914980 -0.25459827
H -0.08780577 -5.57190284 1.88429843
H -0.75099280 -2.31217023 -0.27177521
H 0.09696318 -3.20662830 -2.40581226
H -1.40309459 -4.11041211 -2.68455491
C -2.69052792 -3.35618595 -0.32476270
O -2.61857752 -4.09660099 0.88519716
C -1.97637868 -5.37070727 0.73292438
H -3.23404703 -3.95600049 -1.07088989

H -2.23229028 -5.78763988 -0.24839362
H -2.41932097 -6.00279796 1.50267643
C -3.44069596 -2.06423744 -0.08676604
C -4.53701383 0.46494023 0.22248018
C -3.83787126 -1.34366770 -1.21152309
C -3.62575939 -1.55911151 1.19258045
C -4.18425149 -0.28691381 1.34314513
C -4.39374889 -0.07996918 -1.05546107
H -3.67150356 -1.76210254 -2.19607297
H -3.32419046 -2.15645818 2.04123313
O -4.78756881 0.72053059 -2.08103401
O -4.38220635 0.33090089 2.53635530
C -4.10969705 -0.40820801 3.71211563
H -4.37734804 0.24231385 4.54133676
H -3.04898514 -0.66846718 3.77980825
H -4.71061908 -1.32106801 3.74784963
C -4.58553175 0.23979326 -3.39848427
H -4.92792880 1.03113853 -4.06211324
H -5.17413946 -0.66481726 -3.57451827
H -3.52561652 0.03918540 -3.57912431
O -5.01252024 1.74177406 0.35376023
O 2.71509569 0.80067123 1.93288874
H 2.82059100 -0.08807784 2.30129928
C -4.03414132 2.73219289 0.72369131
H -3.43796952 2.34590683 1.55399206
C -4.85695391 3.90050299 1.23523069
H -4.19295420 4.71657764 1.52761585
H -5.51355369 4.25399728 0.43317708
O -5.58993863 3.52795035 2.38161495
H -6.00096256 2.67864733 2.18520703
C -3.15271837 3.11196071 -0.47590804
H -2.60703677 4.01826018 -0.18248488
O -3.95959307 3.49076264 -1.56649467
H -4.53245427 2.73312551 -1.75878284
C -2.11713884 2.05418070 -0.86869385
C -0.16251152 0.18555765 -1.62217768
C -1.41658006 1.32811053 0.10550693
C -1.81942322 1.83386051 -2.20667855
C -0.84615760 0.90329644 -2.58477510
C -0.46170058 0.39547092 -0.26427342
H -1.62260351 1.47878632 1.15696098
H -2.34850371 2.39942297 -2.96361153
H -0.60366289 0.73655336 -3.62790381
O 0.24670491 -0.39616166 0.58527363
O 0.79165124 -0.72172256 -1.96629641
H 1.14451091 -1.11078356 -1.15127796
C -0.03514978 -0.27997933 1.97111057
H 0.59201418 -1.01343481 2.47258737
H 0.22309667 0.71911001 2.32919122
H -1.09106979 -0.50391031 2.15504063

β-β' bottomC_{α}-C_{β}

O 2.66040799 4.60349874 -0.85924713
C 3.58221672 3.79730272 -0.26082776

C	5.63909030	2.38788154	0.95097994
C	4.82984403	4.41287097	-0.05631466
C	3.37328471	2.48594483	0.13502435
C	4.41392297	1.77294153	0.74193811
C	5.85066225	3.70774629	0.55132355
H	6.44433446	1.83831364	1.42609635
H	2.41233487	2.00348199	-0.00375755
H	6.80056658	4.20305887	0.70921706
C	1.37724000	4.04224306	-1.07299510
H	0.76060488	4.81882820	-1.52167632
H	0.94034274	3.72386287	-0.12380517
H	1.43527180	3.18084551	-1.74347441
O	5.02139006	5.69626457	-0.44779971
H	4.19873834	6.01230055	-0.84093671
C	4.21031446	0.32168181	1.08933900
H	5.05120056	-0.01163814	1.71223758
C	4.20634386	-0.56174705	-0.16618056
H	3.28456821	-0.39555362	-0.73696914
C	5.43164399	-0.35662645	-1.04320408
H	5.47258373	0.68311842	-1.36898738
H	6.32641593	-0.55880680	-0.43566836
O	5.41416489	-1.16253980	-2.19621167
H	4.98362900	-1.99478906	-1.96947985
O	4.25256760	-1.94920626	0.26305238
C	3.09676567	-2.66440399	0.33041088
C	0.78323004	-4.26913446	0.49621767
C	2.48418988	-3.16007943	-0.84027208
C	2.56086678	-3.02678571	1.56918443
C	1.43540969	-3.82074862	1.66400978
C	1.33698728	-3.93360266	-0.76690946
H	1.04668122	-4.06333679	2.64650929
H	0.90017901	-4.35053992	-1.66325929
O	3.11277668	-2.82352152	-1.98752744
O	3.12395669	-2.52100657	2.71825719
C	2.50921035	-3.17641176	-3.22431569
H	3.15171707	-2.75132816	-3.99194424
H	1.50924773	-2.74253618	-3.29749159
H	2.46722606	-4.26321266	-3.33670380
C	4.36352169	-3.13690163	3.08446643
H	4.69810519	-2.63734604	3.99155402
H	4.20062461	-4.19871942	3.28512376
H	5.10033160	-3.01112516	2.28963034
C	-0.38574302	-5.04953726	0.62445709
C	-3.51494994	-4.06848853	0.74164943
C	-2.58706266	-3.38274856	-0.20750689
C	-1.84922959	-4.36410679	-1.12881628
O	-1.17461735	-5.40717589	-0.41278063
H	-0.66914171	-5.46762380	1.58455472
H	-3.21736506	-4.65753450	1.59629789
H	-1.85257194	-2.73905483	0.29062560
H	-1.14849600	-3.80550360	-1.75749141
H	-2.56625518	-4.88439157	-1.76518903
C	-3.57713003	-2.56080300	-1.05865664
O	-4.78510730	-3.32299889	-1.04046474

C -4.91512628 -3.93052493 0.23964324
H -3.26433181 -2.52025305 -2.10559279
H -5.43423807 -4.88474030 0.10808382
H -5.53274647 -3.30058130 0.89953961
C -3.79284094 -1.14211687 -0.57498914
C -4.14218531 1.50891606 0.21040049
C -3.97753170 -0.15234588 -1.53329205
C -3.82476578 -0.82146937 0.78271322
C -4.01138072 0.50369121 1.17264703
C -4.15335044 1.17251058 -1.14245948
H -3.95009325 -0.42085367 -2.58075191
H -3.67720143 -1.59465901 1.52619423
O -4.28733153 2.21529001 -2.00420806
O -4.02350786 0.93141353 2.46241477
C -4.08568625 -0.04982250 3.47927443
H -4.19113933 0.49518363 4.41438093
H -3.17263535 -0.65230243 3.51129146
H -4.94822538 -0.70682232 3.33368416
C -4.26618723 1.92411703 -3.39113115
H -4.37237614 2.88011494 -3.89944083
H -5.09962140 1.27071228 -3.66242894
H -3.31859692 1.45766614 -3.67566261
O -4.24627852 2.82590728 0.56556712
O 2.99109935 0.17436131 1.79698181
H 3.01155733 -0.67998480 2.25054274
C -3.05449686 3.44022743 1.09457769
H -2.64672059 2.79813857 1.87867680
C -3.54831114 4.72491582 1.73449975
H -2.70351277 5.26655757 2.16466916
H -4.01146088 5.35336713 0.96662103
O -4.44424531 4.44661960 2.78813113
H -5.09087369 3.82063824 2.44464357
C -2.02004017 3.69516847 -0.01422241
H -1.24836148 4.33847660 0.42694546
O -2.59673051 4.44812077 -1.05534339
H -3.36197956 3.94379676 -1.37091601
C -1.35299968 2.41853901 -0.52940682
C -0.26319414 0.01639280 -1.47423421
C -0.90932031 1.43938625 0.37086355
C -1.18337060 2.20338073 -1.88932620
C -0.63556378 1.00699585 -2.36236313
C -0.38736355 0.24361732 -0.09290891
H -1.00751336 1.59515031 1.43734602
H -1.49486412 2.96916754 -2.58897969
H -0.50959267 0.82642898 -3.42374679
O 0.00529797 -0.80462262 0.68233547
O 0.20663377 -1.18128466 -1.91365515
H 0.45282387 -1.71293032 -1.14022265
C -0.03070023 -0.61191004 2.08653049
H 0.34751058 -1.53157759 2.52709689
H 0.61250844 0.22404886 2.37162632
H -1.06028857 -0.43540457 2.41646495

β-β’ bottomC_{α}-O

O 2.97673532 4.57079532 -1.88441300
C 3.66943681 4.01242684 -0.85024796
C 5.20161680 3.13229981 1.28448864
C 4.69292105 4.82787819 -0.34041995
C 3.42832393 2.76225030 -0.30454082
C 4.20413763 2.31539878 0.77019528
C 5.45250299 4.38503122 0.72711046
H 5.79453490 2.79609971 2.12864483
H 2.63087758 2.13548582 -0.68507812
H 6.22415423 5.03563854 1.11923260
C 1.81195007 3.89052027 -2.31770436
H 1.36588540 4.51133080 -3.09164352
H 1.10899719 3.76391052 -1.48991765
H 2.06488982 2.90942475 -2.73130214
O 4.92005926 6.04914732 -0.88154807
H 4.27626757 6.18549021 -1.58727712
C 3.96771763 0.93533457 1.33355186
H 4.29852265 0.91353470 2.37937427
C 4.75802684 -0.14419440 0.58047708
H 4.35977682 -0.24649668 -0.43642041
C 6.25630217 0.10882056 0.53577334
H 6.45118561 1.06376237 0.04787174
H 6.62782143 0.16748885 1.56978862
O 6.93936755 -0.88421743 -0.18923045
H 6.49165216 -1.72450936 -0.03468660
O 4.59714913 -1.40694394 1.27922381
C 3.60508875 -2.23665792 0.84339622
C 1.51208427 -3.86619839 -0.11327815
C 3.77605940 -3.00417471 -0.32695526
C 2.42178442 -2.37687017 1.56708634
C 1.37980940 -3.14984519 1.09380935
C 2.75660296 -3.81557897 -0.79056007
H 0.46434710 -3.16831079 1.66880075
H 2.87219412 -4.37790202 -1.70816325
O 4.98072360 -2.84848685 -0.92757166
O 2.25057164 -1.66908404 2.73807783
C 5.17795272 -3.44148491 -2.19973006
H 6.16566146 -3.12252432 -2.52429891
H 4.42423715 -3.09042032 -2.90972702
H 5.14437999 -4.53217644 -2.13079322
C 3.07313798 -2.12506376 3.81797287
H 2.83270482 -1.49664879 4.67376219
H 2.83524740 -3.16702225 4.04549286
H 4.12915541 -2.02625026 3.56296852
C 0.40661461 -4.54394174 -0.69819902
C -0.98657592 -4.17502498 -0.33416006
C -1.42408672 -2.78604441 -0.91538100
C -0.60862023 -2.26775110 -2.09419953
O -0.52748651 -3.12143319 -3.15788682
H 0.57366864 -5.12167435 -1.60105498
H -1.08835116 -4.10757844 0.75264530
H -1.38767081 -2.02496356 -0.13005033

H	0.43601836	-2.04949913	-1.79830266
H	-0.98009652	-1.28077805	-2.42161315
C	-2.89693766	-3.04763616	-1.34451900
O	-3.26956919	-4.27175310	-0.74131182
C	-2.11239071	-5.09021989	-0.81817148
H	-2.92402635	-3.17317205	-2.43684068
H	-1.93550931	-5.40531138	-1.85544911
H	-2.27311824	-5.96595669	-0.19074296
C	-3.78025367	-1.90370112	-0.94484773
C	-5.01394152	0.43980917	-0.12244948
C	-3.92535691	-0.85278580	-1.84350143
C	-4.29188850	-1.82338774	0.34685215
C	-4.92194499	-0.64795030	0.75170573
C	-4.54540932	0.32376097	-1.42942623
H	-3.53629905	-0.95342963	-2.84814413
H	-4.18454778	-2.67376927	1.00682781
O	-4.71301739	1.41932652	-2.21569980
O	-5.45247076	-0.43757041	1.98323162
C	-5.42520697	-1.51411384	2.90047105
H	-5.92310958	-1.15436372	3.79775122
H	-4.39676827	-1.80070144	3.14184622
H	-5.95956529	-2.38109374	2.50237171
C	-4.17790076	1.37109366	-3.52701676
H	-4.37049952	2.34824415	-3.96518543
H	-4.67571120	0.59972283	-4.12069045
H	-3.10024808	1.18468846	-3.49912114
O	-5.57642937	1.61780982	0.28667938
O	2.57614620	0.64819246	1.26810148
H	2.33652800	0.01682782	1.96309943
C	-4.77519219	2.40795973	1.18550490
H	-4.36226006	1.75206719	1.95608569
C	-5.76636569	3.35087243	1.84214072
H	-5.24102331	4.01102543	2.53532607
H	-6.24252811	3.96316567	1.06868697
O	-6.71756100	2.62901478	2.59381536
H	-7.01217308	1.89674812	2.04058421
C	-3.66700517	3.15030820	0.42242440
H	-3.28597159	3.92313860	1.10071590
O	-4.21688061	3.84241591	-0.67270340
H	-4.67870334	3.18419568	-1.21315840
C	-2.47354554	2.27844454	0.02258811
C	-0.14621020	0.84407167	-0.64407374
C	-1.90295250	1.38012031	0.93429447
C	-1.88894785	2.41811147	-1.22769770
C	-0.74040720	1.69596809	-1.55969332
C	-0.76207008	0.65975172	0.60774836
H	-2.34982191	1.24645143	1.91062668
H	-2.31960107	3.11301098	-1.93752874
H	-0.26907849	1.80808067	-2.53034938
O	-0.15328517	-0.24645745	1.42492897
O	0.99918132	0.18229392	-0.96352881
H	1.54993034	0.10621743	-0.15919315
C	-0.81302382	-0.57352287	2.63182558
H	-0.19962685	-1.33427737	3.10886969

H -0.88544903 0.29649906 3.29150389

H -1.81777014 -0.96232210 2.42667573

β-β’ bottomC_{β}-C_{γ}

O 2.08987724 4.49212483 -0.97073575
C 3.18893782 3.91052192 -0.41014502
C 5.56515001 2.99957754 0.68992617
C 4.32571807 4.73799841 -0.40958860
C 3.24988493 2.63429085 0.12636931
C 4.45220548 2.17195097 0.67715979
C 5.50690119 4.28089438 0.14264825
H 6.49323694 2.64820649 1.12818115
H 2.37829758 1.98852600 0.14371933
H 6.36656233 4.93930225 0.14698301
C 0.85988670 3.79767960 -0.86178252
H 0.10457241 4.42950538 -1.32480718
H 0.61098669 3.62821404 0.18982500
H 0.89823344 2.83692213 -1.38316097
O 4.25396525 5.98392178 -0.93839954
H 3.35195096 6.11918091 -1.25356876
C 4.52574529 0.76113139 1.20809541
H 5.40466330 0.67230042 1.86182836
C 4.69524912 -0.24784316 0.05961576
H 3.86283952 -0.14544842 -0.64543519
C 6.03139829 -0.08982579 -0.64827244
H 6.13490012 0.93846974 -0.99618926
H 6.82550665 -0.28693766 0.08686346
O 6.16927325 -0.92825236 -1.76948747

H 5.70647341 -1.75253336 -1.58098619
O 4.68255582 -1.59337876 0.61006187
C 3.51558017 -2.29820220 0.52276937
C 1.08735606 -3.67900114 0.36438164
C 3.04231767 -2.76740875 -0.71489518
C 2.80052841 -2.62094981 1.67228051
C 1.59290370 -3.30757584 1.60094716
C 1.82957652 -3.44501471 -0.79298221
H 1.04332538 -3.49229659 2.51797255
H 1.44374147 -3.78776368 -1.74382013
O 3.83268141 -2.48968446 -1.77677980
O 3.24487445 -2.16985894 2.89400621
C 3.30354964 -2.68345209 -3.08174222
H 4.05124916 -2.28082852 -3.76128891
H 2.36271186 -2.13843306 -3.19591629
H 3.15260411 -3.74704563 -3.28401301
C 4.39490934 -2.86502248 3.38904705
H 4.63892679 -2.41114725 4.34752708
H 4.15361626 -3.92134272 3.52962841
H 5.23102759 -2.75709567 2.69613249
C -0.34514110 -4.17646886 0.25679261
C -1.26097510 -2.99335871 0.19253420
C -2.05115483 -3.01065831 -1.06660972
C -1.37867530 -4.15740128 -1.84552059

O -0.58580115 -4.90972379 -0.93213958

H -0.57123487 -4.83129655 1.10910775

H -1.34227722 -2.25329466 0.97389515

H -1.92508037 -2.07777662 -1.62794113

H -0.73427168 -3.73463143 -2.62487485

H -2.08956431 -4.84003430 -2.31532889

C -3.58098667 -3.12187781 -0.78802196

O -3.87349991 -4.01791414 0.27090769

C -3.64259757 -5.32844220 0.02065699

H -4.08835612 -3.46768009 -1.69983345

H -3.80060786 -5.68600229 -0.99214196

H -3.84588453 -5.96872201 0.86624314

C -4.08678814 -1.75211578 -0.40489162

C -4.63862561 0.89420459 0.23419478

C -4.34471565 -0.83790213 -1.42205234

C -4.15252762 -1.37283482 0.93119996

C -4.44068069 -0.04479994 1.24838964

C -4.62495673 0.48682209 -1.10029548

H -4.29172492 -1.15791387 -2.45481360

H -3.97516517 -2.11317317 1.69806106

O -4.86747437 1.46496107 -2.01216810

O -4.52344751 0.44633688 2.51030075

C -4.34937136 -0.46090819 3.58218790

H -4.47227924 0.12430962 4.49035624

H -3.34920206 -0.90490432 3.56373880

H -5.10052941 -1.25484720 3.54923739

C -4.80679150 1.11164387 -3.38220254

H -4.99524446 2.02804875 -3.93750665

H -5.57403802 0.37189960 -3.62657678

H -3.81757671 0.72130031 -3.63961101

O -4.88204775 2.20801306 0.52802860

O 3.34559040 0.49769796 1.93843363

H 3.43975115 -0.35303645 2.38958841

C -3.76825248 2.96405663 1.04070335

H -3.25070338 2.36307630 1.79234699

C -4.40576669 4.15784592 1.72652858

H -3.62611078 4.80537726 2.13303891

H -4.98331687 4.72692001 0.98971200

O -5.20864667 3.74263141 2.80927266

H -5.74579482 3.00761784 2.49315713

C -2.81872988 3.36892668 -0.09945732

H -2.16023779 4.14985352 0.30199016

O -3.54199262 3.97305894 -1.14336715

H -4.24060448 3.35099909 -1.39592800

C -1.93679165 2.21995622 -0.58539678

C -0.29590810 0.12113746 -1.45486021

C -1.18990540 1.47477074 0.33871320

C -1.82015438 1.92595915 -1.93521823

C -1.01523170 0.86895866 -2.36838996

C -0.36466305 0.44555710 -0.08687644

H -1.23447802 1.71929016 1.39298471

H -2.35951788 2.52773683 -2.65537079

H -0.92195083 0.62656085 -3.42093961

O 0.43236399 -0.31497787 0.71006992

O 0.48855532 -0.90730609 -1.86807774
H 0.87759056 -1.33127826 -1.08676568
C 0.40031903 -0.04957439 2.10209235
H 1.06382580 -0.77315405 2.56781662
H 0.77067208 0.95695353 2.31163140
H -0.61848891 -0.17262417 2.48764630

β-β' bottomC$_{\gamma}$-O

O 1.89267601 4.58667327 -1.13584084
C 2.78954945 4.09432867 -0.23600256
C 4.73433924 3.36938837 1.60415693
C 3.68160075 5.06132646 0.26247169
C 2.87434988 2.77920295 0.18903121
C 3.86148085 2.41105922 1.11340375
C 4.64652050 4.69624182 1.18089743
H 5.49585610 3.08703790 2.32274990
H 2.17722007 2.03080053 -0.17246542
H 5.32019116 5.45689833 1.55521114
C 1.02054313 3.65692401 -1.75714153
H 0.39140064 4.23004839 -2.43435465
H 0.39580020 3.15369391 -1.01575171
H 1.58886399 2.91093765 -2.32073056
O 3.58831834 6.34562972 -0.15966998
H 2.86890956 6.39507827 -0.80089826
C 3.98563018 0.96354094 1.51469257
H 4.74054006 0.87717710 2.30828770
C 4.45414085 0.11337514 0.32560917
H 3.74482099 0.21192479 -0.50272924
C 5.85975230 0.47759685 -0.12188765
H 5.90502214 1.54779839 -0.32903804
H 6.54500328 0.25872612 0.70980680
O 6.26005187 -0.19663676 -1.29080274
H 5.85489350 -1.07101129 -1.28135428
O 4.50329996 -1.27793831 0.73811969
C 3.45951005 -2.08769941 0.40072443
C 1.33877082 -3.81252871 -0.24688030
C 3.21553093 -2.44545747 -0.93783941
C 2.65472151 -2.63925517 1.38985476
C 1.60694030 -3.50370679 1.07820571
C 2.15335004 -3.28313832 -1.25444637
H 1.00919315 -3.88081169 1.89873037
H 1.92703503 -3.52461134 -2.28494801
O 4.07025447 -1.91173991 -1.83972789
O 2.84300692 -2.26059644 2.69934748
C 3.78411131 -2.07407218 -3.22350702
H 4.54796504 -1.50250530 -3.74567954
H 2.79364543 -1.67640166 -3.45693750
H 3.84852317 -3.12716948 -3.50945925
C 3.98366822 -2.86079162 3.32257381
H 4.01662421 -2.47527677 4.33967447
H 3.86428509 -3.94688276 3.34193415
H 4.89495245 -2.59024856 2.78650779

C 0.20242669	-4.72499355	-0.69179804	
C -0.96923161	-4.86640648	0.28689540	
C -1.65849863	-3.49656269	0.04052097	
C -1.45210680	-3.29791391	-1.47558149	
O -0.41524685	-4.20035013	-1.86339632	
H 0.61188089	-5.69999321	-0.96885815	
H -0.66191142	-4.95141575	1.32964603	
H -1.12144203	-2.72111207	0.59294685	
H -1.17118414	-2.26853273	-1.71375570	
H -2.34160652	-3.56888972	-2.05060494	
C -3.15349196	-3.43098330	0.48485972	
O -3.15332732	-3.96434400	1.74237954	
C -1.82379938	-6.02974260	-0.08703345	
H -3.71513584	-4.08608083	-0.19999379	
H -1.83134602	-6.38914958	-1.10802864	
H -2.52133383	-6.44364208	0.62667731	
C -3.67003928	-2.00201881	0.42989198	
C -4.51200334	0.64377342	0.32944040	
C -3.98181724	-1.42553309	-0.79882546	
C -3.80265936	-1.28156217	1.61187657	
C -4.23842027	0.04223747	1.55858602	
C -4.39766154	-0.09553030	-0.84822856	
H -3.89976316	-2.00658137	-1.70733276	
H -3.57886328	-1.76722103	2.55121297	
O -4.69053853	0.57656142	-1.98930349	
O -4.40523216	0.84625163	2.63775008	
C -4.11628098	0.30742007	3.91527761	
H -4.30829352	1.10830760	4.62509554	
H -3.06830968	0.00000288	3.98326411	
H -4.76318155	-0.54606755	4.13544016	
C -4.39373454	-0.06315580	-3.21933355	
H -4.59070549	0.67352615	-3.99537247	
H -5.03898728	-0.93292721	-3.37115926	
H -3.34125781	-0.35979698	-3.25431920	
O -4.90737449	1.95020009	0.27228728	
O 2.73003318	0.51420734	1.98552488	
H 2.83971343	-0.36740019	2.36962656	
C -3.88399306	2.93402559	0.52561118	
H -3.25317380	2.59160799	1.34930355	
C -4.64336511	4.16103071	0.99568757	
H -3.94327210	4.98093216	1.16898076	
H -5.35036633	4.46303372	0.21561571	
O -5.29633469	3.90405491	2.21955521	
H -5.70988512	3.03673750	2.14135215	
C -3.07057878	3.19103343	-0.74721181	
H -2.50050334	4.11326318	-0.57854243	
O -3.94214965	3.47543813	-1.81898006	
H -4.52156628	2.70429607	-1.90745416	
C -2.06879019	2.09313736	-1.11471538	
C -0.13411256	0.21453343	-1.88396704	
C -1.42546736	1.30448585	-0.15060080	
C -1.72981072	1.92441273	-2.45236039	
C -0.76301174	0.99482962	-2.83701136	
C -0.48338534	0.35824870	-0.53097731	

H -1.66343267 1.41004440 0.89885619

H -2.22414431 2.53272824 -3.19957379

H -0.48531408 0.86742997 -3.87697898

O 0.16674094 -0.50079120 0.30118599

O 0.80972287 -0.69211722 -2.24511053

H 1.08850314 -1.17798268 -1.45329159

C -0.08465643 -0.37631778 1.69365707

H 0.51847154 -1.14088453 2.17943038

H 0.23047405 0.60598820 2.05142733

H -1.14614055 -0.55013951 1.89982918

β-β′ bottomC_{α}-C_{Ph} α-side

C -4.49685634 -0.10323774 1.03071694

C -3.31366151 0.62284631 0.49177564

C -3.13139514 -0.06004669 -0.87495443

C -4.57874700 -0.41154167 -1.23547082

O -5.27749694 -0.56105597 0.01149590

H -5.03438327 0.12396862 1.94208371

H -2.43056254 0.52929651 1.11979610

H -2.51043311 -0.95254328 -0.80631291

H -4.67410563 -1.34511025 -1.78863326

H -5.05741515 0.39746449 -1.79675424

C -2.48802428 1.04400192 -1.71676060

O -3.07794869 2.24957566 -1.25740634

C -3.55074399 2.09889573 0.08243109

H -2.72612644 0.95283138 -2.78100020

H -4.61363269 2.35511075 0.09881854

H -3.01442743 2.79006752 0.73984721

C -0.98183997 1.02075713 -1.52469251

C 1.73869344 0.78229792 -1.00164583

C -0.25863325 -0.02427098 -2.09416565

C -0.35147206 1.97430079 -0.73515408

C 1.01611826 1.85345871 -0.47688519

C 1.10469577 -0.13654012 -1.84163151

H -0.76949654 -0.75271786 -2.71140691

H -0.92706496 2.80505396 -0.35166655

O 1.90251820 -1.11543981 -2.34153185

O 1.73547103 2.71226205 0.29155647

C 1.07511573 3.85944754 0.78934751

H 1.82698378 4.42225437 1.33751674

H 0.25917420 3.58424544 1.46547569

H 0.67896386 4.46940541 -0.02732258

C 1.29655796 -2.09750403 -3.16294882

H 2.08737618 -2.79567232 -3.42916495

H 0.88768881 -1.64457082 -4.07037357

H 0.50678648 -2.62459681 -2.62007409

O 3.07315437 0.63588874 -0.73745011

C 3.41835191 0.21282259 0.59599410

H 2.82102068 0.78437343 1.31058293

C 4.87263024 0.61527506 0.75376465

H 5.23222504 0.31014041 1.73861668

H 5.46942592 0.10456408 -0.00962430

O 5.01451171 2.01698397 0.67393143
H 4.49642192 2.30616841 -0.08555204
C 3.19789629 -1.30013367 0.76010643
H 3.70922372 -1.59756999 1.68316721
O 3.84474323 -1.99449540 -0.27929460
H 3.49579797 -1.63772321 -1.10959708
C 1.72424076 -1.68523910 0.88803587
C -0.99896056 -2.34685786 1.07420889
C 0.88077518 -0.98402969 1.75884617
C 1.19876466 -2.74380386 0.16123532
C -0.15645475 -3.06541733 0.24382628
C -0.46075613 -1.31458533 1.85956157
H 1.27493125 -0.17674989 2.36322603
H 1.85227937 -3.31419686 -0.48640054
H -0.57780967 -3.88245158 -0.33068938
O -1.35943246 -0.71038938 2.69540685
O -2.31908241 -2.65362527 1.13037599
H -2.78092440 -2.00895027 1.68601886
C -0.95296740 0.48539751 3.32980436
H -1.82925927 0.86896645 3.84869174
H -0.15569985 0.29758758 4.05427198
H -0.60842959 1.21595896 2.58771693

β-β′ bottomC_{α}-C_{Ph} phenyl-side

O -4.32165487 1.16064687 -1.65963931
C -3.74380788 0.32665196 -0.74468529
C -2.83923744 -1.41211676 1.21583405
C -4.63515242 -0.15655656 0.22777215
C -2.41257322 -0.05226082 -0.72528833
C -1.95295973 -0.92673977 0.26588420
C -4.17920792 -1.02522299 1.20167299
H -2.49019220 -2.09969706 1.97889806
H -1.71819472 0.28836921 -1.48267816
H -4.88451509 -1.39754689 1.93414777
C -3.49548031 1.66868423 -2.68919711
H -4.13330591 2.29110802 -3.31234133
H -3.07538819 0.85517471 -3.28709024
H -2.68455766 2.27349032 -2.27321841
O -5.93799512 0.21850723 0.20079909
H -6.06156722 0.81009017 -0.55110937
C -0.48722472 -1.28724976 0.29428379
H -0.35471884 -2.20133598 0.88967027
C 0.33865460 -0.17541018 0.96040274
H 0.24294466 0.74895494 0.38061248
C -0.04961483 0.05815412 2.41178144
H -1.10963949 0.30665257 2.46814237
H 0.11465667 -0.87928416 2.96334872
O 0.66272777 1.11826219 3.00369819
H 1.55293386 1.11413372 2.63454286
O 1.73287090 -0.57602092 0.98239100
C 2.56018604 -0.11142471 -0.00177498
C 4.29165222 0.76647484 -1.90613749

C 2.96515384 1.23552350 -0.01641005
C 3.07856564 -0.99581302 -0.94616392
C 3.96840686 -0.56173434 -1.93403465
C 3.84261861 1.69870636 -1.00064949
H 4.34225525 -1.26376176 -2.66870516
H 4.16181496 2.73187910 -1.03784770
O 2.45073144 1.99720800 0.97750013
O 2.66606000 -2.30523824 -0.93766968
C 2.71483196 3.38904104 0.96594852
H 2.15868906 3.80129812 1.80466281
H 2.36594101 3.84058153 0.03329141
H 3.78278174 3.58424174 1.09673247
C 3.24582608 -3.09267064 0.10734238
H 2.83930397 -4.09592652 -0.00373614
H 4.33196352 -3.11888241 -0.01062877
H 2.98123779 -2.68320760 1.08368626
O -0.05637437 -1.49404461 -1.03422470
H 0.80280641 -1.93711501 -1.02270363

β-O-4 bottomC_{α}-C_{β} α-side

O 1.90198698 1.20309115 0.00000027
C 0.88335472 0.29187159 0.00000110
C -0.96110262 -1.79049985 0.00000142
C 1.32554171 -1.04529116 0.00000028
C -0.46107417 0.59495851 0.00000173
C -1.42091992 -0.45319713 0.00000013
C 0.39241962 -2.07444482 0.00000038
H -1.68110577 -2.60072230 0.00000172
H -0.80885632 1.61849128 0.00000150
H 0.75397751 -3.09540035 -0.00000033
C 1.55157344 2.57276574 -0.00000093
H 2.48734180 3.12685367 -0.00000449
H 0.97354800 2.82568092 0.89332547
H 0.97354281 2.82567786 -0.89332468
O 2.65306222 -1.32593686 -0.00000108
H 3.13229317 -0.48871758 -0.00000852
C -2.79530303 -0.17230189 -0.00000330
H -3.54970455 -0.94804944 -0.00000329
O -3.20561013 1.12580232 0.00000806
H -4.16348763 1.16936704 -0.00005023

β-O-4 bottomC_{α}-C_{β} β-side

C -3.70358450 -2.89712055 0.78342462
H -2.71961815 -2.49508112 0.55769515
C -4.20194007 -4.16787068 0.20205799
H -3.43353604 -4.93827719 0.27094688
H -5.08493069 -4.49997888 0.76141275
O -4.52365423 -4.02030722 -1.17905751
H -4.90309282 -3.13960023 -1.28784554
O -4.70300293 -1.96367088 0.95531617
C -4.31906442 -0.65808439 0.75757413
C -3.32966446 1.88771830 0.22503587

C -4.31238440 -0.16583284 -0.55608584
C -3.89568802 0.15132253 1.80867732
C -3.40800174 1.42936594 1.53001944
C -3.81817882 1.10808058 -0.82519983
H -3.06196257 2.02881385 2.36500889
H -3.78990109 1.50121510 -1.83151943
O -4.75486256 -1.03318123 -1.49755503
O -3.83204700 -0.24971340 3.10167193
C -4.60646628 -0.66123224 -2.86257580
H -4.96166476 -1.51211611 -3.43953048
H -3.55567476 -0.47250436 -3.09403804
H -5.21532176 0.21722467 -3.09074982
C -4.79564409 -1.17760958 3.60211765
H -4.83566965 -1.00454430 4.67603177
H -5.77514209 -0.99260202 3.15783899
H -4.49309649 -2.20519701 3.39959344
C -2.65070828 3.21833018 -0.06127317
C -1.19309914 3.19275748 0.40621555
C -0.44824748 2.58838286 -0.79599479
C -1.38242471 2.90124461 -1.98160965
O -2.56828039 3.49958591 -1.44615622
H -3.22021624 4.03355321 0.39533021
H -1.04413308 2.61132077 1.31652827
H -0.30776877 1.51351445 -0.69257924
H -1.63586299 1.98492487 -2.52310728
H -0.95104132 3.61809656 -2.68364705
C 0.92392745 3.31575493 -0.78302199
O 0.84427302 4.30646501 0.23446140
C -0.52199366 4.55969051 0.51388501
H 1.10141325 3.80546449 -1.74886287
H -0.94901814 5.25148172 -0.22497840
H -0.58325166 5.01026624 1.50469218
C 2.05312275 2.35409829 -0.49820942
C 3.92517578 0.36476570 0.00828536
C 2.58054309 1.61983840 -1.55701416
C 2.47652201 2.13289304 0.80710184
C 3.42334659 1.13814846 1.05587722
C 3.52337397 0.62636827 -1.30303107
H 2.23800411 1.81556659 -2.56528012
H 2.07483522 2.74925485 1.59914394
O 4.09868283 -0.15299383 -2.25465892
O 3.90738115 0.82170445 2.28515818
C 3.48996969 1.61482650 3.37975376
H 4.01549769 1.22448358 4.24807578
H 2.41011424 1.53267454 3.53853113
H 3.75551020 2.66441758 3.22639775
C 3.67296157 0.02679632 -3.59376025
H 4.21962318 -0.70670431 -4.18281456
H 3.91689899 1.03290338 -3.94600132
H 2.59814812 -0.15372994 -3.68621815
O 4.83129345 -0.63234680 0.24651773
C 4.31790942 -1.82168893 0.87813934
H 3.65196660 -1.52955490 1.69352687
C 5.54146298 -2.48616429 1.48091482

H 5.25178850 -3.42493525 1.95758381
H 6.25856287 -2.70593121 0.68288872
O 6.10996937 -1.66826726 2.48078896
H 6.14112854 -0.77331812 2.12412456
C 3.60032226 -2.71098645 -0.14818931
H 3.45263068 -3.68895438 0.32468261
O 4.44591695 -2.94178054 -1.25093731
H 4.67826374 -2.07021556 -1.60461242
C 2.23218373 -2.17720463 -0.57753121
C -0.24386379 -1.15872765 -1.40875753
C 1.34826848 -1.61645722 0.35382255
C 1.84086736 -2.25399100 -1.90705675
C 0.61035591 -1.74492113 -2.32345218
C 0.12596703 -1.10908933 -0.05568809
H 1.62048927 -1.55739038 1.39922461
H 2.51065639 -2.70652112 -2.62714084
H 0.29947468 -1.79995782 -3.36025322
O -0.80713711 -0.52770840 0.75858357
O -1.43299232 -0.63422842 -1.80847142
H -1.89048410 -0.27170012 -1.03444851
C -0.45202009 -0.34105556 2.11722667
H -1.29951242 0.14689577 2.59227084
H -0.27151821 -1.30254240 2.60679737
H 0.44325321 0.28674661 2.18993968

β-O-4 bottomC_{α}-C_{Ph} α-side

C -2.87734173 -3.39758106 1.27265367
H -2.36856147 -4.33217245 1.07160006
C -3.63739775 -2.75514579 0.15998592
H -2.97347490 -2.25727325 -0.56488431
C -4.49469660 -3.79057050 -0.55119994
H -3.87484800 -4.65575824 -0.79799081
H -5.27209144 -4.11548440 0.15200680
O -5.05297384 -3.31703149 -1.75086311
H -5.31061062 -2.39853724 -1.60745299
O -4.54536949 -1.73669101 0.68410221
C -4.14131659 -0.43962418 0.54806106
C -3.11492504 2.15284090 0.28034699
C -4.15473453 0.20310286 -0.69937667
C -3.71673753 0.28230614 1.66200986
C -3.21337330 1.56957249 1.53799078
C -3.62832871 1.48786964 -0.83155129
H -2.85115952 2.06989642 2.42960078
H -3.59327328 1.97642124 -1.79555001
O -4.65165488 -0.52394448 -1.72531198
O -3.68090392 -0.33436078 2.89121666
C -4.42847296 -0.06262625 -3.05267062
H -4.79754873 -0.85367632 -3.70162996
H -3.36043242 0.08878833 -3.22501970
H -4.98463602 0.85945609 -3.23930329
C -4.97087438 -0.57820900 3.46997297
H -4.78812134 -1.06573056 4.42526047

H	-5.48445323	0.37268172	3.62826655
H	-5.56019241	-1.22766374	2.82147352
C	-2.37254485	3.46916003	0.11134321
C	-0.91792985	3.32842369	0.57019488
C	-0.20499422	2.78651977	-0.68021052
C	-1.11976069	3.24714102	-1.83218390
O	-2.27313641	3.86134145	-1.24508583
H	-2.90007833	4.27075155	0.63736235
H	-0.79847348	2.66684091	1.42884855
H	-0.12190893	1.70127037	-0.66659619
H	-1.42146406	2.39349625	-2.44699924
H	-0.65066124	3.99565057	-2.47425516
C	1.20452196	3.43352487	-0.61110196
O	1.17384682	4.34909313	0.47734058
C	-0.17712251	4.64608125	0.78536249
H	1.41406866	3.98369736	-1.53716768
H	-0.57050923	5.41731312	0.10917312
H	-0.21319182	5.01586591	1.81034364
C	2.28032617	2.39358522	-0.39823828
C	4.06421561	0.29376202	-0.03673279
C	2.74487528	1.68619971	-1.50448627
C	2.72172335	2.08708834	0.88314881
C	3.62421032	1.03650278	1.05914568
C	3.64480945	0.63854418	-1.32344644
H	2.39005533	1.94819008	-2.49342081
H	2.36931940	2.68169643	1.71417126
O	4.16152603	-0.11806856	-2.32549223
O	4.12016088	0.63675210	2.25894287
C	3.75734874	1.38540343	3.40315778
H	4.28014673	0.92512582	4.23822685
H	2.67786041	1.34082676	3.57835207
H	4.06635657	2.42946017	3.30209911
C	3.71265921	0.14571700	-3.64270251
H	4.21043838	-0.58196093	-4.28030336
H	3.99434021	1.15584920	-3.95304018
H	2.62881461	0.01811176	-3.71522194
O	4.92770768	-0.75425657	0.12961890
O	-2.26535079	-2.65243573	2.22040793
H	-2.80055092	-1.87313796	2.45028679
C	4.37580315	-1.94830808	0.71976662
H	3.73883801	-1.66499802	1.56073953
C	5.58088896	-2.68974540	1.26753332
H	5.25977459	-3.63558101	1.70874284
H	6.27270349	-2.90404241	0.44605517
O	6.20312150	-1.94164559	2.28989926
H	6.26144265	-1.03287677	1.97385082
C	3.60272412	-2.75970208	-0.33021575
H	3.41821927	-3.74981884	0.10256142
O	4.42104459	-2.97898612	-1.45641014
H	4.68415424	-2.10327171	-1.77649875
C	2.25535036	-2.14579248	-0.71581682
C	-0.16763084	-0.96406272	-1.47292435
C	1.42086710	-1.56341345	0.24731205
C	1.83887592	-2.16376338	-2.03980448

C 0.63179160 -1.57523126 -2.41926574
C 0.22602795 -0.97026335 -0.12566139
H 1.71156870 -1.55161678 1.28904140
H 2.47108129 -2.63003059 -2.78460899
H 0.30066167 -1.58291226 -3.45130350
O -0.64747654 -0.34207623 0.71548863
O -1.33241003 -0.35742283 -1.83382716
H -1.74310449 0.01006650 -1.03671183
C -0.27718241 -0.25894726 2.08124130
H -1.09215075 0.25560626 2.58675106
H -0.16580722 -1.25722956 2.51002927
H 0.65247845 0.31241728 2.18307650

β-O-4 bottomC_{α}-C_{Ph} phenyl-side

O -1.70204643 0.39151922 0.00005131
C -0.42685787 -0.09391925 -0.00007434
C 2.28585693 -0.79248833 0.00008171
C 0.56516567 0.90282983 -0.00003496
C -0.07043026 -1.44051089 -0.00012617
C 1.27967787 -1.71938930 0.00002234
C 1.90522306 0.55645076 -0.00000111
H 3.33205158 -1.07151376 0.00023157
H -0.81442357 -2.22683702 -0.00025417
H 2.64251093 1.35038315 0.00000482
C -2.75833160 -0.54885512 0.00006585
H -3.68028655 0.02748828 0.00001058
H -2.71814988 -1.17721824 0.89443145
H -2.71812830 -1.17732283 -0.89423269
O 0.20602457 2.21240869 -0.00002101
H -0.75722212 2.25889099 -0.00003385

β-O-4 bottomC_{β}-O β-side

O 2.96719965 0.78764196 -0.63221133
C 1.87015938 0.14746969 -0.13046507
C -0.18835284 -1.39215193 0.90730844
C 2.05738075 -1.23101615 0.06614620
C 0.66060921 0.74458681 0.18193212
C -0.37760960 -0.03192920 0.70682277
C 1.02731280 -1.99371779 0.58488626
H -0.98824580 -1.99451551 1.32561082
H 0.50364659 1.80698178 0.05124943
H 1.19514063 -3.05203407 0.74109957
C 2.86672899 2.18219636 -0.84722794
H 3.83122961 2.50060929 -1.23547292
H 2.65525995 2.70506994 0.08960741
H 2.08391084 2.40687462 -1.57722866
O 3.24586121 -1.80592491 -0.24141450
H 3.83167205 -1.11713342 -0.57768506
C -1.73123187 0.60019049 0.97491194
H -2.21093759 0.03582602 1.78734191
C -2.57779515 0.51406040 -0.25513077
H -2.37971675 1.23618257 -1.04377761

C -3.25168807 -0.75785010 -0.63505078

H -2.55915811 -1.41888476 -1.17081931

H -3.57135230 -1.29808829 0.26788143

O -4.33951145 -0.55351729 -1.52093186

H -4.93313488 0.08626155 -1.11877703

O -1.52513768 1.94936805 1.36638598

H -2.37868985 2.39127625 1.36154482

β-O-4 bottomC_{β}-O_{O}-side

O -4.89219304 -2.98884645 0.59513124

C -4.60377471 -1.78754210 0.51479953

C -3.82563406 0.94444430 0.26054710

C -4.52975418 -1.10781183 -0.78628570

C -4.30102637 -0.95596588 1.67870903

C -3.92573548 0.36697885 1.52465087

C -4.14954551 0.21639131 -0.89580107

H -3.69869944 0.93851108 2.41856728

H -4.10259337 0.71652253 -1.85311758

O -4.83779882 -1.90103009 -1.80897263

O -4.29930196 -1.42584154 2.93139580

C -4.73165655 -1.36830700 -3.12091999

H -5.01079564 -2.17798750 -3.78979905

H -3.70402212 -1.05483204 -3.32327119

H -5.41768551 -0.52705521 -3.25240598

C -4.91486700 -2.66853589 3.29082489

H -4.93503838 -2.65635111 4.37863507

H -5.92660495 -2.72863839 2.89176066

H -4.33526917 -3.51228143 2.92335160

C -3.33766032 2.37638982 0.15010731

C -1.90273271 2.51868227 0.66610390

C -1.04574723 2.23264633 -0.57751915

C -1.99871820 2.55303756 -1.74510782

O -3.28454239 2.84202191 -1.18330545

H -4.03135064 3.03061005 0.68958052

H -1.67302544 1.84369571 1.49119663

H -0.72372822 1.19445280 -0.62317667

H -2.07243591 1.69978942 -2.42572247

H -1.69191557 3.43381525 -2.31354040

C 0.18081164 3.16858376 -0.40203815

O -0.09347884 3.98533735 0.73013813

C -1.48496977 3.95110158 0.99189576

H 0.30146894 3.80683327 -1.28627332

H -2.02108285 4.65757281 0.34332250

H -1.63802159 4.23416478 2.03359816

C 1.44562273 2.37254631 -0.18396790

C 3.59745847 0.65060482 0.16189484

C 2.12181196 1.88471938 -1.29858114

C 1.85472754 2.04298771 1.10275641

C 2.94186296 1.18433967 1.27207894

C 3.20443666 1.02584292 -1.12427899

H 1.78629926 2.16165537 -2.28998681

H 1.33085060 2.47307533 1.94490607

O 3.92947101 0.48383493 -2.13638071

O 3.42658060 0.77935257 2.47460306
C 2.84945185 1.33992021 3.63814539
H 3.40046550 0.91967745 4.47616961
H 1.79196577 1.07072822 3.72423770
H 2.94798171 2.42895920 3.63819968
C 3.52539336 0.77967368 -3.46155804
H 4.19789537 0.22342091 -4.11124525
H 3.62194862 1.84962990 -3.66587724
H 2.49502710 0.45581682 -3.63326130
O 4.63897839 -0.22272297 0.31738652
C 4.29075941 -1.55111045 0.75532046
H 3.56468527 -1.47571051 1.56820581
C 5.58164245 -2.11095381 1.32322402
H 5.42003860 -3.13765604 1.65802480
H 6.34535270 -2.11520675 0.53831394
O 5.99533290 -1.36555763 2.44792231
H 5.91292964 -0.43501494 2.21066992
C 3.74651062 -2.38152214 -0.41640035
H 3.73356654 -3.42699415 -0.08673403
O 4.65159325 -2.33112299 -1.49498385
H 4.75918372 -1.39441792 -1.71802330
C 2.32682216 -2.00038017 -0.84259070
C -0.25437115 -1.27719355 -1.66428377
C 1.33926132 -1.69987200 0.10512020
C 1.99252177 -1.96412350 -2.18930774
C 0.70918261 -1.60177504 -2.60016111
C 0.06483964 -1.33603102 -0.29859720
H 1.56932867 -1.73108577 1.16178733
H 2.74580097 -2.21425802 -2.92547611
H 0.44097887 -1.57288410 -3.64990965
O -0.96843154 -1.00253303 0.53355403
O -1.50122874 -0.90150258 -2.05641906
H -2.04838397 -0.76895522 -1.26749845
C -0.69143943 -0.96102939 1.92120663
H -1.61883327 -0.66986435 2.40930867
H -0.38400909 -1.94514539 2.28637173
H 0.09497347 -0.22612808 2.12863723

β-O-4 bottomO-C_{PhO}-side

O -3.01604171 0.99100755 0.69698603
C -2.02326485 0.24718839 0.12997232
C -0.20504045 -1.48333703 -1.05024078
C -2.38223751 -1.09124371 -0.11032968
C -0.76228381 0.70585660 -0.20923221
C 0.15657944 -0.16772403 -0.80230501
C -1.47248726 -1.94864176 -0.70004284
H 0.50031398 -2.15862229 -1.52264153
H -0.47830042 1.73909737 -0.05481643
H -1.77280968 -2.97166986 -0.88870717
C -2.74167096 2.35382448 0.96205846
H -3.64722718 2.76679483 1.39966555

H -2.50142437 2.88771825 0.03858328

H -1.91363123 2.45173883 1.66984209

O -3.61795601 -1.53181931 0.22442938

H -4.10597104 -0.79321932 0.60814563

C 1.54828661 0.32238601 -1.10049415

H 2.01419682 -0.34754912 -1.83609220

C 2.43064135 0.30200076 0.17144083

H 1.96125369 0.99758733 0.89934982

C 2.59559867 -1.06380270 0.84797501

H 1.63461555 -1.41251972 1.22519032

H 2.95535086 -1.78733196 0.10294293

O 3.47461961 -0.97024484 1.93981011

H 4.26329098 -0.50791108 1.63656831

O 3.64411892 0.88181636 -0.08155696

O 1.49425458 1.64042201 -1.59581391

H 2.39365167 1.98739047 -1.58167950

β-O-4 bottomO-C$_{Ph}$ phenyl-side

C -4.64750006 -1.81728856 0.89405609

C -3.98975243 0.75459731 0.26836025

C -4.66256088 -1.46007638 -0.44225041

C -4.33505621 -0.96385776 1.92399894

C -4.01006864 0.35915385 1.59746185

C -4.32748797 -0.13669164 -0.75592153

H -3.76692195 1.04540982 2.40226888

H -4.33583574 0.21880742 -1.77712314

O -4.98513409 -2.41044802 -1.34477452

O -4.29733304 -1.32741976 3.22591808

C -4.89813836 -2.06029985 -2.71687151

H -5.15693382 -2.96132684 -3.26754778

H -3.88108038 -1.75040144 -2.97260072

H -5.60813068 -1.26570512 -2.96332437

C -4.62491492 -2.68370697 3.48906470

H -4.54757625 -2.81348089 4.56583005

H -5.64340791 -2.90464671 3.15787664

H -3.92691888 -3.35397006 2.97884905

C -3.56444766 2.17584297 -0.07048513

C -2.12947594 2.45405032 0.38246870

C -1.28100894 1.99695693 -0.81610526

C -2.26875630 2.06993590 -1.99688597

O -3.54716019 2.42977051 -1.46306506

H -4.27764809 2.88037310 0.36889861

H -1.86080023 1.93186439 1.30122921

H -0.91120144 0.98105405 -0.69198003

H -2.33221938 1.10196184 -2.50281050

H -2.00005990 2.83487483 -2.72882669

C -0.09668206 3.00202610 -0.83469276

O -0.37425865 3.97182672 0.16760464

C -1.75977099 3.93331487 0.46236083

H -0.03707051 3.49956549 -1.81099941

H -2.33134981 4.50621104 -0.28068531

H -1.90559906 4.37739596 1.44733896

C 1.21233938 2.30626294 -0.54736809

C 3.45235645 0.75028273 -0.02304273

C 1.86748936 1.66816802 -1.59700547
C 1.68590075 2.21090844 0.75581410
C 2.81688412 1.43489159 1.01334697
C 2.99402353 0.89266391 -1.33413131
H 1.48105642 1.76369761 -2.60385158
H 1.17381570 2.75234451 1.53884927
O 3.70371452 0.21899058 -2.27602930
O 3.36651259 1.25381366 2.24263381
C 2.80619212 1.97278784 3.32445251
H 3.40765705 1.71927712 4.19424212
H 1.76697271 1.67717576 3.49967396
H 2.85125707 3.05026353 3.14363942
C 3.23528810 0.27576187 -3.61172429
H 3.90725305 -0.35313518 -4.19206804
H 3.27406569 1.30025294 -3.99227747
H 2.21518938 -0.11235934 -3.68100701
O 4.53689013 -0.04565056 0.22712140
C 4.26326220 -1.29658939 0.88821598
H 3.56147482 -1.11801287 1.70639283
C 5.59577402 -1.70390854 1.48865624
H 5.49331393 -2.66908250 1.98903595
H 6.33427469 -1.80260948 0.68599549
O 6.00814339 -0.76990348 2.46317501
H 5.86270680 0.10608251 2.08805259
C 3.71814230 -2.32869383 -0.10974069
H 3.76496838 -3.30552070 0.38587475
O 4.58277927 -2.42048519 -1.21836833
H 4.63749649 -1.53013252 -1.59643543
C 2.26824094 -2.08118234 -0.53284441
C -0.37502477 -1.62248624 -1.35232305
C 1.29969410 -1.67062711 0.39355419
C 1.88599374 -2.28436606 -1.85157658
C 0.57209390 -2.05363674 -2.26177843
C -0.00514068 -1.43855362 -0.01021640
H 1.56651391 -1.51290944 1.43021439
H 2.62530280 -2.61896380 -2.56813169
H 0.26747995 -2.21074543 -3.29003945
O -1.02609396 -1.02116703 0.79640598
O -1.65172233 -1.37589485 -1.74727104
H -2.17884011 -1.12731954 -0.97199452
C -0.71131488 -0.72570745 2.14317972
H -1.64128036 -0.40775239 2.60965279
H -0.33163642 -1.61267344 2.65913489
H 0.03357098 0.07707662 2.19168562

β-β' topC_α-C_β

O 1.56313573 4.47419125 -1.46374462
C 2.51478705 4.12974485 -0.55162887
C 4.54572950 3.71443725 1.29172053
C 3.34027649 5.20005645 -0.16133655
C 2.70925205 2.86632103 -0.01949106
C 3.73951613 2.65486004 0.90668622

C 4.34831981 4.98915528 0.75910633
H 5.34031059 3.55335929 2.01199480
H 2.06481798 2.03883341 -0.29605260
H 4.96837820 5.82782026 1.05018881
C 0.73348326 3.43590459 -1.95874806
H 0.03383642 3.90287874 -2.64816128
H 0.18156743 2.95853871 -1.14609498
H 1.32806188 2.68335143 -2.48557439
O 3.13977740 6.43258619 -0.68793409
H 2.40285607 6.37328601 -1.30814185
C 3.98174075 1.25965593 1.42280097
H 4.73963308 1.29878488 2.21730249
C 4.52010216 0.35993966 0.30121545
H 3.80380234 0.33505777 -0.52662363
C 5.88916333 0.80216952 -0.18726067
H 5.84564268 1.85197328 -0.48051589
H 6.59380660 0.70763707 0.65165824
O 6.33745372 0.07041669 -1.30315425
H 6.01692048 -0.83396545 -1.21178869
O 4.68675240 -0.98729857 0.81590401
C 3.70059744 -1.89340630 0.55372599
C 1.66747600 -3.75535072 0.05309474
C 3.47799570 -2.37138103 -0.74586963
C 2.93524597 -2.41525533 1.59456705
C 1.92770804 -3.33928576 1.35253694
C 2.46201759 -3.29244346 -0.99376581
H 1.33361177 -3.68749358 2.19136733
H 2.29019172 -3.66955172 -1.99230398
O 4.30204706 -1.87028180 -1.69501646
O 3.11521901 -1.93579036 2.87104374
C 3.96760741 -2.08402879 -3.05992322
H 4.67742147 -1.48898733 -3.63005210
H 2.94809880 -1.74263866 -3.26002762
H 4.07275993 -3.13921568 -3.32515405
C 4.31320869 -2.39595491 3.50628669
H 4.33211668 -1.93854668 4.49351333
H 4.28370978 -3.48404096 3.60275676
H 5.18935914 -2.09097045 2.93195050
C 0.48294763 -4.66528935 -0.22136691
C -0.84829639 -3.93566541 0.08853029
C -1.11239547 -3.30781672 -1.24427734
C -0.37645632 -4.06263629 -2.29546729
O 0.39595860 -5.03761065 -1.58772812
H 0.58935840 -5.59094078 0.35200332
H -0.72195559 -3.18143517 0.87159525
H -1.74703382 -2.45364631 -1.42956012
H 0.26901079 -3.40196244 -2.89081435
H -1.03014022 -4.60454656 -2.98984884
C -3.75737934 -3.56282573 0.05937657
O -3.09395999 -4.23996681 1.02419667
C -1.93134606 -4.92273578 0.55637169
H -3.87840072 -4.06448206 -0.89692669
H -2.20044070 -5.61463745 -0.25183731
H -1.57503942 -5.49875599 1.41140360

C -4.08935240 -2.21006773 0.23140363
C -4.65852629 0.51747690 0.48539394
C -4.56738108 -1.47630248 -0.88155677
C -3.89318412 -1.55946040 1.47273019
C -4.19966871 -0.21379079 1.59227796
C -4.82634540 -0.12469382 -0.75038634
H -4.69027656 -1.98093757 -1.83060649
H -3.52864186 -2.13761793 2.30974229
O -5.22577662 0.68258385 -1.77028193
O -4.06129751 0.50942410 2.73318413
C -3.73786876 -0.18984828 3.92023979
H -3.74533154 0.55252838 4.71473386
H -2.74484159 -0.64486232 3.85556160
H -4.47995940 -0.96535369 4.12927327
C -5.19171137 0.14330258 -3.08000394
H -5.44052247 0.96439161 -3.74929169
H -5.93085954 -0.65435569 -3.19336574
H -4.19120729 -0.23379048 -3.31281974
O -4.97242601 1.84186059 0.58994879
O 2.76517978 0.74910909 1.93364673
H 2.94649130 -0.08194427 2.39481726
C -3.88455474 2.76879743 0.76891608
H -3.22924114 2.40028249 1.56045121
C -4.55338692 4.04097763 1.25814760
H -3.79656493 4.81071007 1.42158467
H -5.25300975 4.39432299 0.49328532
O -5.19961038 3.82405945 2.49328524
H -5.72565232 3.02311566 2.39447735
C -3.13051995 2.97614474 -0.54922512
H -2.49254216 3.85817790 -0.40721021
O -4.03144206 3.32207536 -1.57563785
H -4.68047723 2.60394757 -1.62503976
C -2.20843096 1.82440696 -0.95547967
C -0.28053118 -0.04827201 -1.76446829
C -1.47754325 1.10103590 -0.00242211
C -1.98692760 1.56827472 -2.30284666
C -1.02477642 0.63844069 -2.70658675
C -0.53662568 0.16108617 -0.39818259
H -1.63430343 1.27125309 1.05402754
H -2.54609624 2.12687635 -3.04329270
H -0.82549993 0.45630982 -3.75643026
O 0.20828371 -0.61332688 0.43620764
O 0.69521375 -0.91459943 -2.14622075
H 1.03977292 -1.35358307 -1.35232231
C 0.00106099 -0.43953664 1.82923213
H 0.66455510 -1.14358804 2.32643659
H 0.26773114 0.57495524 2.13317928
H -1.04201931 -0.66284150 2.08055916

β-β′ topC$_{\alpha}$-O

O 1.71932121 4.82809496 -0.80010033
C 2.79903627 4.23613729 -0.21549378

C	5.13177348	3.29902587	0.95390260
C	3.90786586	5.08970261	-0.07778332
C	2.86537036	2.92359446	0.22366171
C	4.04634192	2.44841945	0.80692388
C	5.06678109	4.61948383	0.50952152
H	6.04381353	2.93494399	1.41457232
H	2.01265140	2.25888743	0.13764027
H	5.90452281	5.29682052	0.61891298
C	0.52567924	4.06713115	-0.86324652
H	-0.22068163	4.69671102	-1.34372712
H	0.19491863	3.79702519	0.14332911
H	0.66727918	3.15622087	-1.45170683
O	3.83080769	6.37141859	-0.51144607
H	2.94674895	6.50860086	-0.87320710
C	4.13808832	0.99803954	1.20752018
H	5.01049793	0.86447318	1.86194615
C	4.34523214	0.09818354	-0.02028769
H	3.47495180	0.16768467	-0.68331308
C	5.63005010	0.42702911	-0.76538110
H	5.60739259	1.47252747	-1.07406997
H	6.46878827	0.29442012	-0.06584721
O	5.81739097	-0.35015273	-1.92253299
H	5.44282820	-1.22270886	-1.75828875
O	4.48011312	-1.27318992	0.44195022
C	3.40551783	-2.10804230	0.35193842
C	1.26886303	-3.92077042	0.20871199
C	2.97148128	-2.60338033	-0.88885659

C	2.77866580	-2.57138337	1.50593966
C	1.72043865	-3.47081530	1.44176556
C	1.90433735	-3.49582482	-0.95793204
H	1.25485418	-3.78773344	2.36958840
H	1.57840634	-3.89176595	-1.90985188
O	3.66392339	-2.15426357	-1.95995019
O	3.16724492	-2.07787375	2.72976120
C	3.19560753	-2.48025602	-3.26206223
H	3.85856930	-1.95638130	-3.94674460
H	2.17002805	-2.12823900	-3.39876272
H	3.25766163	-3.55761353	-3.43644163
C	4.40921284	-2.60866216	3.20668297
H	4.59709856	-2.13357594	4.16748876
H	4.32062234	-3.68975536	3.33878290
H	5.21392875	-2.37795356	2.50674878
C	0.06849350	-4.84961161	0.13515344
C	-1.24580184	-4.11827803	0.47203200
C	-1.52023694	-3.44355473	-0.89223004
C	-1.05195310	-4.53036240	-1.87242298
O	-0.12376921	-5.35766612	-1.17433270
H	0.23739836	-5.70566329	0.79587836
H	-1.08873542	-3.38754162	1.26872280
H	-0.82446437	-2.59521497	-0.94870956
H	-0.57657580	-4.09571154	-2.75703851
H	-1.88944371	-5.15551276	-2.19948996
C	-2.87018228	-2.92407071	-1.26799384
O	-3.38496809	-4.50140897	1.57937903

C -2.35245073 -5.08525100 0.89485538
H -3.21679320 -3.19435636 -2.26154230
H -2.74354427 -5.67448628 0.04981419
H -1.95531708 -5.83908687 1.60313305
C -3.48365316 -1.76966784 -0.68497858
C -4.44477439 0.67557382 0.28934024
C -4.17538488 -0.88493860 -1.53733385
C -3.35061733 -1.44628588 0.67754008
C -3.86165917 -0.25042281 1.16165099
C -4.61123539 0.33869233 -1.06008647
H -4.26524987 -1.13578319 -2.58521478
H -2.89144360 -2.15393562 1.35071318
O -5.15480692 1.31588300 -1.83741351
O -3.77122780 0.13640999 2.46260129
C -3.58868534 -0.88725016 3.42825675
H -3.74901485 -0.41708537 4.39579589
H -2.57679368 -1.30172507 3.39184454
H -4.31019328 -1.69424835 3.27612772
C -5.22967835 1.07396167 -3.23165165
H -5.64365933 1.97886509 -3.67114015
H -5.89035229 0.22950465 -3.44530889
H -4.23452260 0.88250319 -3.64437904
O -4.84516088 1.91234272 0.70764860
O 2.95421146 0.63823746 1.89144666
H 3.08330127 -0.22965165 2.29961004
C -3.80431136 2.82990403 1.09913939
H -3.18012484 2.34914406 1.85539670
C -4.54922463 3.98161408 1.74999095
H -3.83221621 4.72881397 2.09591777
H -5.20884433 4.44685546 1.01010089
O -5.26724129 3.54007591 2.88094164
H -5.78319675 2.77624267 2.60177027
C -2.97415076 3.28333442 -0.11382517
H -2.35661075 4.12163564 0.23411149
O -3.81371888 3.81555069 -1.11090335
H -4.46637913 3.13145985 -1.32552688
C -2.03698750 2.20503310 -0.66201538
C -0.31702388 0.22430797 -1.65451926
C -1.29762281 1.40132404 0.21577633
C -1.88370002 2.01851671 -2.02918961
C -1.02217537 1.03416648 -2.52498990
C -0.46765637 0.40505399 -0.26900364
H -1.39736775 1.52939687 1.28550179
H -2.43582457 2.65028378 -2.71407293
H -0.89162572 0.88750046 -3.59071520
O 0.23208734 -0.47982676 0.49462651
O 0.51286926 -0.75220346 -2.11513065
H 0.92612089 -1.17847092 -1.34830074
C 0.11510470 -0.33258089 1.90155637
H 0.70968950 -1.12803085 2.34400559
H 0.51487260 0.63268161 2.21974042
H -0.93323442 -0.43331577 2.20038241

β-β′ topC_{β}-C_{γ}

O 1.62233517 4.89610993 -0.80642524

C 2.69044545 4.32753982 -0.17889207

C 4.99623926 3.43886100 1.07849211

C 3.77085283 5.20746422 0.01086080

C 2.77086871 3.01403795 0.25482542

C 3.93905565 2.56337714 0.88173309

C 4.91589610 4.76140843 0.64227480

H 5.89828725 3.09306433 1.57156141

H 1.93948978 2.32870708 0.12972781

H 5.73155016 5.45814735 0.79002138

C 0.46281013 4.09474050 -0.94841332

H -0.27638580 4.70270052 -1.46658531

H 0.08310040 3.80043383 0.03375329

H 0.67311629 3.19657471 -1.53573987

O 3.67997899 6.49001954 -0.41751324

H 2.81041343 6.60741392 -0.81911853

C 4.05234189 1.11121065 1.26860385

H 4.92561123 0.98615066 1.92371282

C 4.28485515 0.22872235 0.03202067

H 3.40281726 0.25619533 -0.61900285

C 5.54043886 0.62642090 -0.73013302

H 5.45479258 1.66730080 -1.04314545

H 6.39404870 0.54624532 -0.04052254

O 5.75658162 -0.14479724 -1.88580279

H 5.44139074 -1.03859653 -1.71084971

O 4.49732965 -1.13740464 0.48288575

C 3.47108399 -2.03080908 0.39956857

C 1.43860755 -3.96760176 0.27040268

C 3.08589401 -2.58479344 -0.83230861

C 2.85362873 -2.50188572 1.55562021

C 1.84841653 -3.45837458 1.49734498

C 2.07074515 -3.53938591 -0.89615355

H 1.39798990 -3.78358300 2.42901566

H 1.79073514 -3.96411757 -1.84946648

O 3.76651627 -2.12577414 -1.90561919

O 3.20089578 -1.97064867 2.77552146

C 3.31976924 -2.48800104 -3.20618656

H 3.95276481 -1.93185349 -3.89373641

H 2.27610410 -2.19602628 -3.34470683

H 3.44459558 -3.56122944 -3.37243879

C 4.46879073 -2.41895333 3.26995249

H 4.61791763 -1.92105429 4.22595828

H 4.44417837 -3.50162790 3.41550471

H 5.26439028 -2.14859624 2.57395978

C 0.29247132 -4.96687761 0.25146666

C -1.01533817 -4.28631571 0.70595630

C -1.46564745 -3.10949237 -0.09305741

C -0.42007025 -5.12968523 -2.05116485

O 0.20322183 -5.67745867 -0.97766411

H 0.52278749 -5.75405279 0.97485720

H -0.82766626 -3.97372141 1.74374013

H -0.85893741 -2.26883724 -0.39081909

H	-0.56219234	-4.05746170	-2.09998937
H	-0.31464268	-5.73097410	-2.94263091
C	-2.92554716	-3.25796264	-0.39413509
O	-3.35957777	-4.26132396	0.51955035
C	-2.27830844	-5.17032183	0.67425963
H	-3.08480177	-3.61845268	-1.42552463
H	-2.23865791	-5.86206353	-0.17541312
H	-2.44209655	-5.73264006	1.59297469
C	-3.65595328	-1.95385318	-0.20012997
C	-4.59159506	0.62934963	0.14604595
C	-3.95432223	-1.18142262	-1.31483641
C	-3.87616501	-1.47897167	1.08987406
C	-4.35796870	-0.18442371	1.25969925
C	-4.43068208	0.11821080	-1.13937310
H	-3.78034718	-1.58282340	-2.30439204
H	-3.65672856	-2.12458147	1.92985144
O	-4.72754700	0.97362557	-2.15251598
O	-4.59713408	0.40608306	2.45818332
C	-4.38008204	-0.36615046	3.62365155
H	-4.64677550	0.27504163	4.46036712
H	-3.32975969	-0.66212785	3.70913001
H	-5.01153402	-1.25890991	3.62451974
C	-4.54650425	0.50490435	-3.47726126
H	-4.81997710	1.33274774	-4.12815461
H	-5.19932959	-0.34904722	-3.67780784
H	-3.50316105	0.22761494	-3.65219354
O	-4.97483764	1.93255450	0.31012215
O	2.87295813	0.72605836	1.94663325
H	3.01833276	-0.14146486	2.34941436
C	-3.93695691	2.81791884	0.77295128
H	-3.38638399	2.32548407	1.57864972
C	-4.68223282	4.00188230	1.36050363
H	-3.96708465	4.74860605	1.71194887
H	-5.30548993	4.45386755	0.58136541
O	-5.44969253	3.60189927	2.47454949
H	-5.89803240	2.78512868	2.22708948
C	-3.00540127	3.21336098	-0.38266009
H	-2.41494436	4.06731928	-0.02640039
O	-3.76248812	3.69885288	-1.46566826
H	-4.38336311	2.99431375	-1.70470116
C	-2.02399343	2.11852947	-0.81122594
C	-0.16971267	0.18089441	-1.63240173
C	-1.32492833	1.35951676	0.13976532
C	-1.76532774	1.90472121	-2.15817969
C	-0.84614837	0.93628612	-2.57062226
C	-0.40787529	0.40147479	-0.26417798
H	-1.49425471	1.51431975	1.19783714
H	-2.28547101	2.50343275	-2.89514890
H	-0.64117666	0.76604549	-3.62119683
O	0.32143858	-0.40558618	0.55406780
O	0.71803045	-0.77798341	-2.01031809
H	1.09287322	-1.17368963	-1.20850231
C	0.05462724	-0.33485872	1.94426827
H	0.69510089	-1.07759571	2.41496595

H 0.30919381 0.65359326 2.33344859

H -0.99892752 -0.57328223 2.13194707

β-β’ topC$_{\gamma}$-O

O 1.80691532 4.85730698 -0.77021010
C 2.85779065 4.23704549 -0.16270064
C 5.13439256 3.23649928 1.06359219
C 3.97444353 5.06924958 0.03101584
C 2.88827697 2.91490105 0.25043542
C 4.04041469 2.40825987 0.86336271
C 5.10532030 4.56714392 0.64588395
H 6.02457651 2.84730665 1.54578957
H 2.02907879 2.26586976 0.12209886
H 5.95011275 5.22759373 0.79657577
C 0.62772155 4.09096076 -0.94185752
H -0.09578166 4.73429015 -1.43918115
H 0.23767147 3.77087725 0.02808661
H 0.81915570 3.20932329 -1.55925855
O 3.93269065 6.36109800 -0.37701075
H 3.06625896 6.51965839 -0.77109251
C 4.09094291 0.94991426 1.23972434
H 4.95416643 0.78422709 1.89902951
C 4.29111199 0.06068372 0.00269646
H 3.40637666 0.11199866 -0.64343702
C 5.55385160 0.41915446 -0.76679907
H 5.49750607 1.46180106 -1.08074092
H 6.40871368 0.31459522 -0.08193983
O 5.74040002 -0.35991063 -1.92232911

H 5.39274014 -1.24165947 -1.74724064
O 4.46638049 -1.31009363 0.45725397
C 3.41316553 -2.17262222 0.38495606
C 1.32162751 -4.03264186 0.27358137
C 3.00670388 -2.71923616 -0.84561213
C 2.78267410 -2.61052542 1.54664355
C 1.74270496 -3.53162272 1.49719657
C 1.96072978 -3.63753364 -0.89989940
H 1.27250226 -3.83030963 2.42878494
H 1.63879697 -4.05911253 -1.84255693
O 3.69358727 -2.28241880 -1.92319791
O 3.14869650 -2.07900470 2.76147044
C 3.21153636 -2.62126452 -3.21777098
H 3.84574567 -2.07734178 -3.91392335
H 2.17324624 -2.30029237 -3.33107789
H 3.30258619 -3.69622633 -3.39370988
C 4.40477772 -2.55855090 3.25652851
H 4.57130455 -2.05596812 4.20723621
H 4.34958314 -3.63863218 3.41266184
H 5.20553577 -2.31801011 2.55558027
C 0.13353123 -4.97668463 0.22318121
C -1.19316913 -4.21723288 0.46189048
C -1.60917982 -3.19338969 -0.62376712
C -1.13649559 -3.46319984 -2.00785176

O	0.07512871	-5.78412768	-0.87726413
H	0.21324297	-5.69142048	1.06783872
H	-1.06734392	-3.67120051	1.40186882
H	-1.28471294	-2.19455863	-0.32559513
H	-1.09135763	-2.65100897	-2.72146432
H	-1.05069205	-4.48073826	-2.37569124
C	-3.19005901	-3.25492627	-0.52226671
O	-3.49341883	-4.14737765	0.53581355
C	-2.43738385	-5.08798790	0.59462616
H	-3.58386594	-3.66327660	-1.46148258
H	-2.50332426	-5.80650717	-0.23244124
H	-2.50890003	-5.62122094	1.54321138
C	-3.77327556	-1.89301190	-0.27248640
C	-4.52974129	0.74332521	0.14559540
C	-4.01471214	-1.06747538	-1.36643577
C	-3.94413254	-1.43250181	1.02898045
C	-4.33643645	-0.11027457	1.23398951
C	-4.39526735	0.25624167	-1.15461800
H	-3.88131758	-1.45729993	-2.36736309
H	-3.78091532	-2.11423234	1.85200786
O	-4.63532805	1.15637349	-2.14340866
O	-4.53184410	0.45936791	2.45062034
C	-4.39422451	-0.36522078	3.59204844
H	-4.62011473	0.26850542	4.44633035
H	-3.37301658	-0.74879721	3.68065583
H	-5.09569832	-1.20326306	3.55622496
C	-4.45058388	0.72318239	-3.47964753
H	-4.65438786	1.58883587	-4.10651389
H	-5.15042012	-0.07972456	-3.72685356
H	-3.42225015	0.38603754	-3.63885464
O	-4.86720847	2.05526128	0.33936473
O	2.89181030	0.61170482	1.90966994
H	3.00419447	-0.25727884	2.31995265
C	-3.81550341	2.90282322	0.83814320
H	-3.29729558	2.38409419	1.64831577
C	-4.53977064	4.09982674	1.42576679
H	-3.81141240	4.81805718	1.80800613
H	-5.12801896	4.58365829	0.63867950
O	-5.34848034	3.70694551	2.51292113
H	-5.82470743	2.91625873	2.23535000
C	-2.84957271	3.28765179	-0.29174576
H	-2.23311556	4.11112665	0.09113990
O	-3.56832062	3.82207388	-1.37830189
H	-4.20890523	3.14491224	-1.64361718
C	-1.90900886	2.16039527	-0.72417721
C	-0.13907981	0.14630315	-1.54535261
C	-1.27174093	1.34697575	0.22533256
C	-1.63417816	1.95993120	-2.06934999
C	-0.75453297	0.95536019	-2.48137583
C	-0.40371842	0.34475182	-0.17798869
H	-1.45797641	1.48945813	1.28205310
H	-2.10768903	2.59846080	-2.80443631
H	-0.52955873	0.79923561	-3.53020465
O	0.24117465	-0.53192419	0.63989767

O 0.71897705 -0.83340546 -1.92851048
H 0.98443408 -1.33365365 -1.14124040
C 0.04799556 -0.38156765 2.03575761
H 0.65008756 -1.15389983 2.51025890
H 0.39773415 0.59913832 2.36622170
H -1.00953544 -0.52329993 2.28637404

β-β' topC_{α}-C_{Ph} α-side

O -4.47503800 3.38868037 0.38716562
C -4.54691256 2.09088474 -0.03433910
C -4.98145288 -0.50737995 -0.89696218
C -5.85803945 1.59573617 -0.13118476
C -3.46803734 1.28644054 -0.35816102
C -3.68483085 -0.02681115 -0.79022482
C -6.06900284 0.29992554 -0.56404127
H -5.15491635 -1.52088192 -1.24270570
H -2.45309805 1.66041055 -0.31520991
H -7.08736784 -0.05920058 -0.64495001
C -3.18973500 3.97053064 0.48357442
H -3.34289623 4.99431532 0.81669834
H -2.68915218 3.96939262 -0.48870541
H -2.57480672 3.43465155 1.21244329
O -6.90947284 2.39091985 0.18638326
H -6.56247650 3.25043328 0.45353479
C -2.49126209 -0.90025449 -1.09176607
H -2.81616993 -1.74989143 -1.70825205
C -1.88520889 -1.46905000 0.20081294
H -1.53534205 -0.64642186 0.83390085
C -2.84897828 -2.36150799 0.96569314
H -3.75733232 -1.80315094 1.19381795
H -3.11778227 -3.20737785 0.31595246
O -2.31722078 -2.81766288 2.18667993
H -1.36913463 -2.94372562 2.06599498
O -0.75515780 -2.30952763 -0.14712960
C 0.49890393 -1.77367143 -0.07375789
C 3.10592142 -0.75008201 0.05139896
C 1.10179349 -1.50820744 1.16489993
C 1.24006472 -1.56239092 -1.23248918
C 2.53157105 -1.05087166 -1.17760511
C 2.39206999 -0.99328199 1.22530391
H 3.05577302 -0.89606495 -2.11337158
H 2.86991896 -0.79236919 2.17461798
O 0.33699979 -1.79786853 2.24632619
O 0.66126444 -1.80457827 -2.45590129
C 0.83215095 -1.46472561 3.53148831
H 0.04068614 -1.72874502 4.22915463
H 1.04283294 -0.39389567 3.59944895
H 1.73669267 -2.03391850 3.76156101
C 0.53933287 -3.19308778 -2.77769064
H 0.06985205 -3.24102232 -3.75831199
H 1.53125723 -3.65017115 -2.82058898
H -0.07929584 -3.70365857 -2.03765894

C 4.53366563 -0.22739492 0.13740930

C 4.82091524 0.95634093 -0.79644784

C 4.52112779 2.20218764 0.07968787

C 4.34664457 1.60665367 1.49541941

O 4.84498502 0.27400206 1.42563061

H 5.22171978 -1.05925491 -0.04296739

H 4.22371972 0.94378125 -1.70605172

H 3.60629063 2.71490052 -0.23001433

H 3.28771781 1.59666657 1.77863682

H 4.92482302 2.13466744 2.25293690

C 5.74328896 3.04865833 -0.12219991

O 6.58803275 2.50822769 -1.04202683

C 6.31615820 1.11048273 -1.10879882

H 5.77287716 4.12817696 -0.07265156

H 6.91799508 0.59901895 -0.34986112

H 6.59972582 0.76348802 -2.10127185

O -1.53599660 -0.12297921 -1.78303887

H -0.87017039 -0.70860163 -2.16895610

β-β’ topC_{α}-C_{Ph} phenyl-side

C 0.74042642 1.59654433 2.31488508

C -1.10640396 0.06233575 1.07980685

C 0.91149546 0.24043834 2.41559594

C -0.27846753 2.26366122 1.68366266

C -1.23241675 1.45455087 1.04920272

C -0.06236159 -0.54647955 1.78413312

H 1.75064810 -0.19379619 2.94144674

H -0.34627454 3.34341436 1.68463667

O -0.07065009 -1.90119209 1.78091625

O -2.30375325 1.91920047 0.35784215

C -2.52124908 3.31688094 0.34239457

H -3.43173670 3.47098804 -0.23169419

H -1.69125378 3.84031151 -0.14256301

H -2.65424747 3.70303526 1.35696068

C 0.99502851 -2.56214221 2.44297486

H 0.82342828 -3.62651283 2.29757770

H 0.98562501 -2.33233872 3.51197034

H 1.95591564 -2.28039962 2.00449352

O -2.01986302 -0.72329966 0.43092574

C -1.93726444 -0.73981675 -1.00933343

H -1.78192522 0.28055631 -1.36741763

C -3.31344792 -1.19245609 -1.46019022

H -3.33493147 -1.27147935 -2.54905240

H -3.52303209 -2.17911750 -1.03402393

O -4.29416860 -0.24679110 -1.09044688

H -4.11447510 -0.00362135 -0.17520268

C -0.81684245 -1.67953748 -1.47604193

H -0.95261820 -1.82819505 -2.55366853

O -0.99209189 -2.94977193 -0.88984945

H -0.97315988 -2.81162681 0.06866410

C 0.59153596 -1.13253479 -1.23449547

C 3.16286605 -0.16361011 -0.67366462

C 0.89055334 0.22648095 -1.40866199
C 1.60298859 -1.99038037 -0.82612453
C 2.88368486 -1.51071817 -0.54742310
C 2.15622983 0.70707565 -1.11966765
H 0.12692683 0.91714432 -1.74123578
H 1.38358704 -3.04380392 -0.70700695
H 3.67691812 -2.17477168 -0.22428158
O 2.55516941 2.01062635 -1.21844514
O 4.39567115 0.31609592 -0.37485829
H 4.38380128 1.27087539 -0.51263422
C 1.58476599 2.96172654 -1.60551738
H 2.08230870 3.92880407 -1.59146498
H 1.21627658 2.75456282 -2.61471763
H 0.74898153 2.96491281 -0.89867423

β-O-4 topC_{α}-C_{β} α-side

C 2.57185567 0.97479184 0.02164059
H 2.57367582 2.05346475 0.08309824
O 3.81663122 0.44981939 -0.03520210
H 3.77206960 -0.48811304 -0.24321069
C 1.38719464 0.21881566 0.02443258
C -1.03938340 -1.21890474 -0.00343642
C 0.13525051 0.90024926 0.01454481
C 1.36862051 -1.19578581 0.03403726
C 0.16957879 -1.89374010 0.01459435
C -1.04333880 0.19319436 0.00092235
H 0.13182390 1.98286180 0.01054519
H 2.28889527 -1.76741473 0.08087933
H 0.15274840 -2.97669464 0.02389836
O -2.29754147 0.73303969 -0.01143351
O -2.20935140 -1.90422017 -0.01987387
H -2.93019777 -1.26319379 -0.02581267
C -2.40373900 2.14261765 -0.00470817
H -3.46730103 2.36870422 -0.01254233
H -1.94543812 2.56200199 0.89557330
H -1.93041636 2.57184340 -0.89251702

β-O-4 topC_{α}-C_{β} β-side

O 4.23009943 1.58104703 -2.01006967
C 4.36158618 0.58621459 -1.06541920
C 4.78459344 -1.42773189 0.77729023
C 5.65731453 0.10559508 -0.84802663
C 3.28103234 0.04767659 -0.38322590
C 3.48698559 -0.97827558 0.54028985
C 5.86827914 -0.89999366 0.08570744
H 4.95334075 -2.21417020 1.50596609
H 2.28854154 0.44769354 -0.55999561
H 6.87775772 -1.25151119 0.25857751
C 4.04691233 2.88845752 -1.44909848
H 4.00104568 3.57899379 -2.28975987
H 4.89477782 3.14013182 -0.80381281
H 3.11522358 2.93506135 -0.88712338

O	6.70111470	0.62806488	-1.53660630
H	6.33206834	1.23117175	-2.19488104
C	2.31255955	-1.63754008	1.22348323
H	2.66494942	-2.12388125	2.14337023
C	1.69360191	-2.73325591	0.33868621
H	1.22345379	-2.26753997	-0.53521765
C	2.68842613	-3.79820931	-0.09670891
H	3.50457208	-3.33181755	-0.64827243
H	3.10724743	-4.26762811	0.80588044
O	2.10858333	-4.76279215	-0.94162245
H	1.19785552	-4.89779273	-0.65510491
O	0.67717563	-3.43605915	1.10175904
C	-0.62060936	-3.03741368	0.94544704
C	-3.23721439	-2.11811857	0.59410043
C	-1.33534671	-3.35571833	-0.21791260
C	-1.27419365	-2.34688746	1.96329128
C	-2.57429035	-1.89014780	1.79406921
C	-2.63438866	-2.88702344	-0.39832136
H	-3.01785546	-1.29534077	2.58542326
H	-3.18406359	-3.09178350	-1.30732076
O	-0.65273461	-4.10028642	-1.12231955
O	-0.58739464	-2.01220428	3.10554132
C	-1.24202361	-4.32672372	-2.38980035
H	-0.50295026	-4.88007765	-2.96459814
H	-1.46233581	-3.37830297	-2.88903225
H	-2.15843069	-4.91552290	-2.29625588
C	-0.30673111	-3.11649339	3.97130082
H	0.23632511	-2.70839814	4.82144415
H	-1.24387236	-3.56341609	4.31220066
H	0.30161644	-3.86143975	3.45611754
C	-4.51554779	-1.34801627	0.30152080
C	-4.12126013	0.11472395	0.08297940
C	-3.70798784	0.10018818	-1.39312648
C	-4.74152373	-0.86481342	-1.98166994
O	-5.12704687	-1.74671916	-0.91811066
H	-5.24664316	-1.48527968	1.10300242
H	-3.30644050	0.42660231	0.73430332
H	-2.69328918	-0.26858919	-1.53054533
H	-4.33705929	-1.46020123	-2.80390130
H	-5.63027030	-0.33286198	-2.33565001
C	-3.84853394	1.57801765	-1.76057323
O	-4.99477920	2.01821192	-1.04501989
C	-5.25270674	1.14828030	0.06596754
H	-4.03918657	1.74437228	-2.82465022
H	-6.22829049	0.67468113	-0.08252828
H	-5.28528101	1.74270863	0.98213670
C	-2.57348054	2.28513337	-1.32211745
C	-0.16256465	3.18295642	-0.28733484
C	-1.38043451	1.94939320	-1.96096495
C	-2.57705707	3.10676364	-0.20300655
C	-1.36038268	3.55474078	0.31855780
C	-0.16728707	2.38900037	-1.43623993
H	-1.40042010	1.30544690	-2.83137421
H	-3.52099978	3.36346544	0.25692378

O 1.05196261 2.07363007 -1.92998401
O -1.23517651 4.32312346 1.43058122
C -2.41384575 4.62735176 2.15530374
H -2.09319092 5.20455245 3.01950346
H -2.91078769 3.71260149 2.49100005
H -3.10249913 5.22276291 1.55011358
C 1.11513892 1.17228226 -3.02223800
H 2.17538876 1.03062947 -3.22045896
H 0.61977065 1.59033152 -3.90335257
H 0.65526760 0.21372151 -2.75951841
O 1.02155187 3.59150360 0.26939730
O 1.34698359 -0.65007847 1.53109310
H 0.75308488 -0.99991422 2.20974222
C 1.57230531 2.72015475 1.18997203
H 1.09605841 1.75136825 1.29348757
C 2.23963439 3.42598582 2.30780042
H 2.78811825 2.71188361 2.92220429
H 2.94634812 4.16428301 1.90793871
O 1.29172676 4.07510733 3.15790521
H 0.60876885 4.44329416 2.58376462

β-O-4 topC_{α}-C_{Ph} α-side

O -1.85880970 0.60694989 -1.77639271
C -1.25581692 1.74565385 -1.29500307
C -0.14908726 4.11432232 -0.37542237
C -1.81852405 2.97015327 -1.68282066
C -0.15771950 1.71310529 -0.45263254
C 0.41323736 2.90280144 0.00943045
C -1.25926500 4.15195667 -1.21494022
H 0.27326505 5.04474569 -0.00992308
H 0.24708168 0.75527254 -0.13722122
H -1.71128305 5.09035962 -1.51192296
C -1.03558083 -0.15414922 -2.64855146
H -1.64752751 -0.97438218 -3.02030738
H -0.17588624 -0.56782960 -2.11237915
H -0.68626553 0.46343501 -3.48213503
O -2.89757491 3.01558264 -2.49686107
H -3.35322443 2.15979318 -2.46558753
C 1.65887819 2.83448406 0.86416647
H 1.78028840 3.78142432 1.40921095
C 2.88453178 2.62966684 -0.05685693
H 2.60490717 1.95798100 -0.87179143
C 3.39322738 3.93994820 -0.62367507
H 2.55737388 4.49704643 -1.05222298
H 3.81310030 4.52742054 0.20490353
O 4.34425070 3.75352029 -1.64479743
H 4.87476907 2.98324829 -1.41043618
O 4.00036050 2.03732173 0.65851104
C 4.05012564 0.67079161 0.57317480
C 3.71036025 -2.08796694 0.34249354
C 4.25838160 0.03593568 -0.65845607
C 3.85669394 -0.11372232 1.70706560

C 3.68093249 -1.48699270 1.59650187

C 4.06649334 -1.33694823 -0.77620030

H 3.44380865 -2.04530725 2.49531883

H 4.15673601 -1.83384338 -1.73206012

O 4.58452692 0.86060347 -1.68490194

O 3.70838631 0.49283035 2.92996076

C 4.54290978 0.34110153 -3.00284307

H 4.73567739 1.18576998 -3.66021670

H 3.55549488 -0.07709190 -3.22096176

H 5.30896091 -0.42516775 -3.14683119

C 4.90921600 1.08835643 3.43107777

H 4.65543566 1.52408348 4.39532788

H 5.67579616 0.32087802 3.56239888

H 5.26522201 1.86407202 2.75055154

C 3.17853474 -3.50352237 0.15722679

C 1.71010614 -3.52383685 0.59007652

C 1.01259323 -2.90959156 -0.63312077

C 1.90812891 -3.40553115 -1.78716675

O 3.13577954 -3.86756806 -1.21348144

H 3.79662644 -4.24265585 0.67313473

H 1.53297530 -2.95983377 1.50470569

H 0.99660647 -1.82049954 -0.59001397

H 2.11382186 -2.60797929 -2.50755162

H 1.45981072 -4.24740288 -2.32085465

C -0.40381506 -3.51153861 -0.60305862

O -0.33775634 -4.60527264 0.32320154

C 1.01669924 -4.88202063 0.65119957

H -0.66937036 -3.91141396 -1.58943289

H 1.46191200 -5.57718847 -0.07253945

H 1.03123166 -5.33876735 1.64072880

C -1.49015015 -2.55000736 -0.17307171

C -3.56258706 -0.88990390 0.68732112

C -2.69342353 -2.53249205 -0.86737671

C -1.30117057 -1.71248463 0.92453963

C -2.33883278 -0.89280742 1.36773857

C -3.71714429 -1.68394258 -0.44784545

H -2.82418585 -3.18248497 -1.72270059

H -0.35944998 -1.72387656 1.45717686

O -4.91671646 -1.55223003 -1.08770803

O -2.27222086 -0.08920548 2.45045354

C -1.02916317 0.00390631 3.13078905

H -1.18732345 0.71846631 3.93513097

H -0.24416929 0.37514657 2.46751250

H -0.74928714 -0.96465335 3.55655142

C -5.12458380 -2.32242755 -2.25737622

H -6.11940110 -2.06521617 -2.61357797

H -5.08271477 -3.39108514 -2.03158360

H -4.38485168 -2.07583331 -3.02507711

O -4.65378786 -0.20136113 1.13486577

O 1.49881113 1.76744025 1.77147012

H 2.30219945 1.65009906 2.29698456

C -4.60734811 1.23855770 1.22015677

H -3.84204932 1.54282638 1.93773211

C -5.98928420 1.59601815 1.76658194

H -6.04869625 2.67566853 1.91150941
H -6.74596406 1.29865605 1.02939423
O -6.22409787 0.99114805 3.01743977
H -6.01634532 0.05619180 2.91684405
C -4.32241716 1.84083925 -0.10934324
H -4.09367386 2.89571622 -0.18256868
O -4.93171034 1.33649600 -1.21734426
H -5.10777708 0.38789373 -1.09322024

β-O-4 topC_{α}-C_{Ph} phenyl-side

C -1.27967380 -1.71939174 0.00002217
C -0.56516764 0.90282986 -0.00003431
C 0.07043354 -1.44050898 -0.00012724
C -2.28585483 -0.79249302 0.00008199
C -1.90522419 0.55644688 0.00000071
C 0.42685838 -0.09391666 -0.00007517
H 0.81442954 -2.22683276 -0.00025732
H -3.33204892 -1.07152080 0.00023003
H -2.64251411 1.35037734 0.00000792
O 1.70204737 0.39152170 0.00004673
O -0.20603160 2.21240946 -0.00002143
H 0.75721473 2.25889581 -0.00003109
C 2.75833254 -0.54885319 0.00006905
H 3.68028774 0.02748993 0.00002488
H 2.71814233 -1.17721963 0.89443211
H 2.71813849 -1.17731813 -0.89423201

β-O-4 topC_{β}-O β-side

C -2.30980850 0.81872639 0.20553722
H -1.87259652 1.14328439 1.14491596
C -3.71341711 1.21040910 -0.12213855
H -3.93458790 2.21624246 0.23570496
H -3.85915746 1.19502950 -1.21038437
O -4.66210110 0.35278729 0.51053646
H -4.42688969 -0.54943535 0.26510004
C -1.73570501 -0.38492055 -0.47336052
H -1.92713081 -0.30909190 -1.55050403
O -2.48670089 -1.55286126 -0.09125734
H -2.27443942 -1.74252966 0.83056666
C -0.24780296 -0.57151195 -0.24413912
C 2.50887102 -0.89658882 0.10219522
C 0.59022723 0.55028272 -0.18565383
C 0.30761319 -1.83955547 -0.13815369
C 1.68171761 -2.00150001 0.03934484
C 1.95530214 0.38887037 -0.01692759
H 0.16270010 1.54137381 -0.27747237
H -0.32965051 -2.71205243 -0.20786187
H 2.12656584 -2.98499085 0.12688091
O 2.87399024 1.39607811 0.05660235
O 3.84393394 -1.04858101 0.27600352

H 4.24656291 -0.17206281 0.28829450

C 2.40284837 2.72395448 -0.06374520

H 3.27721928 3.36564610 0.01298212

H 1.91803178 2.87806728 -1.03200800

H 1.70131899 2.96213687 0.74095304

β-O-4 topC_{β}-O_{O}-side

O 4.95699159 2.62062819 -0.88140535

C 4.76653199 1.43412155 -0.24360859

C 4.66193843 -0.95301884 1.16217823

C 5.94712528 0.86411194 0.25901116

C 3.54531201 0.79919844 -0.06092043

C 3.49244010 -0.40926226 0.64267782

C 5.88913269 -0.32564389 0.96263805

H 4.62117210 -1.87342236 1.73538551

H 2.62973046 1.25575580 -0.41732712

H 6.80797522 -0.73652822 1.36228252

C 3.80991593 3.26731588 -1.41870702

H 4.16493619 4.20840952 -1.83237897

H 3.05528968 3.45252374 -0.65169651

H 3.37026093 2.65858707 -2.21391590

O 7.13389617 1.49253235 0.07395819

H 6.96059306 2.30840085 -0.41181987

C 2.17567683 -1.12980435 0.85428649

H 2.19152557 -1.59700602 1.84583817

C 1.88656974 -2.25615164 -0.15036960

H 1.32320854 -1.83242803 -0.98945494

C 3.07612286 -3.01474830 -0.70768637

H 3.77008250 -2.32349781 -1.18645123

H 3.60508592 -3.52090217 0.11097440

O 2.64634009 -3.93802542 -1.68370853

H 1.81643193 -4.31913475 -1.37164787

O 1.07673181 -3.27533309 0.47962327

C -0.25668243 -2.99055440 0.58635624

C -2.93563325 -2.23811096 0.70566066

C -1.09047644 -3.17818085 -0.52231043

C -0.79742215 -2.51899663 1.78006271

C -2.13545071 -2.14562297 1.83730841

C -2.43077737 -2.80096753 -0.46533757

H -2.50795334 -1.71460081 2.76068760

H -3.08344702 -2.90785699 -1.32135729

O -0.47260274 -3.68999432 -1.61923191

O -0.00809727 -2.32539048 2.87784417

C -1.23372092 -3.81494714 -2.80425440

H -0.55584721 -4.21401037 -3.55516413

H -1.61038760 -2.84014204 -3.13153689

H -2.07340039 -4.50115155 -2.66234867

C 0.51951500 -3.52277483 3.44678708

H 1.10356429 -3.21705485 4.31311304

H -0.29603619 -4.17699187 3.76813377

H 1.15668080 -4.04625078 2.73187527

C -4.26840416 -1.50611232 0.69463156
C -3.95040066 -0.01185974 0.56845855
C -3.76935065 0.12311755 -0.94668378
C -4.86447996 -0.81317814 -1.46467415
O -5.05592519 -1.80722557 -0.45120382
H -4.85926984 -1.75166683 1.58095103
H -3.04884466 0.26333984 1.11475181
H -2.78474609 -0.21003316 -1.26368574
H -4.58395517 -1.30843368 -2.39767457
H -5.80782577 -0.27951104 -1.61877141
C -3.99349818 1.62007704 -1.14321726
O -5.03966061 1.94613827 -0.24100889
C -5.08889377 0.98242170 0.82519803
H -4.33489158 1.87927265 -2.15082980
H -6.06743881 0.49389236 0.79932455
H -4.97619992 1.50314543 1.77845448
C -2.69860699 2.35151152 -0.82220873
C -0.18533962 3.44150432 -0.04745222
C -1.53127585 1.96026347 -1.50316488
C -2.65922585 3.30701857 0.19535500
C -1.44593524 3.86277582 0.57013532
C -0.30645585 2.45454521 -1.12209046
H -1.60129772 1.23105044 -2.29894582
H -3.58786016 3.58142486 0.67659603
O 0.86542064 2.07705768 -1.64373383
O -1.28841656 4.78198245 1.52156829
C -2.44432346 5.25141964 2.19255754
H -2.09435095 5.99727922 2.90115487
H -2.93990249 4.43663433 2.72794269
H -3.14425534 5.70774222 1.48746478
C 0.87259164 0.92721197 -2.47587786
H 1.91978693 0.69558422 -2.66272357
H 0.36557718 1.12640356 -3.42403700
H 0.39920862 0.09052358 -1.95396140
O 0.90977089 3.87255818 0.32383405
O 1.05741960 -0.25576308 0.74947205
H 1.10273903 0.38725339 1.46430190

β-O-4 topO-C_{PhO}-side

O -2.17096352 1.34155916 0.10560108
C -2.26913064 0.08355285 -0.38135194
H -2.00972191 0.04302666 -1.44952034
C -3.81261521 -0.23998279 -0.31417729
H -3.92918882 -1.25963446 -0.68262440
H -4.11845080 -0.18830003 0.73350804
O -4.55449142 0.59546263 -1.14840456
H -4.45354587 1.50213669 -0.84438090
C -1.48043165 -0.94007948 0.46018219
H -1.83140664 -1.94523623 0.21183827
O -1.78806205 -0.75356273 1.82755653
H -1.61044246 0.17036432 2.03872467

C 0.00522494 -0.87132074 0.17278585
C 2.75919962 -0.76901547 -0.29883923
C 0.67864066 0.35820376 0.21159262
C 0.72061730 -2.02688959 -0.10396190
C 2.09455584 -1.97931861 -0.34116478
C 2.04219013 0.40790328 -0.01855680
H 0.12758255 1.26919456 0.41633019
H 0.20883981 -2.98225710 -0.13042708
H 2.66335861 -2.87458548 -0.55917185
O 2.81094569 1.53501862 -0.01212625
O 4.09203945 -0.71127710 -0.53030622
H 4.36985891 0.20967151 -0.45626182
C 2.16517627 2.76614417 0.25184067
H 2.93773470 3.52998665 0.20989468
H 1.40062793 2.97362205 -0.50213735
H 1.70844526 2.75922185 1.24556694

β-O-4 topO-C_{Ph} phenyl-side

O -3.43228931 3.67398102 0.54626726
C -3.54241327 2.45443712 -0.04657483
C -3.99187002 0.08712217 -1.42514385
C -4.61443104 2.36034339 -0.95303385
C -2.71053557 1.36821468 0.16667413
C -2.93826967 0.17322013 -0.52738635
C -4.83350162 1.17994412 -1.63807349
H -4.15728456 -0.83187363 -1.97676436
H -1.85234043 1.44466108 0.82270477
H -5.65639662 1.13343609 -2.34048359
C -2.35560365 3.87064038 1.44786465
H -2.43513704 4.89804132 1.79470218
H -1.39965987 3.72498178 0.94002275
H -2.43799850 3.18702289 2.29804414
O -5.41637325 3.43221626 -1.16198682
H -5.08803148 4.15720769 -0.61578206
C -2.02549306 -1.00516840 -0.29226789
H -2.14813169 -1.71701775 -1.11765168
C -2.35792124 -1.75375981 1.01772265
H -1.84442364 -1.26378070 1.85250307
C -3.84390762 -1.86585089 1.31772163
H -4.26831056 -0.87443789 1.47221749
H -4.34997440 -2.32587494 0.45691372
O -4.06395474 -2.60761999 2.49619130
H -3.50677936 -3.39138995 2.44740920
O -1.92295501 -3.14105068 0.96830418
C -0.61542821 -3.30319504 0.60382182
C 2.00302820 -3.10276545 -0.32010377
C 0.41782108 -2.95938219 1.49249829
C -0.30043364 -3.69510730 -0.69911232
C 1.01090098 -3.58786592 -1.16058877
C 1.72569582 -2.85285343 1.02451629
H 1.20765620 -3.81034719 -2.20354378
H 2.52842060 -2.53047272 1.67341083

O 0.02084489 -2.65178748 2.74748228
O -1.27215981 -4.04861196 -1.58631979
C 1.00003483 -2.15812285 3.64258724
H 0.47885400 -1.96326065 4.57654947
H 1.43912632 -1.22899490 3.26538486
H 1.78812211 -2.89753062 3.81001776
C -2.07951579 -5.16268156 -1.20365428
H -2.73134087 -5.36581592 -2.05085478
H -1.44653186 -6.03325279 -1.01151398
H -2.67463444 -4.93040232 -0.32035916
C 3.30775036 -2.61831753 -0.92098306
C 3.08888626 -1.17732829 -1.40451266
C 3.50305903 -0.33522878 -0.19066836
C 4.61665265 -1.19800707 0.41631502
O 4.35267547 -2.54935048 0.03866076
H 3.62595253 -3.28492088 -1.72835387
H 2.05510258 -0.99919837 -1.70362495
H 2.68555344 -0.18713081 0.51478049
H 4.64610901 -1.14095831 1.50732653
H 5.60023335 -0.90774056 0.03077882
C 3.95362414 1.00571516 -0.81322335
O 4.07494081 0.76616678 -2.21493231
C 4.04252834 -0.63117093 -2.46361203
H 4.93198802 1.30485070 -0.41798787
H 5.04287881 -1.07310466 -2.35799343
H 3.69696295 -0.78066310 -3.48647127
C 2.94943578 2.09945013 -0.52631845
C 1.04670789 3.87799867 0.22932380
C 3.22637365 2.99523465 0.50492862
C 1.72585265 2.10380321 -1.19179771
C 0.73432180 3.01455879 -0.79990439
C 2.24708576 3.91957569 0.89774421
H 4.19378342 2.96607215 0.99089805
H 1.55472165 1.40425787 -1.99916017
O 2.39608506 4.83990521 1.88245039
O -0.50026349 3.10763518 -1.33862386
C -0.83487056 2.19831617 -2.37959511
H -1.88489774 2.37914731 -2.60393782
H -0.70979580 1.16724736 -2.04257982
H -0.22398888 2.39364693 -3.26619690
C 3.64480716 4.88741821 2.54195276
H 3.56795831 5.68978982 3.27190148
H 4.45397608 5.10966352 1.83982829
H 3.85614670 3.94555522 3.05785008
O -0.69558450 -0.48293632 -0.28130156
H -0.06784560 -1.21091683 -0.23221886

S.4.Optimized geometries of the radical species produced from the RSSRRSSR stereoisomer of MC2

4-O-5C_4-OC_4-side

C 3.86666386 -0.75107923 1.67601687
C 4.18579296 1.05296733 -0.44619127
C 3.09236477 -0.87272463 0.51862022
C 4.79913870 0.28068786 1.78435167
C 4.91950326 1.13936881 0.72020437
C 3.24629806 0.02017856 -0.53483267
H 5.41088272 0.39067033 2.67247505
H 2.63957901 -0.07093142 -1.42754355
H 3.74612199 -1.46042823 2.48823409
C 2.03246559 -1.93928085 0.45793203
O 1.79112090 -2.32244220 -0.88455357
C 0.53579378 -2.97094732 -0.88517560
C -0.35419636 -2.09168109 -0.00812055
C 0.64031090 -1.53020598 1.04264064
H 2.38409840 -2.81891270 1.01637124
H 0.19783100 -3.05758774 -1.91826493
H 0.62916010 -3.97791147 -0.45407932
H -1.17465018 -2.65004776 0.44032663
H 0.50439331 -1.96246103 2.03413743
C 0.33234460 -0.03649103 1.03543466
O -0.03498434 0.24609399 -0.30465707
C -0.86703134 -0.81952345 -0.73777387
H 1.17761210 0.60023047 1.29526395
H -0.50517190 0.17488673 1.71442804
H -0.70940127 -0.90545945 -1.81716437
C -2.33737894 -0.52592750 -0.47445304
C -5.04610745 0.04497078 -0.07285711
C -3.30447231 -1.50873185 -0.65093909
C -2.73334088 0.76150443 -0.10228842
C -4.07564974 1.04215657 0.10156436
C -4.65457223 -1.22580211 -0.45430193
H -3.01539502 -2.50924483 -0.95526541
H -1.97460173 1.52438792 0.01186325
H -5.41741674 -1.98228281 -0.59119595
O 4.31460796 1.89719094 -1.49407329
O -4.58617609 2.25417186 0.47307745
O -6.35811301 0.32583616 0.12866098
C -3.67411184 3.32200547 0.64159611
H -3.13884282 3.52699449 -0.28968899
H -4.26833133 4.18864054 0.92148815
H -2.95490560 3.09776358 1.43455171
C 5.28142605 2.92060580 -1.33900767
H 5.25994092 3.50313475 -2.25705145
H 6.27806098 2.49368619 -1.19177615
H 5.03569082 3.56033306 -0.48591448
H -6.42774713 1.25542995 0.37665078

4-O-5C_4-OO-side

O -4.11085138 2.93153970 1.08236810
C -3.88093344 1.75254558 0.76037563
C -3.57530372 -0.90686346 -0.08493656
C -4.92002355 1.06113558 -0.00332812

C -2.67442558 1.02471164 1.06383961
C -2.52396193 -0.25922357 0.63296061
C -4.76047455 -0.27132493 -0.39984632
H -3.46648894 -1.93854005 -0.40288444
H -1.91130293 1.52247509 1.64787851
C -1.26404214 -1.05520527 0.93203458
O -0.28933233 -0.29201969 1.60451690
C 0.48610638 0.41524440 0.63421025
C 0.39859582 -0.37757082 -0.68502685
C -0.55680028 -1.54324903 -0.34058139
H -1.53017929 -1.88901811 1.59013511
H 0.09204106 1.42708111 0.50277302
H 1.50266156 0.48152782 1.02120252
C 0.41244344 -2.72587656 -0.09916725
O 1.72947799 -2.20331478 -0.15661929
C 1.69651046 -1.11603219 -1.06178115
H 0.28597420 -3.48897950 -0.87619486
H 0.28363662 -3.18926930 0.87990093
H 1.58802533 -1.49856260 -2.08912778
H 0.03693802 0.24397239 -1.50376328
H -1.25660716 -1.76499727 -1.14544697
C 2.96153807 -0.30444140 -0.97265545
C 5.24999437 1.28219533 -0.78899129
C 3.86350394 -0.50632618 0.07514663
C 3.21533742 0.68048823 -1.91872742
C 4.35559276 1.47538357 -1.82698261
C 5.00025669 0.28276357 0.16346018
H 3.65653286 -1.28369400 0.79811792
H 2.52288302 0.83375852 -2.73998604
H 4.56837794 2.24741834 -2.55589832
O -5.70114793 -0.92676847 -1.13391159
O -6.00339111 1.76456446 -0.30036240
O 5.95904378 0.18729552 1.13124301
O 6.36437884 2.04904217 -0.69569242
C 5.77289272 -0.78739643 2.13999536
H 5.74230114 -1.79172481 1.70859118
H 4.85004097 -0.59943009 2.69596590
H 6.62697205 -0.70109699 2.80742152
C -6.97268365 -1.07522401 -0.50228710
H -6.85853191 -1.59408156 0.45395453
H -7.57433537 -1.68066482 -1.17654217
H -7.45147618 -0.10794070 -0.34737471
H -5.85325252 2.64865561 0.08781126
H 6.85562731 1.76599558 0.08485963

4-O-5O-C_5O-side

C 3.48079149 -0.83813154 1.72776798
C 4.02980858 0.70303476 -0.56251834
C 2.78360834 -1.06729975 0.50808656
C 4.42016580 0.14158608 1.81129720
C 4.76006348 0.97916083 0.67798319
C 3.06242442 -0.30620841 -0.60721375

H 4.96882989 0.34082445 2.72393621
H 2.52737907 -0.47413413 -1.53277524
O 5.62307269 1.87275001 0.76580125
H 3.25269491 -1.45623239 2.59062389
C 1.69325129 -2.09676900 0.49063132
O 1.42371046 -2.51637612 -0.83185597
C 0.13637673 -3.10130239 -0.80352392
C -0.70033834 -2.14902139 0.04805409
C 0.32546636 -1.60807249 1.07923176
H 2.02048934 -2.96648075 1.08015277
H -0.21209349 -3.20408708 -1.83140923
H 0.18331455 -4.09681182 -0.34039412
H -1.54681907 -2.64835402 0.51725982
H 0.17728838 -2.00908207 2.08182928
C 0.08803129 -0.10157461 1.03149695
O -0.26336613 0.16009093 -0.31582697
C -1.14892142 -0.87395559 -0.71915306
H 0.95967387 0.50486784 1.27679007
H -0.73881819 0.16538850 1.70374669
H -0.99566685 -0.99900261 -1.79522945
C -2.60244385 -0.50144448 -0.46435060
C -5.28016565 0.20617350 -0.07517743
C -3.61540908 -1.44035825 -0.62123068
C -2.93565031 0.81085906 -0.11866240
C -4.26299060 1.15930837 0.07944009
C -4.95020299 -1.08937033 -0.43085847
H -3.37472318 -2.45963503 -0.90497663
H -2.14215640 1.53978702 -0.02015711
H -5.74842991 -1.81103386 -0.55290574
O 4.21510628 1.36562394 -1.69972104
O -4.71475550 2.40083947 0.42663364
O -6.57664237 0.55307002 0.12009926
C -3.75323538 3.42740041 0.57734882
H -3.20896809 3.59063680 -0.35695049
H -4.30603873 4.32574011 0.84147250
H -3.04578687 3.18346423 1.37503691
C 5.14738513 2.44225835 -1.83818895
H 5.04139441 2.75818112 -2.87356597
H 6.16267463 2.10443949 -1.63869221
H 4.90313653 3.25647006 -1.15827997
H -6.60247035 1.48942951 0.35068453

4-O-5O-C_5C_5-side

C -3.97611135 1.57965739 1.38125838
C -3.71877035 -0.58349695 -0.20544384
C -5.01102590 1.32641371 0.51620680
C -2.82440878 0.84794943 1.50893613
C -2.68339180 -0.26614933 0.67513939
C -4.87331401 0.18610504 -0.29471181
H -3.65965695 -1.44996579 -0.85487243
H -2.04994450 1.09497919 2.22534140

C -1.44321582 -1.14545472 0.77652976
O -0.42706873 -0.54168315 1.55166104
C 0.28972342 0.35730894 0.70586962
C 0.19386686 -0.20117575 -0.72801544
C -0.77036074 -1.40212429 -0.57828585
H -1.71027328 -2.07892617 1.28085402
H -0.15613530 1.35585647 0.75615304
H 1.31243457 0.40718624 1.07858067
C 0.18757570 -2.61718500 -0.57273121
O 1.51054544 -2.10603848 -0.54099822
C 1.48414626 -0.87347413 -1.23461201
H 0.04911516 -3.21824206 -1.47934582
H 0.06022856 -3.25654964 0.30173900
H 1.36963485 -1.06390247 -2.31373624
H -0.16704034 0.55224519 -1.42765886
H -1.48886533 -1.46910436 -1.39319783
C 2.75669237 -0.10172192 -1.00793801
C 5.05665173 1.41234233 -0.55181167
C 3.65514134 -0.48765951 -0.01000507
C 3.02007172 1.02949131 -1.76997527
C 4.16580793 1.78830996 -1.54160283
C 4.79777982 0.26551324 0.21346195
H 3.43972986 -1.37571277 0.56865231
H 2.33026256 1.32712658 -2.55291647
H 4.38611154 2.67244707 -2.12682530
O -5.82540896 -0.14300681 -1.21420039
O -6.12411359 2.10087368 0.39053201
O 5.75423001 -0.00395907 1.15080872
O 6.17683467 2.14348492 -0.32631853
C 5.56254190 -1.14281939 1.96845380
H 5.52461276 -2.05308513 1.36371503
H 4.64136409 -1.05217388 2.55097070
H 6.41780209 -1.18322323 2.63879629
C -7.07760251 -0.54293734 -0.66196373
H -6.94295765 -1.42195778 -0.02441463
H -7.71414316 -0.79831700 -1.50670940
H -7.53109704 0.26750751 -0.08981495
H -6.04827889 2.85002068 0.98933404
H 6.66615863 1.72306463 0.39092381

β-β’Cleavage

C 1.57703434 3.40225063 -2.22315188
C 2.31870124 2.38089451 0.26081354
C 0.66163159 3.40798794 -1.17121125
C 2.83324373 2.83516518 -2.05344728
C 3.19532193 2.29953173 -0.82726243
C 1.04827563 2.91405060 0.06882008
H 3.53666477 2.76045411 -2.87397467
H 0.35250489 2.91116846 0.89749683
O 4.41231971 1.64529488 -0.72102100
C 4.36062933 0.28829653 -0.45413487

C 4.45495111 -2.37441817 0.26351210
C 5.58378334 -0.26945177 -0.08519803
C 3.20218928 -0.46816563 -0.49269058
C 3.23907907 -1.79932011 -0.09324821
C 5.62779375 -1.62456784 0.25369142
H 4.52084572 -3.41043422 0.57917773
H 2.27021292 -0.01728765 -0.80376355
C 1.93284503 -2.54995099 0.09510842
O 0.86764052 -1.94972555 -0.62838205
C 0.16033123 -1.01899203 0.21309378
C 0.80097352 -1.09295022 1.60646395
C 1.49193015 -2.45961734 1.56233861
H 2.03146500 -3.58329990 -0.25180373
H 0.20651766 -0.01870523 -0.22545372
H -0.88627193 -1.33462140 0.24654789
C 0.34142875 -3.40738917 1.95736434
O -0.68509479 -2.61253144 2.55788127
C -0.22975936 -1.27513057 2.71587123
H 0.66849444 -4.17024742 2.66821696
H -0.08833355 -3.90002030 1.08002698
H 0.26952943 -1.17358984 3.69029818
H 1.48492952 -0.26423473 1.79019227
H 2.33643489 -2.53327063 2.24754727
H 1.29498514 3.79838748 -3.19255116
C -0.75371847 3.93158031 -1.39506432
O -1.50013043 3.63285698 -0.21543501
C -2.90263673 3.52870833 -0.36336312
C -3.35479110 2.18010629 -0.82625672
C -1.34017583 3.37454786 -2.66076508
H -0.70934706 5.02479144 -1.49612429
H -3.32460866 3.73878451 0.62673943
H -3.28273053 4.30686861 -1.04233003
H -4.37367835 2.08625671 -1.18007601
H -1.56376956 4.03870116 -3.48649212
C -1.45866351 1.89810124 -2.86248240
O -1.26627891 1.19679493 -1.65322862
C -2.44229536 0.99498341 -0.85298144
H -0.67680055 1.54272314 -3.54769984
H -2.42382737 1.63948653 -3.32320558
H -2.02339921 0.85238134 0.15284401
C -3.14027452 -0.29334605 -1.27201203
C -4.31603518 -2.74683340 -1.93970897
C -4.46299415 -0.56000693 -0.94035139
C -2.39728127 -1.27300556 -1.94460450
C -2.97535276 -2.48897702 -2.26738357
C -5.05116119 -1.77999736 -1.28070112
H -5.04875018 0.16771484 -0.39111855
H -1.36254421 -1.07040252 -2.18822932
H -6.08460516 -1.99482616 -1.03511647
C -1.37757831 -0.29527061 2.64704435
C -3.44868581 1.56533525 2.47235277
C -2.69111935 -0.73556734 2.47536800
C -1.11114773 1.06845302 2.72714817
C -2.14397165 1.99941924 2.63590491

C -3.72073284 0.19221883 2.39413528
H -2.87941997 -1.79824533 2.40531405
H -0.08852049 1.41231351 2.84970744
H -1.94832124 3.06437282 2.68149111
O 6.79641137 -2.20498389 0.65475740
O 6.70801512 0.49337868 -0.01877521
O 2.60751752 1.87355375 1.49326237
O -2.34831941 -3.52000099 -2.90271684
O -4.87909590 -3.93797657 -2.26587747
O -5.04516520 -0.10581740 2.24254871
O -4.46597259 2.46532651 2.37899273
C -5.40738486 -1.47676156 2.27504671
H -5.12042082 -1.92507955 3.23037191
H -4.93820677 -2.02055071 1.45158448
H -6.48923536 -1.50651678 2.16383302
C -0.97207816 -3.35142751 -3.21050591
H -0.39271623 -3.12832039 -2.31060740
H -0.64558120 -4.29172538 -3.64902014
H -0.83913021 -2.54418555 -3.93691024
C 7.79574419 -2.29048153 -0.35740539
H 7.42546559 -2.87959671 -1.20198858
H 8.64566583 -2.79694952 0.09591534
H 8.09608080 -1.29839804 -0.69771951
C 3.88323399 2.17818080 2.05637429
H 3.73474929 2.24363197 3.13336175
H 4.25102102 3.13517711 1.67950702
H 4.60605813 1.38943788 1.83866351
H 6.47147183 1.39041801 -0.28472530
H -5.29001942 1.96886904 2.30157029
H -4.20533304 -4.46988776 -2.70667625

β-β′ bottomC_{α}-C_{β}

C 1.81866875 3.74025549 -1.79966606
C 2.49954609 2.33256041 0.52407544
C 0.86393119 3.58934238 -0.76564869
C 3.04888192 3.12161435 -1.70103062
C 3.38543668 2.38836553 -0.56500478
C 1.24574004 2.90208793 0.40880308
H 3.76255140 3.15574683 -2.51554814
H 0.54979255 2.80543842 1.23156789
O 4.57135541 1.67197925 -0.56055885
C 4.46109710 0.29660156 -0.45274175
C 4.44181518 -2.43107088 -0.03610882
C 5.65813635 -0.34995856 -0.14931648
C 3.27164349 -0.40216199 -0.57600084
C 3.25194090 -1.76930800 -0.32567136
C 5.64485262 -1.73509360 0.03588110
H 4.46316894 -3.49760837 0.16247679
H 2.36052784 0.11922841 -0.83509913
C 1.91808459 -2.48685768 -0.22145992
O 0.87168672 -1.76759273 -0.85766778
C 0.21072760 -0.91246222 0.09327772

C 0.83017981 -1.19759937 1.46873507
C 1.48102670 -2.56752082 1.24826179
H 1.97818302 -3.47260766 -0.69413877
H 0.31517108 0.13056514 -0.21385057
H -0.85183585 -1.16870537 0.08450605
C 0.30531586 -3.52573667 1.51723140
O -0.69248051 -2.79502275 2.23311688
C -0.22279062 -1.48668006 2.53579859
H 0.61059089 -4.39205172 2.10913875
H -0.14244140 -3.87633541 0.58160803
H 0.25557758 -1.49422528 3.52549612
H 1.53916530 -0.42498668 1.76650125
H 2.32231264 -2.75356946 1.91581885
H 1.55887012 4.29184227 -2.69618104
C -0.45793336 4.04257225 -0.92309881
O -1.36029507 3.72730420 0.02318445
C -2.73169625 3.69920326 -0.36234249
C -3.07633257 2.54849558 -1.32813189
C -2.62056636 2.75093166 -2.74090595
H -0.80396903 4.57472105 -1.80330206
H -3.28140512 3.56950030 0.57078215
H -3.00597751 4.66152686 -0.81116516
H -4.16285457 2.43381016 -1.28193811
H -3.15334027 3.31586681 -3.49181696
C -1.45600610 1.85996600 -3.01418426
O -1.15759391 1.26056977 -1.76033345
C -2.34826140 1.22782378 -0.97744422
H -0.55465855 2.38926366 -3.34580795
H -1.69643889 1.09117298 -3.76549531
H -2.02084689 1.20698573 0.06651066
C -3.16658791 -0.01850615 -1.28046211
C -4.57266075 -2.40052672 -1.70260618
C -4.49858616 -0.13117184 -0.90358278
C -2.53209467 -1.11307833 -1.88012174
C -3.22415962 -2.29664399 -2.07783501
C -5.20320822 -1.31470545 -1.12314973
H -5.00231246 0.69025075 -0.40690755
H -1.48955582 -1.02555366 -2.15992041
H -6.24414136 -1.41140750 -0.83785135
C -1.36492965 -0.49558479 2.54299184
C -3.42899637 1.37766471 2.46085010
C -2.68236890 -0.92279941 2.36991735
C -1.09320326 0.86103369 2.69223398
C -2.12294639 1.79928269 2.64585164
C -3.71028871 0.00936780 2.34471949
H -2.87550991 -1.97985838 2.24936695
H -0.06882962 1.19295885 2.83027737
H -1.92280921 2.86080864 2.73485082
O 6.78670526 -2.40535546 0.36919287
O 6.81232437 0.35363163 0.00353937
O 2.78058077 1.65278911 1.67334456
O -2.70543840 -3.43371165 -2.62405899
O -5.24747087 -3.56235395 -1.89970790
O -5.03751189 -0.28067788 2.21100977

O -4.44180222 2.28769640 2.37998591
C -5.40494228 -1.65043586 2.24164558
H -5.08747526 -2.10896727 3.18239703
H -4.96751142 -2.18838914 1.39731154
H -6.48985751 -1.67487339 2.16568411
C -1.32502874 -3.41976007 -2.95659580
H -0.71214694 -3.16258303 -2.08793255
H -1.08964775 -4.42290024 -3.30484981
H -1.12979335 -2.69927427 -3.75614027
C 7.78774785 -2.41523271 -0.64470722
H 7.39822704 -2.88554255 -1.55278427
H 8.61251356 -3.00715972 -0.25247789
H 8.13209280 -1.40412165 -0.86675974
C 4.05774536 1.86816082 2.27266387
H 3.90890007 1.79179242 3.34895266
H 4.43530846 2.86301667 2.02604385
H 4.77343898 1.10826043 1.95262282
H 6.61254757 1.28393772 -0.15840110
H -5.27210662 1.79668844 2.34151875
H -4.63753044 -4.19131787 -2.30416217

β-β' bottomC_α-O

C 2.38353686 4.24100047 -1.18566788
C 2.33177766 2.09826847 0.58655310
C 1.15207814 3.85179534 -0.62201585
C 3.53279553 3.49790895 -0.96764844
C 3.50620675 2.39862692 -0.11935128
C 1.16466291 2.78023053 0.28424066
H 4.46052277 3.73236591 -1.47545522
H 0.26797839 2.49003745 0.81287311
O 4.65177939 1.63517003 0.00183577
C 4.55522011 0.27311095 -0.23271812
C 4.50824390 -2.47898225 -0.40612123
C 5.67311450 -0.44930260 0.17980356
C 3.43972773 -0.35653937 -0.76114285
C 3.39025492 -1.74512849 -0.79707583
C 5.65433024 -1.84193013 0.05696498
H 4.51025497 -3.56387388 -0.42592532
H 2.59558204 0.22427160 -1.10388551
C 2.07300776 -2.44166360 -1.11322097
O 1.06941349 -1.53032972 -1.52222696
C 0.44196907 -0.97264266 -0.35570575
C 0.83945226 -1.85188638 0.84231957
C 1.50090608 -3.06490094 0.16950988
H 2.20220505 -3.17073463 -1.91838926
H 0.73505883 0.06964069 -0.22226856
H -0.63429611 -1.00732121 -0.51814094
C 0.30521513 -4.02500207 -0.03226785
O -0.84813542 -3.39681862 0.52838699
C -0.39025247 -2.46457890 1.50502915
H 0.48368482 -4.98018876 0.47264508
H 0.09510237 -4.21729447 -1.08684560

H	-0.08420574	-3.01725985	2.40719822
H	1.50382889	-1.33118737	1.53063060
H	2.27791274	-3.51776799	0.78426619
H	2.41601300	5.08834268	-1.86102936
C	-0.05097253	4.55866654	-1.02347411
O	-1.44248837	3.56854221	1.67006237
C	-2.48669745	3.75571830	0.80995174
C	-2.32318663	3.20357085	-0.60485839
C	-1.31881175	3.93256188	-1.54591278
H	0.11771617	5.57644117	-1.36197369
H	-3.34250546	3.25950270	1.31431153
H	-2.75615605	4.82471442	0.79465348
H	-3.30160054	3.30882332	-1.08506431
H	-1.86251848	4.71911179	-2.07337142
C	-1.00136080	2.76610891	-2.50353114
O	-0.86063436	1.64862526	-1.64998179
C	-1.90953164	1.71278139	-0.68753966
H	-0.07003536	2.88956976	-3.05597163
H	-1.83191466	2.61172623	-3.20701866
H	-1.47968624	1.37646625	0.26104187
C	-3.03701771	0.76253940	-1.05387107
C	-5.00728620	-1.14601736	-1.59677576
C	-4.33589652	0.93268462	-0.58861800
C	-2.72831133	-0.37199007	-1.80982137
C	-3.69511209	-1.33148372	-2.05394236
C	-5.32242418	-0.01236349	-0.86990098
H	-4.59788363	1.79346791	0.01736361
H	-1.72424085	-0.48752849	-2.19477726
H	-6.33717580	0.10775371	-0.50984831
C	-1.47362573	-1.47706097	1.86518258
C	-3.45227259	0.36147655	2.56139117
C	-2.82320285	-1.82239620	1.72616400
C	-1.13090106	-0.22768057	2.36944525
C	-2.12080719	0.69113718	2.72324254
C	-3.80526597	-0.91271130	2.08591758
H	-3.07810251	-2.79485443	1.32595372
H	-0.08837691	0.05915167	2.46643926
H	-1.86445386	1.67104146	3.10949716
O	6.71339764	-2.59318994	0.47891535
O	6.74632109	0.17530135	0.73388151
O	2.24914862	1.09622033	1.51065546
O	-3.48464301	-2.50023230	-2.72575894
O	-5.95440993	-2.09076472	-1.84805367
O	-5.14989005	-1.12440176	2.02738124
O	-4.42535685	1.26973174	2.84924607
C	-5.59217943	-2.39486901	1.58131902
H	-5.24711474	-3.17912995	2.26142930
H	-5.23947482	-2.59580620	0.56776529
H	-6.67909722	-2.35599822	1.58191336
C	-2.12892281	-2.89832343	-2.88734689
H	-1.60987421	-2.86718820	-1.92422100
H	-2.15628587	-3.91625593	-3.27049011
H	-1.61348817	-2.25220516	-3.60215581
C	7.91082204	-2.40676955	-0.27060448

H 7.74008014 -2.66110453 -1.32111854

H 8.64537017 -3.08780843 0.15487047

H 8.26940080 -1.37964169 -0.18911153

C 3.25754615 1.05075285 2.51890586

H 2.77896020 0.65404073 3.41398797

H 3.64053097 2.05259156 2.72586857

H 4.07678112 0.39063742 2.22517287

H 6.56061110 1.12213498 0.74935091

H -5.27674398 0.83653208 2.70757742

H -5.53935383 -2.78634919 -2.37334073

β-β′ bottomC_{β}-C_{γ}

C 2.07909871 4.05478378 -0.91357236

C 2.52161815 2.03235808 0.95452828

C 1.05648177 3.67838128 -0.04294179

C 3.29929166 3.39509248 -0.88062390

C 3.51796974 2.37287692 0.03285690

C 1.29150380 2.68303150 0.89483225

H 4.09273512 3.63686082 -1.57742648

H 0.51715897 2.40333251 1.59746404

O 4.72840798 1.69996505 -0.00577371

C 4.68341716 0.34862455 -0.30140170

C 4.73284861 -2.38634814 -0.65017390

C 5.84473419 -0.35081504 0.01950860

C 3.57245475 -0.29060014 -0.82196122

C 3.57560686 -1.67412085 -0.95373317

C 5.87114665 -1.73351797 -0.18656354

H 4.77491790 -3.46661707 -0.74307519

H 2.69879515 0.28313384 -1.09467168

C 2.27238825 -2.38850158 -1.27520312

O 1.27011884 -1.48725096 -1.71111034

C 0.56532135 -0.96719929 -0.57590793

C 1.02290501 -1.77371118 0.65500405

C 1.69142900 -2.99393170 0.01093940

H 2.41839983 -3.12841305 -2.06727054

H 0.75290303 0.10522063 -0.46836206

H -0.50129930 -1.10343049 -0.75727669

C 0.49715457 -3.95284513 -0.18897232

O -0.61986463 -3.40562813 0.52002277

C -0.14807103 -2.39138124 1.40759587

H 0.71853061 -4.95254075 0.19529569

H 0.21075794 -4.03080129 -1.24156164

H 0.22127311 -2.86867036 2.32772146

H 1.69915515 -1.20287978 1.29063864

H 2.46364028 -3.43481306 0.64087826

H 1.91425394 4.84395290 -1.63928020

C -0.31248335 4.32448242 -0.16368259

O -1.17291403 3.86682465 0.87168094

C -2.47820398 3.66840838 0.33645661

C -2.25228903 3.18558087 -1.10608408

C -1.00420581 3.93311157 -1.43700828

H -0.19518685 5.41751077 -0.08786949

H	-2.99089332	2.94933535	0.97710267
H	-3.03358923	4.61421864	0.33783952
H	-3.07747721	3.46009670	-1.76992481
H	-0.53424455	3.94974285	-2.40913249
C	-1.72330853	1.53099127	-3.48301245
O	-1.21726988	1.27301893	-2.25473635
C	-2.03561687	1.65667941	-1.14547224
H	-1.08271674	1.20234754	-4.28804908
H	-2.80085410	1.56869446	-3.60024831
H	-1.42392779	1.38676450	-0.27754559
C	-3.31744217	0.83852066	-1.11615061
C	-5.58488499	-0.80201529	-1.03041816
C	-4.55644997	1.35783541	-0.76209814
C	-3.22044115	-0.52123005	-1.43576784
C	-4.33419665	-1.33705599	-1.37864626
C	-5.68794760	0.54047356	-0.72255639
H	-4.67063054	2.40403313	-0.50750388
H	-2.26351887	-0.92186937	-1.74000751
H	-6.65961625	0.93734880	-0.45444667
C	-1.25223173	-1.42001365	1.74496848
C	-3.26728479	0.42108390	2.32712296
C	-2.59033266	-1.82576629	1.72749656
C	-0.93556893	-0.11103106	2.08937782
C	-1.94126797	0.81109536	2.37836070
C	-3.59027390	-0.91231429	2.02445138
H	-2.82752926	-2.84861264	1.46430146
H	0.10217698	0.20521947	2.12325565
H	-1.70468253	1.84083833	2.62478279
O	6.97255294	-2.46694822	0.14811418
O	6.91713025	0.28400652	0.56473567
O	2.65617057	1.03208974	1.87098989
O	-4.34622117	-2.67820514	-1.63670404
O	-6.67974276	-1.60485289	-0.98737403
O	-4.92643866	-1.17829297	2.04746259
O	-4.25483281	1.32144631	2.57135808
C	-5.33022621	-2.51184638	1.79736054
H	-4.93731954	-3.18092340	2.56909704
H	-4.98873747	-2.84540654	0.81335994
H	-6.41714898	-2.50824169	1.82007436
C	-3.09158043	-3.29635875	-1.89589362
H	-2.40191681	-3.14207478	-1.05961411
H	-3.29965267	-4.35736966	-2.01511668
H	-2.65436262	-2.90379507	-2.81845888
C	8.13105815	-2.19218288	-0.63518201
H	7.92552116	-2.38935833	-1.69168230
H	8.90534850	-2.87012823	-0.28138485
H	8.45720670	-1.15930577	-0.50508211
C	3.83301936	1.02822516	2.68107029
H	3.53512588	0.63823661	3.65333966
H	4.22063191	2.04257884	2.79812972
H	4.60030016	0.38152142	2.25158119
H	6.70299317	1.22284144	0.62908267
H	-5.09908149	0.87990377	2.41199351
H	-6.41505249	-2.48096323	-1.29369059

β-β' bottomC_γ-O

C	1.97161290	4.11681751	-0.71043724
C	2.34277248	1.91521683	0.95428407
C	0.90523854	3.65081938	0.06231336
C	3.18797283	3.45010490	-0.70226595
C	3.37375500	2.33851443	0.10911615
C	1.11071060	2.56259504	0.90020207
H	4.01070263	3.76778190	-1.33131436
H	0.31847336	2.22273954	1.55485309
O	4.59828097	1.69361931	0.06899909
C	4.60676085	0.35195326	-0.26992109
C	4.75924635	-2.36919447	-0.69100990
C	5.78314611	-0.31688524	0.06207124
C	3.53334686	-0.30919257	-0.83894083
C	3.58624864	-1.68820005	-1.00580912
C	5.86279443	-1.69141534	-0.18169233
H	4.84041820	-3.44459258	-0.81054951
H	2.64828459	0.24330477	-1.11834759
C	2.31485008	-2.43730469	-1.37461637
O	1.29035352	-1.55463269	-1.79706981
C	0.55833711	-1.09301915	-0.65476009
C	1.02726692	-1.92139205	0.55630916
C	1.73214369	-3.10284428	-0.11836770
H	2.49834411	-3.14428774	-2.18845061
H	0.71013938	-0.01886476	-0.51053641
H	-0.50078264	-1.26189262	-0.84987660

C	0.56137881	-4.07986305	-0.36524474
O	-0.57385799	-3.58605991	0.35535881
C	-0.13732731	-2.58587386	1.27570468
H	0.80169370	-5.08863447	-0.01842930
H	0.28470249	-4.12302795	-1.42252133
H	0.22910419	-3.07851815	2.18877286
H	1.68193714	-1.35163202	1.21352599
H	2.50658010	-3.54699030	0.50655817
H	1.84241907	4.98868858	-1.34200250
C	-0.42635676	4.37122645	0.03481410
O	-1.28268812	3.84878897	1.04528558
C	-2.59618650	3.67348106	0.52702848
C	-2.37925229	3.28182309	-0.93507836
C	-1.24552124	4.28545216	-1.29410427
H	-0.23057668	5.42882839	0.24911539
H	-3.08786453	2.90879039	1.13183945
H	-3.16551043	4.60884656	0.60050929
H	-3.25214010	3.49183498	-1.55621129
H	-1.77440899	5.24263756	-1.37179677
C	-0.50157431	4.09310008	-2.56462298
O	-1.13999149	1.43030271	-1.98597888
C	-2.04850409	1.77592096	-1.03437745
H	0.31649193	3.39508801	-2.63970820
H	-0.93788172	4.46662389	-3.48171608
H	-1.48373483	1.51275935	-0.10321078

C -3.29064217 0.87896474 -1.06811378
C -5.47818343 -0.86441337 -1.16013111
C -4.55394033 1.30382558 -0.67181999
C -3.12747450 -0.43481685 -1.51570272
C -4.20181754 -1.30581988 -1.53971563
C -5.64538302 0.43647573 -0.72560015
H -4.72127983 2.31151835 -0.31448028
H -2.15385316 -0.74998444 -1.86094382
H -6.63642305 0.76131775 -0.43214616
C -1.26517085 -1.64485331 1.62481123
C -3.32152759 0.13089170 2.27165969
C -2.59512590 -2.07219363 1.57244102
C -0.97681209 -0.34396463 2.02263219
C -2.00353354 0.54463754 2.34458030
C -3.61574380 -1.19194126 1.90012115
H -2.80926346 -3.08684554 1.26232969
H 0.05431787 -0.00964626 2.08039844
H -1.79023656 1.56559346 2.64534483
O 6.98015965 -2.39614071 0.16142046
O 6.81793693 0.33688375 0.65490818
O 2.45631504 0.85176662 1.80174867
O -4.14915236 -2.61622455 -1.92132106
O -6.53727814 -1.71443473 -1.21153346
O -4.94551226 -1.48659824 1.89748908
O -4.32619499 0.99678424 2.56329349
C -5.31781521 -2.80199268 1.52874168
H -4.93371990 -3.52552479 2.25432992
H -4.94177145 -3.04623099 0.53117219
H -6.40464913 -2.81844243 1.51947264
C -2.86246242 -3.15798794 -2.19357598
H -2.20890696 -3.05892731 -1.32083849
H -3.02232801 -4.21052309 -2.41730541
H -2.41165841 -2.66497953 -3.05939605
C 8.14777581 -2.05981899 -0.58349666
H 7.97566774 -2.23320365 -1.65007508
H 8.93590298 -2.72111088 -0.22875050
H 8.43467545 -1.02070050 -0.41601869
C 3.58082301 0.83167318 2.68313171
H 3.24395610 0.35046120 3.60034425
H 3.91072329 1.84895299 2.90505077
H 4.40331523 0.25633432 2.25397128
H 6.56952515 1.26538081 0.74180179
H -5.16107069 0.55355838 2.36383800
H -6.23123674 -2.54195778 -1.60275067

β-β' bottomC_{α}-C_{Ph} α-side

C 4.12564963 0.63888314 0.39146677
O 4.29540997 -0.31311617 -0.56788339
C 3.39214147 -1.37897310 -0.27955504
C 2.17425410 -0.71344405 0.35961189
C 2.77924618 0.53688667 1.05138354
H 4.65297831 1.56351250 0.19862037

H	3.17875672	-1.89724557	-1.21428807
H	3.87175131	-2.07200574	0.41958234
H	1.67333749	-1.38057726	1.05914942
H	2.84658115	0.43714775	2.13795425
C	1.80513556	1.64957285	0.65094955
O	1.38017786	1.29342732	-0.65180293
C	1.16542006	-0.11081956	-0.65844267
H	2.26440130	2.63689820	0.59724149
H	0.95158225	1.68097122	1.34268026
H	1.39504081	-0.44486624	-1.67467800
C	-0.28137240	-0.45964821	-0.33971314
C	-2.95440957	-1.11170216	0.16173353
C	-0.66314611	-1.77950368	-0.12808670
C	-1.25199305	0.54480546	-0.30974301
C	-2.57623090	0.22097912	-0.05557759
C	-1.99439995	-2.10699629	0.11908994
H	0.07640182	-2.57280282	-0.16287602
H	-0.94850401	1.56618319	-0.49732252
H	-2.30470799	-3.13163367	0.28249295
O	-3.61291275	1.10878911	0.00735348
O	-4.25128591	-1.42242292	0.40951221
C	-3.32038995	2.47226533	-0.22884147
H	-2.90472325	2.61314829	-1.23045397
H	-4.26467053	3.00504403	-0.14554152
H	-2.61635441	2.85360734	0.51631114
H	-4.76241483	-0.60463295	0.38803922

β-β′ bottomC_{α}-C_{Ph} phenyl-side

C	-1.03475902	4.19841914	1.93644246
C	-1.08239691	2.53041541	-0.33989182
C	0.04993547	4.01787959	1.11436511
C	-2.18570188	3.47970357	1.60866241
C	-2.20528336	2.65168268	0.49486178
C	0.08514623	3.22312710	-0.00257160
H	-3.07771653	3.53080655	2.22213319
H	0.96218271	3.11072483	-0.62959266
O	-3.36114905	1.93649609	0.23559731
C	-3.28173359	0.55676117	0.31628294
C	-3.27259419	-2.19896455	0.25565876
C	-4.38186274	-0.10959059	-0.22035534
C	-2.19935816	-0.12943935	0.83697542
C	-2.17608325	-1.51770132	0.77527005
C	-4.37957004	-1.50713897	-0.22691901
H	-3.28877392	-3.28218587	0.19524943
H	-1.37108372	0.41171092	1.27187251
C	-0.90257001	-2.25497930	1.14683376
O	-0.04447161	-1.46307720	1.95057650
C	0.90254594	-0.76404338	1.13185227
C	0.68627969	-1.23424288	-0.31709473
C	-0.08377066	-2.54564587	-0.11745771
H	-1.14052586	-3.16196174	1.71037790
H	0.76100780	0.31541087	1.23828868

H 1.90406125 -1.01769844 1.48773874

C 1.05876183 -3.56939772 0.06002449

O 2.27745168 -2.92458659 -0.31753732

C 1.97256529 -1.71105028 -0.98863578

H 0.90504542 -4.45205709 -0.56691707

H 1.16178276 -3.88602821 1.10102505

H 1.76282620 -1.92009563 -2.04821920

H 0.14188732 -0.49774627 -0.90918191

H -0.72160966 -2.79446796 -0.96546995

H -1.00808726 4.84396869 2.80501123

C 3.11747067 -0.73355988 -0.89512968

C 5.16736524 1.14917160 -0.68733462

C 4.27186658 -1.04275127 -0.17411611

C 2.99975788 0.50949292 -1.50749176

C 4.02179143 1.44967631 -1.40414649

C 5.28947056 -0.10480318 -0.07261437

H 4.34731158 -2.01477724 0.29403697

H 2.10219945 0.75247035 -2.06790118

H 3.94874804 2.42215999 -1.87579715

O -5.41809678 -2.20353389 -0.77332953

O -5.42285440 0.57896082 -0.76120109

O -1.04377942 1.71864314 -1.42880174

O 6.46515589 -0.28025944 0.60148395

O 6.16660604 2.06141523 -0.58273677

C 6.66003636 -1.51662603 1.26072381

H 6.64443992 -2.34468040 0.54664289

H 5.89153479 -1.67606329 2.02237603

H 7.63773324 -1.45870364 1.73325797

C -6.65320172 -2.07786611 -0.07303348

H -6.53794607 -2.42777723 0.95726092

H -7.36548636 -2.71260143 -0.59646065

H -7.00473713 -1.04514495 -0.07933504

C -2.12464798 1.79767974 -2.36163467

H -1.69285105 1.61310454 -3.34404016

H -2.57688242 2.79145924 -2.34139376

H -2.87874726 1.03830563 -2.14826067

H -5.24354730 1.52081299 -0.65265308

H 6.86957732 1.67133802 -0.04961614

β-β’ topC_{α}-C_{β}

C 1.76417953 3.87166984 -1.47403161

C 2.30079171 2.22922527 0.71416867

C 0.79694409 3.69666421 -0.48383577

C 2.97189115 3.19207558 -1.39678703

C 3.23771627 2.36085633 -0.31764735

C 1.07703530 2.88364509 0.60542734

H 3.72144679 3.27739672 -2.17414473

H 0.34492747 2.75777315 1.39182690

O 4.43963062 1.67468160 -0.29644182

C 4.38079126 0.29199048 -0.31379294

C 4.44885151 -2.46089525 -0.15135175

C 5.58642685 -0.33645671 -0.00621236

C 3.22924929 -0.43170895 -0.56298301
C 3.25086970 -1.81687502 -0.44451611
C 5.61787464 -1.73254085 0.05130932
H 4.50281077 -3.53996684 -0.05167512
H 2.31230462 0.07981767 -0.81963525
C 1.93677695 -2.57563673 -0.49618634
O 0.91263837 -1.79201839 -1.09139483
C 0.17477314 -1.08860511 -0.07413271
C 0.76676603 -1.50798218 1.28019201
C 1.42900059 -2.84141851 0.92750218
H 2.04263071 -3.49226775 -1.08537907
H 0.22554137 -0.01114945 -0.25692978
H -0.87172762 -1.39159247 -0.14821524
C 0.23396689 -3.80504925 1.01704896
O -0.72685559 -3.20897033 1.89102217
C -0.30834538 -1.90333466 2.28736198
H 0.51398917 -4.78239233 1.41546686
H -0.22944808 -3.94220024 0.03308798
H 0.13281560 -1.96781669 3.29129704
H 1.46553462 -0.76816173 1.66940452
H 2.23352796 -3.11628864 1.61004605
H 1.56951429 4.52604935 -2.31762790
C -0.51838041 4.42173024 -0.58486362
O -1.33939880 4.12093429 0.53019644
C -2.66413367 4.53172381 0.21198173
C -2.74803753 4.41306347 -1.27496663
C -1.39362743 4.11678363 -1.83178002
H -0.31627211 5.50450153 -0.58969318
H -3.36115194 3.88572362 0.75846565
H -2.82774740 5.56716871 0.55183804
H -3.65715977 4.49610676 -1.85200667
H -1.13823953 4.81330574 -2.64198104
C -1.22411252 2.71665837 -2.45080102
O -1.35051720 1.62180819 -1.55190951
C -2.61613223 1.31042822 -1.16102694
H -0.21794452 2.62747454 -2.86377964
H -1.94979704 2.60067652 -3.26412519
H -3.19008432 2.06977985 -0.64285255
C -3.15091269 0.03078284 -1.38702748
C -4.29579767 -2.50574802 -1.75065410
C -4.45542391 -0.25676207 -0.92341079
C -2.43294881 -0.98610915 -2.07403621
C -3.00032068 -2.23178974 -2.23709864
C -5.01734505 -1.50553099 -1.11167830
H -5.01221953 0.51623718 -0.40583412
H -1.43416568 -0.77187482 -2.43219842
H -6.01912868 -1.73008022 -0.76492016
C -1.47086283 -0.93563891 2.30290985
C -3.55859135 0.90226268 2.16723792
C -2.78773371 -1.39071585 2.23622573
C -1.21217461 0.43074970 2.32799895
C -2.25319364 1.35171820 2.25055238
C -3.82750234 -0.47253679 2.18486621
H -2.97094465 -2.45575955 2.19688015

H -0.18679646 0.78331783 2.37764541

H -2.05679327 2.41752098 2.22065896

O 6.76631410 -2.39040754 0.38501921

O 6.70310710 0.38868994 0.27177325

O 2.49057115 1.43218545 1.80239973

O -2.41705358 -3.29907313 -2.85573085

O -4.83569461 -3.74023646 -1.91427571

O -5.15666293 -0.78566198 2.12326544

O -4.58257289 1.79565345 2.03374164

C -5.49781458 -2.15902932 2.18927762

H -5.15453692 -2.59477205 3.13199751

H -5.06440477 -2.70780959 1.34885211

H -6.58349532 -2.20561695 2.13621378

C -1.12549655 -3.10435197 -3.41214963

H -0.40831344 -2.80603231 -2.64293946

H -0.83969330 -4.05967272 -3.84725839

H -1.16060293 -2.34181875 -4.19610194

C 7.81801589 -2.27388129 -0.56944328

H 7.49386820 -2.67139414 -1.53607119

H 8.64320499 -2.87174168 -0.18738815

H 8.13474064 -1.23577471 -0.68018470

C 3.72473327 1.55580350 2.51093165

H 3.49471760 1.37517313 3.55994050

H 4.13461989 2.56147669 2.39474378

H 4.44838825 0.81471179 2.16613866

H 6.47737343 1.32303165 0.18711340

H -5.40924820 1.29856297 2.06861989

H -4.18668550 -4.28370817 -2.37808981

β-β' topC$_{\alpha}$-O

C 2.04093685 4.05699787 -0.71198741

C 2.44791943 1.90192707 1.00414214

C 0.98914114 3.59621683 0.08194687

C 3.26369579 3.39934530 -0.70443741

C 3.46124166 2.30541648 0.12441114

C 1.21374529 2.53913538 0.95442374

H 4.07261079 3.70036217 -1.35875911

H 0.42069733 2.19941114 1.60656035

O 4.67100197 1.63702019 0.05738140

C 4.63393286 0.29357039 -0.27815148

C 4.70752284 -2.43010600 -0.69602096

C 5.79863640 -0.40511594 0.03226127

C 3.53052087 -0.33868809 -0.82443248

C 3.54652367 -1.71932942 -0.98910589

C 5.83804640 -1.78149678 -0.20944130

H 4.75770653 -3.50738930 -0.81602488

H 2.65505753 0.23693581 -1.09216899

C 2.25420284 -2.44187391 -1.33682986

O 1.24621984 -1.54222770 -1.76127180

C 0.52409622 -1.05954423 -0.62078124

C 0.98547540 -1.88357645 0.59722294

C 1.66828420 -3.08257220 -0.06919410

H	2.41595557	-3.16218589	-2.14376858
H	0.68809565	0.01434125	-0.49059336
H	-0.53789233	-1.22327071	-0.80998769
C	0.48171601	-4.04496904	-0.29803215
O	-0.64105196	-3.52812235	0.42528011
C	-0.18416349	-2.52460472	1.33311599
H	0.71054007	-5.05365879	0.05702306
H	0.19728720	-4.09474673	-1.35299252
H	0.18339013	-3.01374383	2.24752136
H	1.65362828	-1.31754786	1.24465690
H	2.43959917	-3.53163958	0.55611250
H	1.89737324	4.90967715	-1.36728289
C	-0.37773333	4.23165518	-0.01893332
O	-1.21032822	3.74036895	1.01710576
C	-2.54572515	3.69998007	0.54531491
C	-2.42999997	3.21254166	-0.90981311
C	-1.15620006	3.97143990	-1.35948342
H	-0.26029068	5.31699451	0.10346988
H	-3.11978242	3.03118565	1.18743246
H	-2.98613314	4.70715319	0.57601442
H	-3.28919972	3.57524740	-1.48421589
H	-1.48260275	4.94445709	-1.73929852
C	-0.35115558	3.33008768	-2.49617259
O	0.39567126	2.22548153	-2.16863215
C	-2.34968605	1.72612367	-1.04242524
H	0.27082695	4.07215863	-3.01545802
H	-1.06199263	2.95726025	-3.25801752
H	-1.37803461	1.25660823	-1.12644934
C	-3.49564715	0.89198839	-1.09056947
C	-5.70838882	-0.84930355	-1.22052358
C	-4.80719374	1.34972078	-0.83157960
C	-3.33027416	-0.47926435	-1.41826492
C	-4.40896573	-1.32961242	-1.47633730
C	-5.89245471	0.48703511	-0.89813401
H	-4.97602233	2.38371922	-0.55583023
H	-2.33483876	-0.84309488	-1.62930671
H	-6.89959718	0.83253379	-0.69736978
C	-1.29539775	-1.56170784	1.67536448
C	-3.30857233	0.28672612	2.23974364
C	-2.63521667	-1.95791772	1.62429585
C	-0.97870144	-0.25856790	2.04234090
C	-1.98218664	0.66647964	2.32321238
C	-3.63435076	-1.03787023	1.90790813
H	-2.87566714	-2.97449927	1.34023697
H	0.05995588	0.04976803	2.09771503
H	-1.74323516	1.69321925	2.57821636
O	6.94360610	-2.51446043	0.11310294
O	6.86211312	0.22304165	0.60241523
O	2.57883427	0.85276146	1.86455943
O	-4.35359338	-2.66451163	-1.76503577
O	-6.76781675	-1.69614793	-1.27428271
O	-4.97433149	-1.28561324	1.87401332
O	-4.29551384	1.19333125	2.46907061
C	-5.39011892	-2.60731358	1.58593539

H -5.04355068 -3.29536909 2.36330735

H -5.01321026 -2.93371162 0.61266838

H -6.47709582 -2.58429133 1.56305720

C -3.06662822 -3.22238329 -1.99923659

H -2.41848290 -3.09381405 -1.12660082

H -3.22882580 -4.28193776 -2.18477081

H -2.60498399 -2.76313140 -2.87833784

C 8.10363253 -2.21039450 -0.65674783

H 7.90538480 -2.38275028 -1.71898461

H 8.88203438 -2.89026883 -0.31582911

H 8.42011291 -1.17829792 -0.49913432

C 3.73333605 0.82930449 2.70574841

H 3.41908969 0.37897348 3.64629090

H 4.09350705 1.84389720 2.88987974

H 4.52688107 0.22480167 2.26269942

H 6.63939401 1.15784024 0.69106064

H -5.13658645 0.77029961 2.25265475

H -6.44266522 -2.55574892 -1.56956450

β-β' topC_{β}-C_{γ}

C 1.48046723 3.69629148 -1.83017534

C 2.19447708 2.19231938 0.40371397

C 0.58719462 3.56980419 -0.76981333

C 2.70675542 3.04560056 -1.79835587

C 3.06295748 2.29002779 -0.69398315

C 0.95228726 2.81636249 0.34296160

H 3.40634033 3.11120328 -2.62255396

H 0.28407355 2.72092049 1.19194980

O 4.30734067 1.68536842 -0.69762698

C 4.37121017 0.30588220 -0.63254019

C 4.71228378 -2.41110707 -0.31955924

C 5.64741156 -0.18813564 -0.36318627

C 3.28138655 -0.53334134 -0.77301218

C 3.44263747 -1.90142419 -0.57234361

C 5.81679951 -1.56839280 -0.23224157

H 4.87362788 -3.47210024 -0.15947112

H 2.31174122 -0.11843765 -1.01055709

C 2.21485521 -2.78865515 -0.45824208

O 1.06888573 -2.19363605 -1.04450900

C 0.37978588 -1.40230480 -0.06417504

C 1.08261353 -1.63297359 1.28525953

C 1.84286488 -2.93669884 1.02389681

H 2.38333993 -3.74816440 -0.95542626

H 0.38470754 -0.35313332 -0.36968106

H -0.65728353 -1.74936128 -0.02744883

C 0.76617004 -3.99824092 1.33816269

O -0.30474634 -3.33868490 2.02256098

C 0.11338515 -2.03156798 2.39158586

H 1.16198958 -4.79722320 1.97063944

H 0.35452857 -4.43813747 0.42575112

H 0.65715204 -2.07626904 3.34657797

H 1.72394716 -0.79696969 1.56078999

H 2.72326612 -3.05009229 1.65626452
H 1.21982263 4.30326246 -2.69029513
C -0.75977085 4.24157813 -0.85103418
O -1.04022972 4.81474973 0.42172151
C -2.12345414 5.62222109 0.46568521
C -2.37915106 2.39157430 -0.18362340
C -1.92133490 3.29681597 -1.28102203
H -0.69365136 5.05335368 -1.58857272
H -2.30993032 6.04457269 1.44170668
H -2.39113681 6.16291768 -0.43752087
H -2.92464309 2.70384125 0.69460343
H -2.73852084 3.93442374 -1.63513976
C -1.51569984 2.26743346 -2.35187151
O -1.11208561 1.11790679 -1.62160718
C -2.05064795 0.97892202 -0.54684245
H -0.68140687 2.57127915 -2.98369948
H -2.38046897 2.02723708 -2.98233952
H -1.53271172 0.46027092 0.26659799
C -3.23047745 0.12541952 -1.00227087
C -5.29472641 -1.53078110 -1.89959236
C -4.55448881 0.46175468 -0.76697401
C -2.93234507 -1.05417463 -1.69918762
C -3.95286059 -1.87795727 -2.13797924
C -5.58731555 -0.36444558 -1.21967212
H -4.79461748 1.37383712 -0.23239140
H -1.89599640 -1.28818436 -1.90900255
H -6.62713292 -0.11462788 -1.04571027
C -1.05616868 -1.08871940 2.53061189
C -3.15743019 0.73828739 2.67121949
C -2.35863949 -1.51093757 2.26523252
C -0.81481608 0.23945357 2.87427412
C -1.86448126 1.15222604 2.94566708
C -3.40534626 -0.60124820 2.34195563
H -2.52786267 -2.54491680 1.99677432
H 0.20061843 0.57481700 3.06169274
H -1.69361961 2.19439749 3.19044197
O 7.03982825 -2.09663530 0.06424958
O 6.70226624 0.65344028 -0.19469742
O 2.49004804 1.46911221 1.52168731
O -3.78823908 -3.05591109 -2.80850175
O -6.29236920 -2.34877952 -2.32341364
O -4.71707548 -0.88646311 2.11358534
O -4.18393183 1.63076443 2.69734379
C -5.01206326 -2.15545401 1.55400019
H -4.80902334 -2.95080875 2.27680062
H -4.42553541 -2.31452597 0.64484566
H -6.07059487 -2.14278639 1.30451026
C -2.45717322 -3.49441977 -3.02285009
H -1.92877377 -3.61138324 -2.07198217
H -2.53166632 -4.45551138 -3.52585714
H -1.91130862 -2.78839905 -3.65405179
C 8.01900095 -1.94127827 -0.95950420
H 7.67961397 -2.42509985 -1.88041163
H 8.91846760 -2.43566840 -0.59779873

H 8.22739730 -0.88694431 -1.14692051
C 3.75002873 1.71249384 2.15016812
H 3.59049710 1.57963253 3.21945839
H 4.08500486 2.73377195 1.95635645
H 4.50232591 1.00019131 1.80556597
H 6.38372641 1.55527777 -0.32287945
H -4.99286086 1.14785058 2.48443363
H -5.88806439 -3.09742843 -2.77844640

β-β′ topC$_{\gamma}$-O

C 2.05812827 4.03715103 -1.26709678
C 2.19336311 2.15827912 0.78685911
C 0.98118357 3.88783611 -0.39888494
C 3.19314760 3.24979582 -1.12468660
C 3.26343645 2.31217220 -0.10950149
C 1.06257831 2.95359949 0.62986013
H 4.03867099 3.34357576 -1.79515526
H 0.25030091 2.83007420 1.33991942
O 4.42634887 1.57163621 0.00609866
C 4.35749854 0.20800402 -0.20394987
C 4.38487068 -2.53994917 -0.39052434
C 5.51169236 -0.48114230 0.16468156
C 3.23942904 -0.44746220 -0.68621054
C 3.23384637 -1.83718777 -0.73600584
C 5.52807902 -1.87268810 0.03843664
H 4.41765406 -3.62404865 -0.42089748
H 2.37241584 0.11819440 -0.99676051
C 1.92964647 -2.56630927 -1.01429889
O 0.90117064 -1.67470149 -1.40984336
C 0.28095835 -1.12499261 -0.23538481
C 0.74638531 -1.96717810 0.96476365
C 1.40209968 -3.18062395 0.29011370
H 2.05341331 -3.30247376 -1.81387394
H 0.54537267 -0.06947056 -0.12450560
H -0.79735042 -1.20403670 -0.37073873
C 0.21350906 -4.15524846 0.12993295
O -0.91024809 -3.57499117 0.79011432
C -0.43879991 -2.58656251 1.69851385
H 0.44142323 -5.12813102 0.57695964
H -0.06346506 -4.30295902 -0.91660655
H -0.08617578 -3.07866250 2.61798679
H 1.42838340 -1.41699575 1.61287292
H 2.20228540 -3.61521918 0.88818330
H 2.01889187 4.77758604 -2.05903926
C -0.23478097 4.77758442 -0.57815700
O -0.61546491 5.45114538 0.54887097
C -2.55603443 3.44734839 1.02013040
C -2.35868946 3.16491819 -0.42681969
C -1.48754337 4.12812511 -1.26210860
H 0.04445469 5.57061673 -1.29795984
H -1.96780968 4.18936238 1.54510994
H -3.35425173 2.95179748 1.55866973

H	-3.34327384	3.19118241	-0.91220409
H	-2.10045061	4.96217214	-1.60786886
C	-1.07520528	3.20209425	-2.40719426
O	-0.80344065	1.95849484	-1.78674566
C	-1.76181308	1.75629929	-0.74939607
H	-0.17484060	3.51394956	-2.93868419
H	-1.90036970	3.10331486	-3.12490207
H	-1.21211970	1.37587075	0.11824858
C	-2.79458529	0.72092515	-1.15156762
C	-4.57492669	-1.34041285	-1.77093132
C	-4.07353273	0.70545969	-0.61024232
C	-2.40833104	-0.30010053	-2.02890853
C	-3.28349321	-1.33155792	-2.32134166
C	-4.96786381	-0.31638996	-0.93121321
H	-4.38963523	1.47626570	0.08460321
H	-1.41136405	-0.27694211	-2.45055171
H	-5.96771250	-0.33801599	-0.51482091
C	-1.53490737	-1.60471886	2.03278805
C	-3.53443075	0.24090822	2.63260648
C	-2.87607841	-1.95069506	1.84546488
C	-1.20699913	-0.34530684	2.52289454
C	-2.20750752	0.57699757	2.82601278
C	-3.86944115	-1.03391108	2.15266996
H	-3.11493162	-2.92488386	1.44078637
H	-0.16803016	-0.05869618	2.65194156
H	-1.96775100	1.56497961	3.20002666
O	6.62162688	-2.59611859	0.41605253
O	6.58792818	0.17632777	0.67284722
O	2.17516363	1.22034155	1.77608153
O	-3.01014084	-2.39819178	-3.12640338
O	-5.42732156	-2.36233231	-2.05511252
O	-5.21083475	-1.23993107	2.01988820
O	-4.51585635	1.15194655	2.87881096
C	-5.63176199	-2.49878673	1.51979600
H	-5.31177221	-3.30248497	2.18894265
H	-5.24031034	-2.66968092	0.51382485
H	-6.71839805	-2.46207023	1.48463497
C	-1.67716594	-2.52250431	-3.59849902
H	-0.96539425	-2.51897619	-2.76778147
H	-1.63376522	-3.46941779	-4.13214167
H	-1.43368177	-1.70543023	-4.28356410
C	7.78715427	-2.37166761	-0.37305287
H	7.58547402	-2.61936904	-1.41961027
H	8.55247814	-3.03878322	0.01867819
H	8.12297222	-1.33690222	-0.29249665
C	3.30584431	1.14598136	2.64658910
H	2.92165048	0.85105170	3.62229888
H	3.79449451	2.11928367	2.72714115
H	4.01894600	0.39682117	2.29667938
H	6.38132484	1.11887294	0.68299083
H	-5.36376885	0.71879770	2.71897043
H	-4.96918771	-2.96762589	-2.65140719

β-β’ topC_{α}-C_{Ph} α-side

C 0.35787124 3.85217885 -0.84375415

C 1.15759770 1.92796553 1.01084248

C -0.59879476 3.18077430 -0.08186747

C 1.70067233 3.52166635 -0.72275648

C 2.10043827 2.54894735 0.18276523

C -0.19194865 2.23175281 0.84708658

H 2.45751812 3.98530294 -1.34383587

H -0.92456116 1.71057997 1.45064967

O 3.43430560 2.17818696 0.21295995

C 3.70989438 0.87180099 -0.15866928

C 4.38526376 -1.75397249 -0.66674125

C 4.97753778 0.42102741 0.20143825

C 2.79988060 0.04391518 -0.79091497

C 3.11849362 -1.29038595 -1.01012435

C 5.32053190 -0.90464359 -0.08193495

H 4.67221002 -2.78859876 -0.82323716

H 1.83328704 0.42605773 -1.08474622

C 2.02416249 -2.23649989 -1.47578101

O 0.92350324 -1.53529150 -2.02982248

C -0.05304921 -1.26287425 -1.01227393

C 0.43917160 -1.94051567 0.27763062

C 1.43309328 -2.97181759 -0.26477685

H 2.40434028 -2.91811750 -2.24230823

H -0.17165051 -0.18473489 -0.87844601

H -1.01231363 -1.66286996 -1.34692344

C 0.50401951 -4.15742010 -0.60178079

O -0.74965929 -3.92256626 0.04837940

C -0.62545284 -2.81439323 0.92951407

H 0.91871779 -5.10694696 -0.25346476

H 0.31328677 -4.22430085 -1.67624165

H -0.25670297 -3.16333572 1.90528205

H 0.88797335 -1.22702467 0.96883160

H 2.19883150 -3.24714903 0.46052516

H 0.05218637 4.61001290 -1.55742283

C -2.06151243 3.45387680 -0.30704948

O -2.83717179 2.84316527 0.70146560

C -4.16973710 2.83727380 0.22424041

C -4.05834664 2.43643489 -1.26152314

C -2.63678101 2.93110593 -1.65833532

H -2.22853077 4.54198037 -0.27557608

H -4.74545273 2.13168314 0.82219070

H -4.60212794 3.84407923 0.31718219

H -4.85962475 2.89149457 -1.84836191

H -2.65268225 3.73524937 -2.39355362

C -1.97638249 1.67810040 -2.24661232

O -2.67045044 0.56389183 -1.68653334

C -3.95784853 0.95695110 -1.48460877

H -0.91812093 1.57257024 -2.01177186

H -2.10584293 1.65764239 -3.33328083

H -4.59559979 0.21335846 -1.02843087

C -1.94446621 -2.10723658 1.12216746

C -4.32659653 -0.70173466 1.45498074

C -3.10107274 -2.54927766 0.48065769

C -1.98624800 -0.96331884 1.91275617

C -3.17443228 -0.25575727 2.07693520

C -4.28421210 -1.84476263 0.64744808

H -3.04556041 -3.42912939 -0.14570574

H -1.08065369 -0.60905680 2.39611412

H -3.21829755 0.64933765 2.67127987

O 6.53720792 -1.40179016 0.28688804

O 5.85218337 1.23426886 0.85358402

O 1.47774157 0.96745474 1.92049267

O -5.48044159 -2.14862703 0.05536188

O -5.49557796 -0.01293906 1.59244065

C -5.49697171 -3.23562231 -0.85161116

H -5.23858623 -4.16905906 -0.34431765

H -4.79942617 -3.06017428 -1.67553938

H -6.51273093 -3.29647324 -1.23527457

C 7.64616303 -0.83114014 -0.40250189

H 7.54716995 -1.00076750 -1.47897168

H 8.53158812 -1.34419884 -0.03218235

H 7.72712631 0.23772344 -0.19925665

C 2.58341614 1.21303421 2.79056574

H 2.33837076 0.73458746 3.73763643

H 2.71767495 2.28564829 2.94713358

H 3.50017153 0.77573028 2.39126973

H 5.43338752 2.09775576 0.95455688

H -6.18462633 -0.50748440 1.13199928

β-β' topC_α-C_{Ph} phenyl-side

C 1.27961793 -1.71940422 -0.00000033

C 0.56530546 0.90279898 -0.00000011

C 2.28593573 -0.79262286 -0.00000016

C -0.07054644 -1.44047699 0.00000043

C -0.42696626 -0.09382889 0.00000036

C 1.90536454 0.55640384 0.00000041

H 3.33207727 -1.07189165 -0.00000029

H -0.81434601 -2.22700255 0.00000094

H 2.64267065 1.35032334 0.00000090

O -1.70210452 0.39168562 0.00000278

O 0.20632753 2.21224797 -0.00000236

C -2.75863461 -0.54877107 -0.00000171

H -2.71860300 -1.17706309 0.89439902

H -3.68046137 0.02782237 -0.00000335

H -2.71859774 -1.17705998 -0.89440437

H -0.75698202 2.25881013 0.00001043

β''-β'''Cleavage

C 2.41339141 3.92634224 -1.39909383

C 2.83043419 1.96245716 0.51747542

C 1.44702699 3.70290038 -0.42168495

C 3.56999550 3.15056312 -1.44803699

C 3.77719062 2.16612482 -0.49517077
C 1.66826435 2.71646285 0.53711734
H 4.31845451 3.29596054 -2.21732904
H 0.92985856 2.52452940 1.30566707
O 4.93273550 1.40467126 -0.54145640
C 4.72127844 0.03041163 -0.52910010
C 4.18984561 -2.64539945 -0.20532251
C 5.45837612 -0.68680555 0.40726155
C 3.78251978 -0.57398496 -1.34156655
C 3.46609502 -1.91640076 -1.14641904
C 5.19933683 -2.05064809 0.54712738
H 3.98088647 -3.68763845 -0.00198324
H 3.25068624 0.01965191 -2.07362307
C 2.32090738 -2.52235376 -1.96162761
O 1.37595679 -1.55600169 -2.37979158
C 0.78688146 -0.68238737 -1.38914103
C 0.82015886 -1.17675910 0.01106220
C 1.69023029 -3.71799707 -1.30088748
H 2.75321820 -2.86955954 -2.91228200
H 1.27418503 0.29416699 -1.43755524
H -0.24631234 -0.56122558 -1.72087153
C 0.21984353 -3.98044250 -1.23833836
O -0.60733558 -2.93672834 -0.74113354
C -0.13122117 -2.25989868 0.41672728
H 0.06103217 -4.88170081 -0.63164545
H -0.17911305 -4.18276445 -2.24140881
H 0.38531670 -2.98520907 1.06693904
H 1.50766058 -0.74708703 0.72792913
H 2.32744596 -4.58260052 -1.15527515
H 2.25904778 4.70014066 -2.14411899
C 0.14384814 4.46350964 -0.44038642
O -0.37755894 4.53173649 0.87950504
C -1.78583702 4.63512394 0.78280676
C -2.15744935 3.66467700 -0.32978554
C -0.98567795 3.82721482 -1.32522981
H 0.33259223 5.48264339 -0.80279476
H -2.20822229 4.38268434 1.75635944
H -2.07968004 5.66068613 0.52066845
H -3.12926992 3.88127108 -0.77311299
H -1.22847593 4.47961605 -2.16425349
C -0.73203802 2.39388391 -1.76246470
O -0.92102317 1.63845285 -0.57825904
C -2.05320352 2.17116254 0.09907491
H 0.27675379 2.20473084 -2.12930377
H -1.45697350 2.09755949 -2.53338628
H -1.83033508 2.09992505 1.16803454
C -3.30208881 1.36189626 -0.19443537
C -5.58433258 -0.17505354 -0.67821463
C -4.50654824 1.66854026 0.42953397
C -3.24007884 0.27312136 -1.06238355
C -4.37246491 -0.48843690 -1.30581237
C -5.64427831 0.90190959 0.19132267
H -4.56092552 2.49445554 1.13204245
H -2.28960952 0.00653445 -1.50185751

H -6.58652581 1.12253953 0.67825408

C -1.33958260 -1.70956922 1.17259657

C -3.54279432 -0.72263476 2.58622965

C -2.59189293 -2.31722158 1.03122930

C -1.20305509 -0.62582358 2.03234967

C -2.29806838 -0.14324684 2.74695351

C -3.68525145 -1.82058032 1.72397882

H -2.69650584 -3.15508140 0.35503375

H -0.24223232 -0.13668958 2.14222718

H -2.20506953 0.70262670 3.41837458

O 5.84415226 -2.79275246 1.49539127

O 6.34903043 -0.06493113 1.22422166

O 3.04306477 0.99135935 1.45983665

O -4.42251300 -1.58530388 -2.12208874

O -6.69028943 -0.93369073 -0.90864742

O -4.96076580 -2.30100650 1.65064541

O -4.61997255 -0.23497179 3.25090134

C -5.20611232 -3.36945985 0.75745685

H -4.66469931 -4.26728300 1.07191723

H -4.91082853 -3.09940328 -0.26044456

H -6.27755943 -3.55285119 0.79263748

C -3.21215731 -1.97521501 -2.75792607

H -2.43778435 -2.20868905 -2.02118958

H -3.45274989 -2.86186237 -3.34095533

H -2.85992871 -1.18492020 -3.42774313

C 7.24602904 -2.93566147 1.28709084

H 7.43619169 -3.41573136 0.32225258

H 7.60974908 -3.57417877 2.08992721

H 7.75050181 -1.96913217 1.32750222

C 3.71472253 1.46369556 2.62468423

H 3.95769212 0.58664565 3.22130530

H 3.06869944 2.14178364 3.18987038

H 4.63643310 1.98657708 2.35232766

H 6.36842567 0.86638732 0.97123504

H -5.39796778 -0.71037138 2.93288242

H -6.46137074 -1.58790447 -1.57955753

β''-β''' bottomC_{α}-C_{β}

C -1.78552329 3.98876125 1.35862778

C -2.07408493 2.21371759 -0.77762188

C -0.68685608 3.66989356 0.56142126

C -3.00200922 3.35779292 1.14038334

C -3.13293564 2.44289929 0.10535157

C -0.84761030 2.81548441 -0.51968473

H -3.85445063 3.52496375 1.78774482

H -0.00444118 2.57758673 -1.15417155

O -4.31018741 1.72526901 0.00218043

C -4.24546257 0.37740131 0.31179219

C -4.42909389 -2.32772904 0.85543734

C -5.49248117 -0.25564838 0.23765667

C -3.09128515 -0.30668338 0.63647604

C -3.16149015 -1.69800604 0.88693295

C	-5.57273566	-1.62257214	0.53607179
H	-4.52360841	-3.38989392	1.05020330
H	-2.14359018	0.20952606	0.71827996
C	-2.01815705	-2.48390878	1.14454876
O	-0.73146625	-2.09563492	1.02030462
C	-0.20772133	-1.46188164	-0.16872125
C	-0.94149374	-1.79274813	-1.47240421
C	-1.26169266	-3.24265854	-1.66171321
H	-2.12819430	-3.49455886	1.52296787
H	-0.17382875	-0.37888175	-0.01497080
H	0.82050978	-1.82548161	-0.21899698
C	-0.56332356	-3.74815033	-2.87618122
O	0.44012088	-2.78395757	-3.13832915
C	-0.05305856	-1.51183462	-2.73301588
H	-1.24768794	-3.82862547	-3.73870077
H	-0.07874758	-4.71950459	-2.74392489
H	-0.71656742	-1.11757902	-3.51504957
H	-1.84284446	-1.17457435	-1.52614139
H	-2.08382446	-3.75320759	-1.18158653
H	-1.67693360	4.68353980	2.18500602
C	0.69057165	4.14817903	0.94408612
O	1.55287213	4.04660043	-0.17978936
C	2.84514746	3.65614357	0.25515816
C	2.58309734	2.69242074	1.40198462
C	1.35438600	3.30852964	2.09436008
H	0.63434269	5.19627656	1.26374538
H	3.36875903	3.21659517	-0.59456083
H	3.40869786	4.52904606	0.61113704
H	3.43431667	2.60472136	2.07753652
H	1.63241896	3.96170041	2.92232637
C	0.61189518	2.06996922	2.57831233
O	0.78410763	1.11760991	1.54269047
C	2.07826044	1.28995981	0.96397816
H	-0.45869412	2.20309117	2.73248748
H	1.06753020	1.71045063	3.51074100
H	1.92812087	1.26412119	-0.12188977
C	3.01879574	0.16727444	1.35759366
C	4.75975555	-1.91222052	2.02965216
C	4.38751302	0.29207247	1.14713471
C	2.51160202	-1.01682904	1.90238254
C	3.37823183	-2.04969056	2.23263446
C	5.25793409	-0.74115708	1.48758521
H	4.78859547	1.20039051	0.70825423
H	1.44370648	-1.10814911	2.06080208
H	6.32651081	-0.65624168	1.32959781
C	1.12725950	-0.57736341	-2.58990988
C	3.34596066	1.10835513	-2.40283829
C	2.38055297	-1.10202934	-2.25213424
C	1.00137236	0.78326155	-2.83432585
C	2.10824246	1.63027507	-2.73300275
C	3.48192607	-0.26835253	-2.16507490
H	2.47595082	-2.16867585	-2.10257635
H	0.03364326	1.19182544	-3.10245867
H	2.02085305	2.69661541	-2.90867298

O -6.76746532 -2.27893652 0.45227201
O -6.60519243 0.43299527 -0.12708831
O -2.13137957 1.33668808 -1.81769658
O 3.01215343 -3.25255487 2.76282306
O 5.59957383 -2.93340801 2.34332954
O 4.75418484 -0.66385984 -1.87966317
O 4.43561438 1.91813419 -2.29878229
C 4.94047311 -2.02411276 -1.52617270
H 4.73141319 -2.67388983 -2.38079701
H 4.29695265 -2.29068248 -0.68331974
H 5.98190456 -2.12471820 -1.22928128
C 1.62401463 -3.48583463 2.94446325
H 1.08343238 -3.39807367 1.99904524
H 1.53812813 -4.49658614 3.33680809
H 1.20342557 -2.77482686 3.66117735
C -7.72250844 -1.89095068 1.43554850
H -7.32712271 -2.07952867 2.43846615
H -8.60182100 -2.50988315 1.26768085
H -7.98796624 -0.83768977 1.33113867
C -3.31855067 1.28078219 -2.60630544
H -3.00018703 1.00259518 -3.61069980
H -3.81087858 2.25493655 -2.63483874
H -4.00932979 0.52679190 -2.22227422
H -6.34974275 1.35251894 -0.26982339
H 5.19765065 1.35544781 -2.11030629
H 5.06660042 -3.64991898 2.70908183

β''-β''' bottom$C_α$-O

C 2.34380609 3.34817742 -1.14924783
C 2.68463987 2.01314670 1.27461753
C 1.29090870 3.29672846 -0.23325757
C 3.52767786 2.67741206 -0.88648813
C 3.69457736 1.98900623 0.30893655
C 1.47395083 2.63835644 0.97525592
H 4.33446214 2.65745431 -1.61061361
H 0.67991945 2.61489981 1.71117473
O 4.86617110 1.25881405 0.50257932
C 4.74977276 -0.07224813 0.10837111
C 4.53268993 -2.65419664 -0.84533779
C 5.60632756 -0.49035240 -0.91040157
C 3.83529064 -0.92252178 0.68815200
C 3.67285060 -2.23911251 0.20327955
C 5.48644061 -1.81114540 -1.38088090
H 4.46172522 -3.65806965 -1.24827972
H 3.22223552 -0.54783977 1.49141454
C 2.65604636 -3.08862353 0.70331783
O -0.23812970 -2.22227018 -0.29431659
C 0.37007850 -1.16493614 0.32803910
C 0.88387314 -1.45550548 1.73426829
C 1.73990849 -2.74489022 1.83679702
H 2.56057989 -4.07255155 0.25658880
H 1.23306343 -0.93242445 -0.33183134

H -0.25276134 -0.26264438 0.29198842
C 0.66422744 -3.82775650 2.08496637
O -0.52891838 -3.16361568 2.49193251
C -0.24151568 -1.79794285 2.72768053
H 0.97244317 -4.53322850 2.86205146
H 0.44340068 -4.37330944 1.16527653
H 0.15802649 -1.67756381 3.74633258
H 1.40127700 -0.56303232 2.09113385
H 2.34299063 -2.63626473 2.75049764
H 2.22826105 3.89084212 -2.08189824
C -0.01229659 3.98813297 -0.55487322
O -0.88431620 3.89896092 0.56153264
C -2.21763978 3.95782269 0.08367509
C -2.18639982 3.11132165 -1.17963428
C -0.80865076 3.44084255 -1.79071392
H 0.20530955 5.04862469 -0.74446990
H -2.87641231 3.56359666 0.85946191
H -2.49931765 4.99519051 -0.14135906
H -3.00180711 3.33241967 -1.86714745
H -0.87233658 4.20645236 -2.56481371
C -0.39253626 2.09322650 -2.36276770
O -0.83404066 1.15177334 -1.40567949
C -2.08360841 1.59836756 -0.86973043
H 0.67967597 1.95461291 -2.49559134
H -0.89717178 1.93342029 -3.32556041
H -2.00048579 1.45409280 0.21277610
C -3.23522201 0.75411193 -1.37955748
C -5.33598684 -0.91064292 -2.17313942
C -4.53323063 1.24197907 -1.46651895
C -2.98586456 -0.59100410 -1.68024058
C -4.02711581 -1.41082410 -2.07899382
C -5.58354394 0.41257163 -1.86500350
H -4.75101363 2.27302697 -1.21192765
H -1.97753498 -0.97555865 -1.58108191
H -6.59954997 0.78137654 -1.93749664
C -1.46654045 -0.93701436 2.58073459
C -3.61042819 0.77936392 2.10502403
C -2.60428241 -1.41646310 1.92931860
C -1.42197985 0.38222404 3.01668806
C -2.49186873 1.24235071 2.77689189
C -3.67764591 -0.56496414 1.70608683
H -2.62508319 -2.45070267 1.61134830
H -0.54127553 0.75173226 3.53258587
H -2.46341646 2.27883191 3.09226431
O 6.32637434 -2.27264645 -2.35335665
O 6.52521862 0.34831021 -1.44717740
O 2.78407441 1.40908044 2.49454163
O -3.91815808 -2.73887312 -2.38063013
O -6.35137802 -1.73648347 -2.53920022
O -4.84403114 -0.90529086 1.09382536
O -4.63545708 1.62582090 1.81889590
C -5.07689368 -2.27801433 0.83634281
H -5.09531924 -2.84368280 1.77283205
H -4.31332295 -2.69111693 0.17286975

H -6.04512610 -2.33175486 0.34215874
C -2.64592446 -3.34043899 -2.19188275
H -2.31300825 -3.23542995 -1.15575628
H -2.76805986 -4.39069188 -2.44629714
H -1.89845481 -2.88324963 -2.84540385
C 6.11643242 -1.69145861 -3.63701936
H 5.09640535 -1.89306935 -3.97863805
H 6.82749482 -2.16870351 -4.30844976
H 6.29672967 -0.61521691 -3.61502996
C 4.02322739 1.51646044 3.19672710
H 3.77519811 1.43845367 4.25389004
H 4.49378276 2.48123169 2.99671712
H 4.70724381 0.71477810 2.91323073
H 6.49437007 1.17141935 -0.94295627
H -5.27376174 1.13709051 1.28121228
H -5.96996060 -2.60136274 -2.73366806

β''-β''' bottomC$_{\beta}$-C$_{\gamma}$

C 2.17881617 3.94896519 -1.21858862
C 2.44280564 2.01062310 0.76510800
C 1.08884956 3.64317567 -0.40399631
C 3.37139611 3.24830973 -1.08685913
C 3.50046701 2.26322955 -0.11776956
C 1.24177513 2.69011283 0.59457905
H 4.21179356 3.43210307 -1.74521285
H 0.42169855 2.46358905 1.26422662
O 4.68020940 1.54011072 -0.06301112
C 4.57229543 0.16806902 -0.22750170
C 4.44142811 -2.58642436 -0.25912875
C 5.65430637 -0.56271813 0.25511910
C 3.45943416 -0.45311164 -0.76602182
C 3.36507987 -1.83854073 -0.73224231
C 5.59245656 -1.95968052 0.20661475
H 4.40517610 -3.66970854 -0.21578092
H 2.65047950 0.13752015 -1.17171604
C 2.02510145 -2.49551372 -1.07618329
O 1.07739009 -1.52838038 -1.49977716
C 0.46560277 -0.95550283 -0.34017673
C 0.59796921 -1.98377848 0.80292198
C 1.41018323 -3.06699832 0.16855426
H 2.14417576 -3.22711645 -1.88013387
H 0.93527590 -0.00457658 -0.08257651
H -0.57524870 -0.76556273 -0.57760895
C -1.93480012 -4.28167634 0.35612244
O -1.46510022 -3.02701948 0.19830207
C -0.77954503 -2.42587869 1.30216066
H -2.01624677 -4.68546884 1.35835808
H -2.56609087 -4.61443066 -0.45516181
H -0.66221137 -3.18275161 2.08705903
H 1.14491055 -1.54861698 1.64936901
H 1.79089349 -3.93546800 0.68839902
H 2.08955206 4.71587119 -1.98133525

C	-0.24382188	4.32645388	-0.61824117
O	-1.06204048	4.11955717	0.52748204
C	-2.40444003	3.88128152	0.13210824
C	-2.28473571	3.14980361	-1.19552387
C	-1.07045777	3.82505983	-1.85354220
H	-0.06410142	5.40199551	-0.73610751
H	-2.88514444	3.30108158	0.92344215
H	-2.94220655	4.82966744	0.00667449
H	-3.18630793	3.21928241	-1.80616449
H	-1.35917542	4.67465955	-2.47372261
C	-0.49344602	2.69176457	-2.69012941
O	-0.69695435	1.51480921	-1.92639942
C	-1.81341477	1.68769156	-1.05200319
H	0.57341633	2.77487549	-2.89815388
H	-1.03680892	2.62789716	-3.64148697
H	-1.42930611	1.53952092	-0.03382146
C	-2.87835841	0.63896371	-1.28633587
C	-4.71641185	-1.45716676	-1.38939777
C	-4.15788536	0.79685010	-0.76709762
C	-2.53423766	-0.56364448	-1.91239898
C	-3.43306581	-1.61600701	-1.93272229
C	-5.08367299	-0.24321387	-0.83853259
H	-4.44158684	1.72230132	-0.27586612
H	-1.54948541	-0.66579110	-2.34869830
H	-6.08443127	-0.13244589	-0.43827912
C	-1.62713645	-1.26466070	1.80293423
C	-3.28185944	0.85493818	2.56910439
C	-3.01858688	-1.43398883	1.84023514
C	-1.08249740	-0.04333845	2.18248415
C	-1.91067212	1.01719599	2.56106016
C	-3.83649054	-0.39137026	2.23233057
H	-3.45118001	-2.37311820	1.51775176
H	-0.01107418	0.12362889	2.16311062
H	-1.49898425	1.98400620	2.83048467
O	6.61184895	-2.71782643	0.70711133
O	6.72706603	0.05863951	0.81664943
O	2.48809627	1.05952431	1.74194991
O	-3.18454603	-2.85598894	-2.44236538
O	-5.59142630	-2.50236505	-1.39477441
O	-5.19731523	-0.43025481	2.29970080
O	-4.09770511	1.89630540	2.88021085
C	-5.82428065	-1.68968705	2.11186565
H	-5.45800564	-2.40873273	2.85052253
H	-5.65165276	-2.07197833	1.10286463
H	-6.88869025	-1.52127894	2.26012707
C	-1.88213538	-3.09295948	-2.96406392
H	-1.11971700	-2.90671143	-2.20326834
H	-1.86698094	-4.13876007	-3.26412032
H	-1.69925679	-2.45882295	-3.83604099
C	7.83762757	-2.61322966	-0.01149638
H	7.69232989	-2.92511268	-1.05026431
H	8.53630108	-3.28905939	0.47794523
H	8.22627709	-1.59424446	0.02088979
C	3.59679145	1.09592571	2.64172345

H 3.22927037 0.72112758 3.59592581
H 3.95420293 2.12079215 2.76609328
H 4.40838562 0.45570394 2.29231408
H 6.57640249 1.01047809 0.76513146
H -5.00663267 1.57132660 2.86545227
H -5.15879997 -3.24050203 -1.84168895

β''-β''' bottomC_{γ}-O

C 2.12396095 3.95261533 -1.43707793
C 2.25728052 2.12282560 0.66134566
C 0.98190503 3.67088818 -0.69166996
C 3.32098247 3.29773244 -1.16259391
C 3.38481089 2.37501388 -0.13106053
C 1.06404135 2.76689992 0.36361285
H 4.21295004 3.47538285 -1.75094073
H 0.19404901 2.54629338 0.96871508
O 4.57241090 1.70425375 0.09977787
C 4.55797737 0.33318467 -0.09585730
C 4.67130138 -2.41310829 -0.30226936
C 5.58371143 -0.34975344 0.55262491
C 3.61065891 -0.32925097 -0.85838580
C 3.65022305 -1.71636818 -0.94484384
C 5.64146816 -1.74148697 0.43355281
H 4.73694174 -3.49483312 -0.35316866
H 2.84280831 0.22839575 -1.37755324
C 2.56709894 -2.45640495 -1.71180104
O 1.65689926 -1.55993397 -2.32570884
C 0.62441165 -1.22858251 -1.39176382
C 0.77633285 -2.19184367 -0.20489997
C 1.66939559 -3.32088848 -0.79455514
H 3.01613753 -3.05796543 -2.50580884
H 0.70623283 -0.18642592 -1.07736729
H -0.33729061 -1.35750018 -1.89700056
C 0.95040581 -4.35315542 -1.59006349
O -1.42953286 -3.16625321 -0.51565613
C -0.56332734 -2.62548994 0.40217967
H 0.61026483 -5.27215899 -1.13340664
H 0.58109223 -4.10524661 -2.57683379
H -0.35899144 -3.51026107 1.04811489
H 1.36765783 -1.71074444 0.57808610
H 2.24772985 -3.78741808 0.00689165
H 2.07945595 4.67164724 -2.24798017
C -0.34748453 4.27908215 -1.06542535
O -1.12386076 4.47983637 0.10888634
C -2.49187929 4.34885352 -0.23460993
C -2.51442675 3.17978858 -1.20910708
C -1.22633460 3.40258419 -2.02724827
H -0.17018857 5.24905431 -1.54691392
H -3.05055783 4.17526286 0.68615919
H -2.86221458 5.26580918 -0.71190196
H -3.41425457 3.14485365 -1.82372951
H -1.40942337 3.93578254 -2.96063717

C -0.74622701 1.98138107 -2.27280310
O -1.07390331 1.27612820 -1.08778525
C -2.25030712 1.82194228 -0.50288855
H 0.33009186 1.88834818 -2.43308337
H -1.27195426 1.55092708 -3.13624091
H -2.02196763 2.00813398 0.55182432
C -3.39412042 0.83617454 -0.56883540
C -5.49256536 -0.99771335 -0.63659564
C -4.51637806 1.02050635 0.22901286
C -3.31790856 -0.27714909 -1.40868750
C -4.36163456 -1.18899717 -1.44385790
C -5.56745913 0.10631574 0.19387716
H -4.56423392 1.86464250 0.91018323
H -2.42166174 -0.43407166 -1.99365595
H -6.44310407 0.22571208 0.82071969
C -1.26006400 -1.58468082 1.28026646
C -2.62828179 0.22362262 2.90908367
C -2.57597962 -1.85885077 1.68330782
C -0.64419382 -0.41661221 1.70257429
C -1.32916414 0.48342723 2.52453603
C -3.25143070 -0.96792392 2.49447737
H -3.05786411 -2.76158106 1.32902066
H 0.36701158 -0.16532764 1.41036091
H -0.85715032 1.39876888 2.86391147
O 6.60163880 -2.45693427 1.08892390
O 6.49383094 0.31286542 1.31696333
O 2.25347550 1.20932851 1.67263498
O -4.41263178 -2.31283496 -2.21442830
O -6.50116746 -1.91235389 -0.65534401
O -4.52310502 -1.11490225 2.96434123
O -3.31425619 1.11106521 3.67406206
C -5.20322021 -2.31708317 2.64767280
H -4.65692772 -3.17961365 3.04090149
H -5.33736836 -2.41891794 1.56847022
H -6.17691230 -2.24962629 3.12775112
C -3.33815237 -2.54281231 -3.11052744
H -2.40392446 -2.69366483 -2.56475134
H -3.59016435 -3.44901931 -3.65654601
H -3.23776526 -1.70765336 -3.81016293
C 7.92907331 -2.24005814 0.61802027
H 8.00140581 -2.51104294 -0.43971157
H 8.57122927 -2.89246895 1.20652409
H 8.23073630 -1.20102516 0.75835962
C 3.25887554 1.32946438 2.68000400
H 2.80058417 0.99780937 3.61071170
H 3.57763923 2.36962719 2.78032124
H 4.11677068 0.69399043 2.45533923
H 6.29729589 1.25599598 1.25743690
H -4.18624057 0.73446801 3.84633426
H -6.27656452 -2.58099151 -1.31440244

β''-β''' bottomC_{α}-C_{Ph} α-side

C 3.80871998 0.43032623 1.03681546
O 3.05083199 -0.16693324 1.99340602
C 1.83196397 -0.58993488 1.37931430
C 2.18764287 -0.91501735 -0.07868174
C 3.44388546 -0.04167689 -0.33896507
H 4.80416074 0.70058838 1.36142982
H 1.44920437 -1.43900946 1.94238747
H 1.11274248 0.23311192 1.42695885
C 2.91435597 1.04979684 -1.30167820
O 1.50035727 1.00020574 -1.20417325
C 1.16539324 -0.37099176 -1.09398163
H 3.21809999 0.83124833 -2.33361491
H 3.23789938 2.05392402 -1.02990263
H 1.34050492 -0.86261163 -2.06352176
H 2.37747634 -1.97930557 -0.21617658
H 4.25636288 -0.60151863 -0.81181742
C -0.28044579 -0.54401845 -0.70484086
C -2.93291687 -0.91540221 0.08059538
C -1.06394001 0.56074783 -0.36339455
C -0.83090595 -1.81902094 -0.65635518
C -2.15386136 -2.00628587 -0.26287870
C -2.38175507 0.37328383 0.02621712
H -0.62345128 1.54725537 -0.41348703
H -0.22910736 -2.67958459 -0.92997193
H -2.59823784 -2.99291069 -0.22052001
O -3.25192420 1.36398036 0.38205637
O -4.22267600 -1.08999196 0.46193331
C -2.77046340 2.69429817 0.35970185
H -2.45307984 2.97567535 -0.64818662
H -1.93445750 2.81643707 1.05411496
H -3.60069908 3.32343226 0.67162136
H -4.59616891 -0.22144674 0.65369767

β’’-β’’’ bottomC_{α}-C_{Ph} phenyl-side

C -0.18211460 -3.37581197 -1.59152628
C -1.12110707 -2.40079392 0.85212541
C 0.66124138 -3.35225907 -0.47950176
C -1.45251460 -2.82674134 -1.50606262
C -1.89310699 -2.28639555 -0.30696530
C 0.15679637 -2.94341785 0.74699516
H -2.09979412 -2.75102077 -2.37175601
H 0.75996246 -2.99623599 1.64431464
O -3.07179626 -1.55952666 -0.29131197
C -2.91786593 -0.18583421 -0.22939773
C -2.90522031 2.59171353 -0.03006497
C -4.10494686 0.49427865 0.05943123
C -1.71177520 0.48739564 -0.42101725
C -1.78299537 1.85457665 -0.30406606
C -4.10103332 1.88829272 0.15355827
H -2.91263344 3.67103340 0.06188007
H -0.78795426 -0.03945345 -0.64214661
H 0.17360171 -3.76705192 -2.53863865

C 2.13806893 -3.63756612 -0.64644401
O 2.78104726 -3.88580421 0.59151827
C 3.38618761 -2.69787442 1.10573245
C 3.34463999 -1.64351529 -0.00530114
C 2.92255698 -2.43677023 -1.25453099
H 2.26914882 -4.52939306 -1.26479990
H 2.85495383 -2.36721826 2.00455320
H 4.41120035 -2.94829354 1.38657544
H 4.32235957 -1.18381308 -0.14942236
H 3.78604942 -2.81792490 -1.80156803
C 2.15648826 -1.38637479 -2.04545952
O 1.45530987 -0.63411534 -1.06840039
C 2.26005675 -0.53742915 0.10896605
H 1.42496890 -1.77691327 -2.75141236
H 2.86524281 -0.74514242 -2.58528563
H 1.58763566 -0.74677873 0.94872346
C 2.80919334 0.86668891 0.27900553
C 3.70471227 3.48166928 0.69598603
C 3.90844259 1.11856551 1.09020435
C 2.14014266 1.93891365 -0.32219246
C 2.58804174 3.23402787 -0.11817353
C 4.35614246 2.42253475 1.29979541
H 4.42737738 0.30092059 1.57904266
H 1.27690441 1.73618243 -0.94299609
H 5.21254935 2.63242972 1.92851661
O -5.23555566 2.57087189 0.47579458
O -5.25914811 -0.19481792 0.27344422
O -1.48793426 -1.91383818 2.06247653
O 2.03255972 4.36051702 -0.65282850
O 4.13854470 4.75227328 0.88761633
C 0.85274693 4.20453205 -1.42022981
H 0.06036527 3.74278355 -0.82392864
H 0.55381023 5.20487611 -1.72461240
H 1.04313946 3.59218958 -2.30630972
C -6.26300743 2.52299538 -0.51169308
H -5.89700013 2.94011028 -1.45474075
H -7.07698131 3.13744641 -0.13225005
H -6.61147012 1.50088954 -0.66527209
C -2.84816991 -2.02729061 2.47311742
H -2.82735037 -2.10676718 3.55871571
H -3.30697254 -2.92318904 2.04831431
H -3.42049741 -1.14279735 2.18776381
H -5.07162132 -1.13253566 0.14371838
H 3.54918311 5.34252747 0.40280366

β''-β''' topC$_{\alpha}$-C$_{\beta}$

C 3.45536738 3.67180505 -1.24051686
C 3.70775907 1.88724202 0.89164582
C 2.62070418 3.79069086 -0.12877306
C 4.33742748 2.60422445 -1.33025564
C 4.42241881 1.68288897 -0.29491541
C 2.78349342 2.92797001 0.94562238

H	4.93171274	2.43210963	-2.21958567
H	2.15240563	3.01747188	1.82029180
O	5.18777082	0.54603075	-0.48705498
C	4.52071041	-0.66818620	-0.51507743
C	3.44780588	-3.20959316	-0.45513507
C	5.36975559	-1.76564525	-0.65027127
C	3.15039870	-0.82567894	-0.36727805
C	2.61344163	-2.10620019	-0.29679123
C	4.81591998	-3.04883409	-0.64594778
H	3.06226452	-4.22325767	-0.41170509
H	2.49948076	0.03245615	-0.28086058
C	1.14851370	-2.29460917	0.06887362
O	0.44212163	-1.06230963	0.09067112
C	0.53042468	-0.47706040	1.39848436
C	0.95466367	-1.58893634	2.28893798
C	1.02519043	-2.86078220	1.50798937
H	0.64687728	-2.93648857	-0.66236442
H	1.23836146	0.36012354	1.40688855
H	-0.46109153	-0.06675257	1.63260130
C	-0.17295295	-3.80886142	1.69064993
O	-1.40823333	-3.28921932	1.22134704
C	-1.99970713	-2.34868015	1.99958372
H	-0.27023288	-4.08488735	2.74711152
H	-0.00618688	-4.71714827	1.10796439
H	-1.45077228	-1.98938213	2.86084223
H	1.25079128	-1.46863249	3.32037044
H	1.91318487	-3.44472881	1.77133506
H	3.35607076	4.36092192	-2.07252343
C	1.41461502	4.68712736	-0.20617260
O	0.87282871	4.90720116	1.08203029
C	-0.50097833	5.20123500	0.91041156
C	-0.99056441	4.22813932	-0.16078085
C	0.26291725	4.04729808	-1.06089236
H	1.68971826	5.65171134	-0.65523637
H	-0.99256714	5.08445287	1.87684073
H	-0.62547781	6.23892764	0.57107419
H	-1.84894798	4.61901924	-0.70543441
H	0.17582893	4.54877153	-2.02472559
C	0.34362909	2.52924203	-1.20555516
O	-0.08763965	2.05331493	0.05793250
C	-1.26321038	2.78877525	0.36570751
H	1.33755775	2.12916416	-1.39797593
H	-0.33626210	2.19030221	-1.99715077
H	-1.35709215	2.78065747	1.45622262
C	-2.48116486	2.12571842	-0.26717601
C	-4.66153842	0.88793829	-1.49776668
C	-3.71564334	2.76071790	-0.32210121
C	-2.34532467	0.84238244	-0.80655230
C	-3.41937794	0.23861444	-1.44063833
C	-4.80767670	2.13843645	-0.92618825
H	-3.84438938	3.74821147	0.10888311
H	-1.38964534	0.33624432	-0.73409028
H	-5.78171733	2.61073917	-0.96019412
C	-3.27099880	-1.85651016	1.65854198

C -5.74226510 -0.73769670 0.91712301
C -4.02325464 -2.41694066 0.59666674
C -3.80991078 -0.75017378 2.35631600
C -5.02256752 -0.20573337 1.98365760
C -5.23303108 -1.85999643 0.23611679
H -3.61798907 -3.26896549 0.06854062
H -3.24690272 -0.31071454 3.17235396
H -5.43162351 0.66053161 2.48922628
O 5.61131861 -4.15336712 -0.74721974
O 6.71496888 -1.60094447 -0.75468122
O 3.75823619 1.04745013 1.95766807
O -3.37790543 -0.98230297 -2.04983809
O -5.72504986 0.28532018 -2.09870357
O -6.00743715 -2.29593970 -0.80582023
O -6.91730885 -0.16614340 0.55405334
C -5.58996509 -3.46965324 -1.47565666
H -5.51520938 -4.30928027 -0.77880832
H -4.62569248 -3.31161928 -1.96776674
H -6.35370306 -3.68103600 -2.22128930
C -2.14145130 -1.67887297 -1.98759072
H -1.82735173 -1.83699554 -0.95205090
H -2.30825943 -2.63572495 -2.48038140
H -1.36137330 -1.12386504 -2.51771370
C 6.27399770 -4.29751144 -2.00062286
H 5.53910978 -4.35975514 -2.80905286
H 6.83329984 -5.22930901 -1.94351625
H 6.95789727 -3.46702612 -2.18118048
C 5.02577541 0.53426663 2.36195550
H 4.97177135 0.41315260 3.44272940
H 5.82468663 1.23544906 2.11124790
H 5.22105269 -0.43492268 1.89847805
H 6.89924263 -0.65413127 -0.72883003
H -7.08950889 -0.38123352 -0.37218010
H -5.46875863 -0.61862578 -2.32523022

β''-β''' topC_α-O

C 1.74596086 3.53007547 -2.24515798
C 2.33141568 2.11979673 0.08337283
C 0.76365751 3.36148892 -1.27333223
C 3.00462215 2.95918014 -2.07901071
C 3.29466678 2.24313177 -0.92795385
C 1.07042504 2.66398201 -0.10902187
H 3.77208942 3.04708862 -2.83822309
H 0.31894846 2.53418977 0.65887583
O 4.54858644 1.67547379 -0.80049719
C 4.59630145 0.32389776 -0.50086194
C 4.81545225 -2.30803483 0.27996463
C 5.72307857 -0.07446671 0.21225585
C 3.60430685 -0.57244288 -0.85759553
C 3.69561166 -1.89247728 -0.43725505
C 5.83368016 -1.41528518 0.59449255
H 4.92116678 -3.33420003 0.61643977

H 2.75159990 -0.23341754 -1.43242727
C 2.53627016 -2.83958024 -0.67806922
O 1.74458120 -2.42283185 -1.77639766
C 0.57146968 -1.74382769 -1.33182136
C 0.63492497 -1.67624746 0.20830672
C 1.58262466 -2.85570183 0.53206547
H 2.91600758 -3.84170642 -0.89909472
H 0.53381479 -0.74422945 -1.76852414
H -0.30080310 -2.31363222 -1.66447502
C 0.85829781 -4.22518014 0.69440684
O 0.11731408 -4.46806606 -0.42761376
C -0.70981917 -1.72447164 0.84529503
H 0.20678296 -4.13165921 1.57521946
H 1.62356674 -4.99551128 0.85717200
H -1.33899481 -2.56692076 0.57151709
H 1.16065373 -0.75957281 0.48787201
H 2.11883404 -2.69158956 1.46900851
H 1.52624473 4.08816836 -3.14943362
C -0.64212677 3.85736876 -1.49750776
O -1.26741292 4.06459151 -0.24057143
C -2.66069002 3.88937099 -0.40964056
C -2.78192924 2.69765211 -1.35124735
C -1.56998423 2.87425494 -2.29403836
H -0.60412088 4.81059089 -2.04159488
H -3.09727443 3.72331816 0.57686026
H -3.10964129 4.78696649 -0.85659800
H -3.73170296 2.68112486 -1.88547166
H -1.84380476 3.30813007 -3.25599725
C -1.06056930 1.44731070 -2.44134547
O -1.25699921 0.86733096 -1.16277820
C -2.50380805 1.33368770 -0.65767159
H -0.00062316 1.37062313 -2.68816655
H -1.64755276 0.91413337 -3.20209687
H -2.35872365 1.47907422 0.41732572
C -3.61207942 0.31776105 -0.86620499
C -5.65277303 -1.57391666 -1.12682248
C -4.92358453 0.64077983 -0.53657293
C -3.31751530 -0.96594488 -1.33111240
C -4.33061698 -1.90598128 -1.46049223
C -5.94324419 -0.30011716 -0.66930405
H -5.16082822 1.63071333 -0.15950377
H -2.28918597 -1.21108678 -1.56239647
H -6.96979884 -0.06278959 -0.41659993
C -1.27496570 -0.74033386 1.68589262
C -2.54558579 1.25665645 3.21972221
C -2.64591341 -0.86050602 2.05441055
C -0.56606682 0.39316533 2.15073312
C -1.19847380 1.37165440 2.90264415
C -3.26493198 0.11918871 2.79515281
H -3.20629400 -1.71503353 1.69459846
H 0.48364054 0.51329261 1.91597960
H -0.66141504 2.24666241 3.25050437
O 6.89884617 -1.84342136 1.33293109
O 6.68681300 0.82016674 0.56269945

O 2.56993885 1.40961894 1.22862228
O -4.17740323 -3.18760089 -1.90207713
O -6.63923758 -2.49920361 -1.24813285
O -4.57491436 0.12336816 3.17922906
O -3.16941962 2.22280318 3.93663613
C -5.34073615 -1.03207411 2.88265904
H -4.90282340 -1.91309567 3.36100500
H -5.40592338 -1.18807322 1.80290570
H -6.33395840 -0.84967488 3.28655529
C -2.86585979 -3.62357574 -2.22234573
H -2.21469276 -3.59678224 -1.34471227
H -2.96314095 -4.65008372 -2.56720120
H -2.43664117 -3.00885991 -3.01893363
C 8.14660898 -1.81688712 0.64476743
H 8.10124526 -2.45878814 -0.24025189
H 8.88673935 -2.20663351 1.34091864
H 8.41305787 -0.79950890 0.35446089
C 3.53023374 1.97988414 2.11505478
H 3.55446438 1.33680966 2.99239383
H 3.22401327 2.98976243 2.40176897
H 4.52387878 2.00825939 1.66326571
H 6.45143078 1.67285995 0.17691528
H -4.08293034 1.94292367 4.07487092
H -6.24285921 -3.30908958 -1.59199515

β''-β''' topC_β-C_γ

C 1.90732480 4.11296678 -1.18212726
C 2.19962962 2.19862088 0.82773254
C 0.82372795 3.78550609 -0.37043739
C 3.12159720 3.45713300 -1.02168609
C 3.26133529 2.49328919 -0.03579134
C 0.98165630 2.83959005 0.63757931
H 3.97009428 3.66480160 -1.66216985
H 0.14977237 2.57417086 1.27592490
O 4.47229524 1.83463473 0.07621659
C 4.49875105 0.48319443 -0.20811304
C 4.79750912 -2.21357756 -0.65262696
C 5.71491209 -0.12225262 0.10742083
C 3.43374787 -0.23376098 -0.73104499
C 3.57897674 -1.60190087 -0.93959833
C 5.86460866 -1.48835744 -0.13471951
H 4.94980587 -3.27482267 -0.81944075
H 2.50082483 0.26662409 -0.95493828
C 2.43403066 -2.47970857 -1.40910678
O 1.43805289 -1.78838919 -2.14964256
C 0.52824420 -0.97553104 -1.55273407
C 1.43164973 -2.60314927 0.97447395
C 1.89822118 -3.34192218 -0.23639546
H 2.83395001 -3.18051184 -2.14741249
H -0.12994386 -0.49570683 -2.26025922
H 0.28603733 -1.06016923 -0.50475735
C 0.66940352 -4.20446123 -0.59183635

O -0.43592979 -3.64695500 0.10047307
C 0.03611735 -3.02781221 1.30244102
H 0.82514492 -5.24185948 -0.27284548
H 0.43440565 -4.18733865 -1.65804382
H 0.05791837 -3.79676408 2.09755765
H 2.08711174 -2.07081598 1.64801484
H 2.74493160 -3.98974986 0.02414977
H 1.79847848 4.86366870 -1.95740294
C -0.53156872 4.37840780 -0.65326178
O -1.27813622 4.47644917 0.54965249
C -2.64903115 4.43901806 0.19762589
C -2.72576733 3.34577183 -0.86096638
C -1.40947539 3.53863227 -1.64758596
H -0.40078117 5.38410387 -1.07482357
H -3.22175508 4.22405318 1.10081320
H -2.96753711 5.40769131 -0.21166055
H -3.61418390 3.41710819 -1.48843448
H -1.54704678 4.07743799 -2.58537672
C -0.94933620 2.10522011 -1.86904196
O -1.29329860 1.44222126 -0.66565618
C -2.56524763 1.92752907 -0.24800904
H 0.12454577 1.98773612 -2.02095711
H -1.48582196 1.66420582 -2.72211310
H -2.52106809 2.00814068 0.84081753
C -3.66867006 0.95412672 -0.61347014
C -5.68126848 -0.90000149 -1.17019185
C -5.00610061 1.27886180 -0.41133559
C -3.33500107 -0.31416019 -1.09138007
C -4.33474702 -1.23091271 -1.37559981
C -6.01212566 0.35405511 -0.68671786
H -5.27432213 2.25789112 -0.02630665
H -2.28977212 -0.57624281 -1.19376794
H -7.05789616 0.59065500 -0.53236141
C -0.94292681 -1.94771697 1.70828292
C -2.82683283 -0.02106650 2.41870923
C -2.30986784 -2.26021920 1.67177933
C -0.53315959 -0.68523932 2.10783258
C -1.47976988 0.27217492 2.48000967
C -3.24549085 -1.29843745 2.00580565
H -2.61800772 -3.24604121 1.34578732
H 0.51478480 -0.40958650 2.10793283
H -1.17756691 1.26487338 2.79365114
O 7.02335978 -2.13632741 0.18002744
O 6.72976907 0.59485605 0.65732152
O 2.27801416 1.25105571 1.80382728
O -4.13209086 -2.50109182 -1.83546747
O -6.65226487 -1.81809639 -1.42492795
O -4.59763599 -1.44384824 1.95067312
O -3.75170064 0.92844454 2.71755951
C -5.11051622 -2.69746554 1.53787396
H -4.86274430 -3.47146464 2.27099291
H -4.71711141 -2.97893088 0.55725002
H -6.18946020 -2.57643282 1.47076664
C -2.78899472 -2.88077752 -2.11425405

H -2.16895173 -2.83782664 -1.21515043
H -2.83797275 -3.90434788 -2.47996400
H -2.36346382 -2.23280917 -2.88668867
C 8.15724359 -1.73766988 -0.58637988
H 7.97086704 -1.91142986 -1.65056193
H 8.98363985 -2.36275862 -0.25386737
H 8.39677016 -0.68719191 -0.41621839
C 3.40112593 1.29012585 2.68338656
H 3.04525847 0.93193063 3.64867309
H 3.77348926 2.31157922 2.78871683
H 4.20121633 0.63731947 2.32771166
H 6.43657656 1.51144445 0.73168199
H -4.62080149 0.57039338 2.49208570
H -6.22637719 -2.59539398 -1.80670964

β''-β''' topC$_\gamma$-O

C 1.65518653 4.04866192 -1.25927167
C 2.25724433 1.99985450 0.52234751
C 0.70192529 3.64710215 -0.32492155
C 2.88338203 3.40389305 -1.33728394
C 3.18615523 2.37616015 -0.45535035
C 1.01511389 2.62095428 0.55935099
H 3.62664275 3.68637692 -2.07276198
H 0.29463270 2.29804584 1.30179988
O 4.44433788 1.80796326 -0.53583874
C 4.56125842 0.43009253 -0.54562301
C 4.97579525 -2.28708702 -0.37613412
C 5.74286302 -0.04066919 0.02771735
C 3.60850341 -0.42845001 -1.06312381
C 3.80488732 -1.80185675 -0.94975451
C 5.95387568 -1.41901493 0.09887203
H 5.15550917 -3.35246041 -0.27417198
H 2.70183493 -0.03023720 -1.50292977
C 2.69048545 -2.75937069 -1.32929814
O 1.97323330 -2.40590138 -2.43934620
C 0.44236750 -0.90151400 -0.50778362
C 1.10871758 -1.77800135 0.49895125
C 1.78041615 -3.02225859 -0.09865941
H 3.12593731 -3.74297313 -1.59700365
H -0.07384413 -0.00261759 -0.20355652
H 0.51401212 -1.09593218 -1.57267260
C 0.58292120 -3.95077617 -0.32423151
O -0.38212727 -3.59319339 0.66792651
C 0.09536996 -2.48297328 1.43034408
H 0.84144480 -5.00635436 -0.20952917
H 0.14976509 -3.79379640 -1.31708101
H 0.63741418 -2.86685683 2.30672063
H 1.81699415 -1.19020431 1.09173704
H 2.43218094 -3.45245836 0.66804840
H 1.43377351 4.86101902 -1.94432655
C -0.65552734 4.30967336 -0.29613216
O -1.28909491 4.02738066 0.94469291

C	-2.69054051	3.94658590	0.73876505
C	-2.82159747	3.26283053	-0.61366577
C	-1.65139493	3.85810842	-1.42016753
H	-0.51106541	5.39446219	-0.38182209
H	-3.11936928	3.37416938	1.56400998
H	-3.13568717	4.94999766	0.72011560
H	-3.78323576	3.43618413	-1.09621907
H	-1.95484517	4.71443056	-2.02349523
C	-1.22555512	2.67583285	-2.27675335
O	-1.34691289	1.55827478	-1.41821475
C	-2.46484020	1.76152398	-0.54555915
H	-0.19522459	2.71384409	-2.63124241
H	-1.89928415	2.57899916	-3.13862443
H	-2.09578577	1.52551830	0.45824263
C	-3.58374973	0.79427415	-0.87028561
C	-5.54199961	-1.14484770	-1.33561154
C	-4.92491040	1.07549668	-0.64453684
C	-3.21801948	-0.47897640	-1.32076722
C	-4.18438799	-1.44144681	-1.54001246
C	-5.90512289	0.11002525	-0.88678470
H	-5.22874186	2.04531035	-0.26730618
H	-2.16847025	-0.69779373	-1.46582789
H	-6.95528626	0.31713659	-0.72045951
C	-1.03955054	-1.60130724	1.88529488
C	-3.10151075	0.16247303	2.52911625
C	-2.36618548	-1.98771649	1.69782233
C	-0.75522470	-0.35902817	2.44523668
C	-1.78526436	0.52107444	2.76910554
C	-3.39256167	-1.11032343	2.02002349
H	-2.57469872	-2.96156146	1.27592603
H	0.27641006	-0.04876692	2.58241929
H	-1.58082470	1.50788343	3.17056608
O	7.07190375	-1.92838700	0.69230279
O	6.66205708	0.81976239	0.54044382
O	2.52354837	1.00370814	1.42376233
O	-3.94051259	-2.72775709	-1.93214000
O	-6.48366196	-2.10320525	-1.54502126
O	-4.72186474	-1.35303792	1.84995209
O	-4.10629389	1.04984300	2.76290588
C	-5.11167118	-2.66477067	1.48387607
H	-4.83292432	-3.37631937	2.26720318
H	-4.65595529	-2.96475164	0.53686038
H	-6.19245217	-2.63743169	1.36428357
C	-2.57777904	-3.12421673	-2.01998693
H	-2.06829096	-2.97875587	-1.06175085
H	-2.58777636	-4.18083759	-2.27902997
H	-2.06001164	-2.55936858	-2.80098267
C	8.28975597	-1.65455251	0.00377485
H	8.25090367	-2.07002385	-1.00772777
H	9.07691967	-2.14746340	0.57087916
H	8.48287111	-0.58185128	-0.04053166
C	3.49657395	1.33604312	2.41158241
H	3.59650385	0.46031965	3.05040645
H	3.15679163	2.19170495	3.00174459

H 4.46507723 1.56117364 1.95918856
H 6.35410767 1.71745186 0.36377550
H -4.92976209 0.63775876 2.46987300
H -6.03551603 -2.88558472 -1.88904149

β''-β''' topC_{α}-C_{Ph} α-side

C 0.99254658 3.87764960 -1.40471831
C 1.90119501 2.47718307 0.83318923
C 0.15368648 3.69516318 -0.30661001
C 2.25741417 3.30415321 -1.41327199
C 2.69427257 2.57850936 -0.31605259
C 0.62176155 3.02387297 0.81642198
H 2.90548334 3.36802769 -2.27902716
H -0.01949158 2.87809744 1.67587677
O 3.89404275 1.89291575 -0.40565286
C 3.79944133 0.51298959 -0.45322946
C 3.84776965 -2.24389730 -0.43939162
C 5.02749247 -0.14071388 -0.35803828
C 2.60937107 -0.18885330 -0.54889603
C 2.62686032 -1.57906685 -0.51348662
C 5.04493235 -1.53683648 -0.37253336
H 3.91127472 -3.32617811 -0.42488255
H 1.66366941 0.33044090 -0.62359822
C 1.30932464 -2.34104869 -0.57740809
O 0.18725203 -1.49181189 -0.38623756
C 0.06484809 -1.27966188 1.02324462
C 0.42541280 -2.63379967 1.66767651
C 1.16374017 -3.39585292 0.53239189
H 1.19711049 -2.78313492 -1.57236402
H 0.75581478 -0.49412336 1.34832174
H -0.95508592 -0.95769868 1.22102399
C 0.18059809 -4.51413513 0.15761131
O -0.65844780 -4.71348346 1.29307192
C -0.74189807 -3.52815872 1.95989237
H 0.65781624 -5.46547863 -0.07338390
H -0.44182380 -4.20223499 -0.68903057
H -1.26940784 -3.58565687 2.90198438
H 1.05979460 -2.48200153 2.54544100
H 2.11869067 -3.79963524 0.86251415
H 0.63997481 4.42121453 -2.27458920
C -1.29646444 4.07442648 -0.41326975
O -1.85450223 4.27963096 0.87125504
C -3.25206503 4.11229178 0.73170111
C -3.41168075 2.88376647 -0.16153346
C -2.17698001 2.97162940 -1.09763390
H -1.39162835 5.00062849 -0.99768013
H -3.68025722 3.99794930 1.72803819
H -3.69333880 4.99774231 0.25299118
H -4.35659345 2.88756902 -0.70326544
H -2.43110777 3.26593955 -2.11598769
C -1.61472038 1.55315602 -1.04182909
O -1.87114787 1.13350202 0.28884173

C -3.19796578 1.53896724 0.58964462

H -0.54383746 1.47070266 -1.22280542

H -2.14572647 0.90986074 -1.75530973

H -3.23205800 1.69231061 1.67261114

C -4.20515548 0.46532710 0.20304389

C -6.05870797 -1.52205293 -0.45227157

C -5.57022667 0.71623554 0.27553224

C -3.76109666 -0.80020186 -0.19248206

C -4.68216496 -1.78277371 -0.52395627

C -6.49622912 -0.27385867 -0.04722448

H -5.92838913 1.68968254 0.59478491

H -2.69683773 -0.99771696 -0.24295593

H -7.56245772 -0.09260930 0.00976157

O 6.21747178 -2.22388975 -0.24788170

O 6.18337376 0.56130932 -0.22221423

O 2.26593955 1.76443032 1.93056953

O -4.37672794 -3.04551122 -0.93903409

O -6.95215459 -2.48964106 -0.77815096

C -3.01083752 -3.42589592 -0.90556617

H -2.60860621 -3.33374402 0.10735149

H -2.97858983 -4.46736168 -1.21945120

H -2.41704746 -2.81104194 -1.58920921

C 7.12255866 -2.06368590 -1.33714271

H 6.65329951 -2.39728251 -2.26774040

H 7.98045256 -2.69514768 -1.11467337

H 7.44317773 -1.02542953 -1.43240871

C 3.59838164 1.90465243 2.41985666

H 3.53792317 1.78786742 3.50078581

H 3.99619386 2.89325980 2.18019809

H 4.25033783 1.13119880 2.00897774

H 5.96276358 1.50070260 -0.24040268

H -6.45477671 -3.28320402 -1.01039496

β''-β''' topC$_{\alpha}$-C$_{Ph}$ phenyl-side

C 1.27974544 -1.71944302 0.00028561

C 0.56524598 0.90280970 -0.00012778

C -0.07046909 -1.44052601 0.00011263

C 2.28585477 -0.79257049 -0.00016297

C 1.90525833 0.55648930 -0.00018395

C -0.42685588 -0.09393581 -0.00000721

H -0.81439462 -2.22690055 0.00060901

H 3.33209001 -1.07150412 -0.00047738

H 2.64260346 1.35037661 -0.00051952

O -1.70210334 0.39153052 0.00047518

O 0.20615846 2.21238988 0.00028944

C -2.75849885 -0.54875395 -0.00037006

H -2.71922322 -1.17717379 0.89405468

H -2.71770760 -1.17724661 -0.89460310

H -3.68042572 0.02770010 -0.00134732

H -0.75706750 2.25896677 -0.00111091
